# Which Metrics Best Capture Protein Structural Changes in Molecular Dynamics Simulations? Evaluating Score Combinations and Force-Field Effects

**DOI:** 10.1021/acs.jcim.6c01218

**Published:** 2026-08-26

**Authors:** Christian Pfaendner, Thilo Paul, Benjamin Unger, Kristyna Pluhackova

**Affiliations:** † Stuttgart Center for Simulation Science, Cluster of Excellence EXC 2075, 9149 University of Stuttgart, Universitätsstr. 32, Stuttgart 70569, Germany; ‡ Artificial Intelligence Software Academy, University of Stuttgart, Stuttgart 70569, Germany; § 150232 Institute for Applied and Numerical Mathematics, Karlsruhe Institute of Technology, Englerstr. 2, Karlsruhe 76131, Germany

## Abstract

The scores RMSD, TM-score, GDT, lDDT, SphereGrinder, CAD, QCS, FlexE, and MolProbity provide an automated, comprehensive assessment of conformational changes and structural quality of proteins and thus represent a promising tool for routine use in molecular dynamics (MD) simulations, particularly in high-throughput settings. However, it remains unclear to what extent these scores provide redundant information and which scores are most informative for capturing conformational changes. Based on MD simulations of 268 diverse proteins, we demonstrate that the investigated scores are highly correlated and that one global score (or QCS), one local score, and FlexE capture almost 90% of the variance across all scores. Since MD randomness partially explains FlexE’s variability, we argue that a combination of one global and one local score is sufficient for most practical applications. We also investigate the influence of simulation setups and find that the choice of the force field can significantly affect scoring results. In particular, the setup using the Amber ff19sb force field yields systematically different scores than all CHARMM36m-based setups, emphasizing the importance of methodological choices when designing MD experiments and interpreting their results.

## Graphical abstract

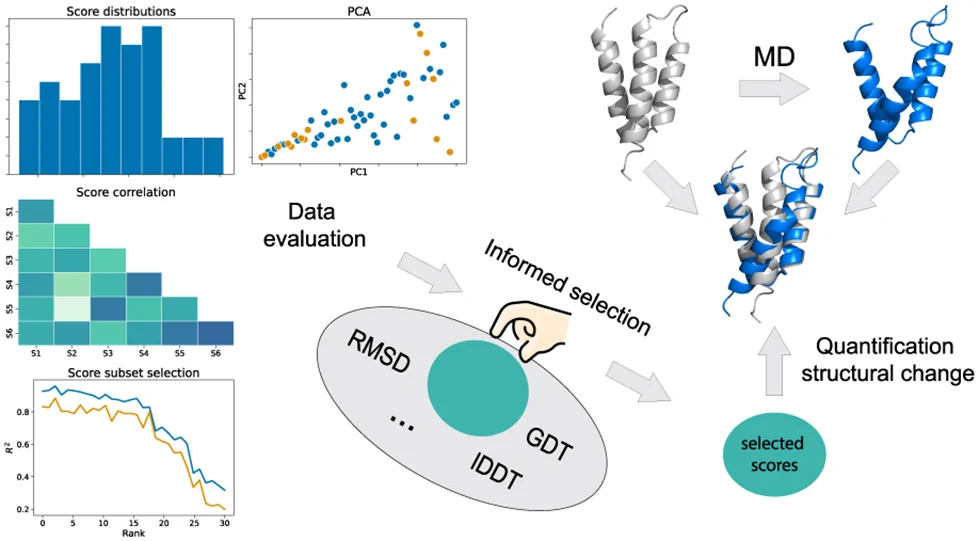

## 1 Introduction

Proteins have been studied by molecular dynamics (MD) simulations for over four decades,^1^ providing invaluable insights into protein dynamics and structure-function relationships^2^ in countless cases. For example, pore formation of Gasdermin D and A3,^3^ protein folding,^4^ activation of G-protein coupled receptors,^5^ and water passage through aquaporines.^6,7^ In many MD frameworks, protein conformations at various simulation times are routinely compared to their experimentally resolved or AI-generated starting conformations to assess structural changes.^8–14^ These structural comparisons can be performed visually, mainly as a sanity check^15^ or on a sample basis, as it quickly becomes infeasible, particularly for high-throughput studies. Therefore, a better alternative or extension to visual inspection is an automated and reproducible assessment via quantitative scores^8–13^ like the root-mean-square deviation (RMSD).^16^

Over the years, a variety of conceptually distinct scores have been developed to compare and evaluate protein structures.^16–26^ Fundamentally, they can be categorized into superposition-based or superposition-free, depending on whether two structures are spatially aligned by optimizing a given objective function, and into global or local, depending on whether entire structures are compared or substructures are considered separately.^27^ Moreover, protein structures can be evaluated individually, that is, without a reference structure, by quantifying deviations of intrinsic structural features such as bond lengths from expected values.

In this study, we focus on the scores RMSD,^16^ the template modeling score (TM-score),^17^ the global distance test (GDT)^18,19^ , the local distance difference test (lDDT),^20^ SphereGrinder,^21^ the quality control score (QCS),^22^ the contact area difference (CAD),^23^ FlexE,^24^ and MolProbity.^25,26^ Each score is briefly presented and categorized in Table 1, while a detailed description is given in Supporting Information Section S1. We selected these scores because all of them were extensively used in the field of protein structure prediction, for example, in the CASP experiments^28,29^ , which are large, community-wide competitions evaluating how well computational methods can predict 3D protein structures. Moreover, they played a major role in showcasing and validating breakthroughs such as AlphaFold.^30^ Finally, they cover all of the methodological categories introduced earlier and assess a broad range of structural aspects, e.g., interatomic distances or secondary structure elements (SSEs).

**Table 1:** Summary of Investigated Scores^a^

Score	Description	Reference	Superposition	Scope	Range
RMSD^16^	Average distance between atoms of the reference and the query structure	Yes	Yes	Global	[0, ∞)
TM-score^17^	Average distance between atoms of the reference and the query structure normalized by a length-dependent factor	Yes	Yes	Global	[0, 1]
GDT^18,19^	$\frac{1}{4}\left(F_{1}+F_{2}+F_{4}+F_{8}\right)$ where *F*_*d*_ is the maximum fraction of atoms of the query structure that can be superimposed onto the reference within a distance of *d* Å	Yes	Yes	Global	[0, 1]
lDDT^20^	$\frac{1}{4}\left(F_{0.5}+F_{1}+F_{2}+F_{4}\right)$ where *F*_*d*_ is the fraction of atom distances that differ by at most *d* Å between reference and query structure	Yes	No	Local	[0, 1]
SphereGrinder^21^	$\frac{1}{2}\left(F_{2}+F_{4}\right)$ where *F*_*d*_ is the fraction of local environments of the query structure that can be superimposed onto the reference with an RMSD smaller than *d* Å	Yes	Yes	Local	[0, 1]
QCS^23^	Preservation of SSEs between reference and query structure	Yes	No	Local	[0, 1]
CAD^23^	Similarity of residue–residue contact areas between reference and query structure	Yes	No	Local	[0, 1]
FlexE^24^	Energy required to deform the reference into the query structure based on elastic network model	Yes	No	Global	[0, ∞)
MolProbity^25,26^	Evaluation of clashes and comparison of bonds, angles, and dihedral angles to expected values	No	No	Local	[0, ∞)

a The column range specifies the possible output of the software used, except for QCS, which is rescaled from [0, 100] to [0, 1] in our work for better visual compatibility with the other scores. For all reference-based scores, except RMSD and FlexE, higher values indicate more similar structures. For MolProbity, lower values indicate greater agreement with expected values.

Although the selected scores are different by design, a study by Olechnovič et al. showed for a slightly different set of scores that they are highly correlated with Spearman’s rank correlation coefficients of 0.7 or higher based on data from CASP10–12.^31^ This raises the following research questions that we answer throughout this study: Are correlations between scores equally high for MD-generated structures as for those predicted by machine learning models or traditional physics- and geometry-based methods? Can a single score, or a combination of a few scores, sufficiently capture the most relevant information contained in all nine studied scores, even though each assesses structural aspects of proteins differently? And finally, how significant is the choice of the force field—CHARMM36m vs Amber ff19sb—and its parameters, in particular the treatment of van der Waals (vdW) interactions and the water model, on the protein conformations after MD simulations, and thus the scoring results? To address these questions, we conducted 1 μs MD simulations of 268 diverse proteins and investigated similarities and redundancies between scores based on the final snapshots. Furthermore, we simulated a subset of 31 proteins with four commonly used simulation setups (see Section 2.2), and examined systematic score differences between them. These setups may differ in one, two, or three of the following parameters: the force field itself, the treatment of long-range vdW interactions, because it was found to have a substantial influence on the outcome of MD simulations,^32,33^ and the water model, as it influences thermodynamic properties of biomolecules,^34^ and in some cases this influence is stronger than that of the biomolecular force field itself.^35^

## 2 Methods

An overview of the used protein data sets, simulation setups, and scoring strategy is displayed in Figure 1 and described in the following subsections. A brief description of the simulation protocol is also given.

**Figure 1:**
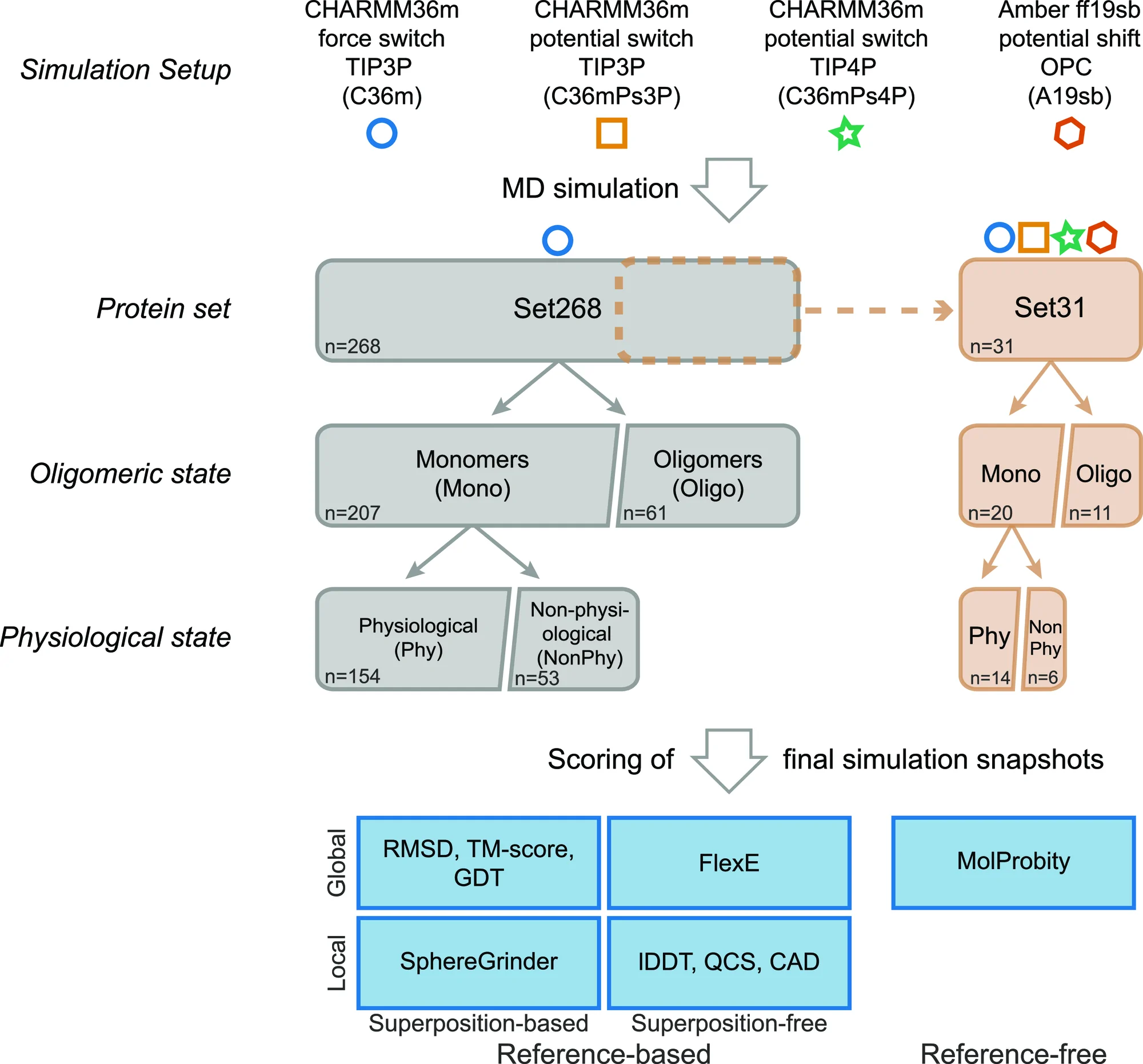
Overview of the generated protein data sets and their subsets, the utilized simulation setups, and the applied scores. The number of proteins per data set is indicated in the lower left corner of each rectangle. Simulation setups are represented by unique colored symbols, which indicate how each protein data set was simulated.

### 2.1 Protein Data Set Acquisition

We downloaded 207 proteins with a resolution of 2 Å or better, comprising 50 to 250 residues, and containing diverse secondary structure content from the RCSB database.^36^ The distributions of their properties are visualized in Supporting Information Figure S1. Subsequently, we created their homooligomeric structures if a higher oligomeric state was indicated by the “author determined biological unit” or the “software determined quaternary structure” field of remark 350 in the corresponding PDB files. This resulted in 61 additional oligomers, and thus a total set of 268 proteins, which we abbreviate as Set268. This approach inherently led to the inclusion of 53 monomers that are nonphysiological, i.e., neither the author(s) nor the software annotates the protein as monomeric. We included these nonphysiological monomers to obtain also large conformational changes and thus to ensure coverage of the full score ranges, as their nonphysiological nature is expected to produce worse scores. Note that the discrepancy between the 53 nonphysiological monomers and the 61 oligomers stems from differing oligomeric-state annotations of the author(s) and the software. Moreover, proteins that naturally function as components of larger protein complexes are simulated in isolation, which may reduce their stability during the simulations. Similar to nonphysiological monomers, these proteins are expected to undergo larger conformational changes. However, this does not affect the primary objectives of our study, as our analysis focuses on the comparative performance of scores rather than on absolute score values or the exact extent of structural deviations from the initial conformations. Further details on the protein selection process and the generation of the oligomers are given in Supporting Information Section S2.

We manually selected a subset of 31 proteins, denoted Set31, from Set268. The 31 proteins were selected to maximize differences in their properties (see Supporting Information Figure S1), and they consist of 20 monomers (14 physiological and six nonphysiological) and 11 homooligomers.

The PDB identifiers of all studied proteins, together with their properties and allocations to the data sets Set268 and Set31, are given in the supplementary file set268_set31_properties.csv. Moreover, the alignments to their respective UniProt^37^ sequences are provided in the supplementary file uniprot_alignments.csv.

### 2.2 Simulation Setups

We conducted all-atom MD simulations using GROMACS2023 ^38,39^ with the four simulation setups

- **C36m:** CHARMM36m;^40–42^ force-switch vdW modifier;^43,44^ CHARMM TIP3P water model^45,46^

- **C36mPs3P:** CHARMM36m; potential-switch vdW modifier;^43,44^ CHARMM TIP3P water model
- **C36mPs4P:** CHARMM36m; potential-switch vdW modifier;^43,44^ TIP4P water model^45^
- **A19sb:** Amber ff19sb;^47,48^ potential-shift vdW modifier;^43,44^ OPC water model.^49^

Background information on the used force fields, vdW modifiers, and water models, including their strengths and weaknesses are provided in Supporting Information Section S3.3. Set268 was simulated only with C36m, while Set31 was simulated with all four simulation setups. Note that this set of simulation setups examined is not exhaustive. Instead, it focuses on force-field parameters that are in line with the recommendations of the force-field developers and commonly applied by the simulation community. Our objective is to determine whether systematic differences between scores emerge among these standard settings, rather than to pinpoint the exact origins of any observed discrepancies. Furthermore, our approach remains practically feasible, as each simulation setup requires 31 additional simulations.

### 2.3 Simulation Protocol

To ensure methodological consistency, the following sequential steps were conducted in the same manner for each protein downloaded from the RCSB database: first, crystallographic water molecules were removed, retaining only the protein. Next, missing heavy atoms and hydrogen atoms were added, and protonation states were assigned to terminal residues. Subsequently, a dodecahedron simulation box with 1.5 nm distance to the protein was added, and 100 steps of energy minimization (EM) were conducted in vacuum. Each system was then solvated, and three rounds of EM were carried out with the protein frozen (500 steps), the backbone frozen (1000 steps), and without restrictions (1000 steps). For further equilibration, 1 ns position restraint simulations were conducted, once with all heavy atoms restrained and once with the backbone restrained. Finally, each system was simulated for 1 μs. A detailed description of the simulation protocol and the corresponding parameters is provided in Supporting Information Section S3.1 and S3.2, respectively.

To provide practical guidance regarding computational cost of the production MD simulation, we assessed relative GROMACS runtimes on one NVIDIA A40 GPU and 16 CPU cores of an AMD EPYC Milan (Zen 3) processor in ns/d, and found that C36m was on average approximately 5%, 52%, and 17% faster than C36mPs3P, C36mPs4P, and A19sb, respectively. These relative runtimes should be interpreted as approximate performance estimates rather than exact measurements since simulations were not conducted under controlled benchmark conditions.

### 2.4 Scoring

For each simulation, the protein was made whole via the GROMACS command gmx trjconv using the option -pbc cluster. Subsequently, the final snapshot was extracted, hydrogens were removed (except for MolProbity), and terminal residues were discarded. Afterward, the final snapshot was scored, that is, it was compared to the corresponding starting structure (i.e., crystal structure) via RMSD, TM-score, GDT, lDDT, SphereGrinder, QCS, CAD, and FlexE. For GDT, we additionally extracted and scored snapshots every 10 ns (only Set268 and C36m), discarding the first 200 ns as equilibration. For RMSD and FlexE, lower values indicate greater similarity to the crystal structure, whereas for all other scores, higher values indicate greater similarity. We therefore use the terms high-similarity and low-similarity values throughout this article, according to each score’s intended direction. In addition to reference-based scores, the structural quality of the final snapshot and the starting structure, specifically bond, angle, and dihedral angle distributions, as well as clashing atoms, was assessed via MolProbity. For oligomers, scoring was performed individually for each chain, and the final score was calculated as their arithmetic mean. Note that QCS is computed as an average over six subscores, some of which are defined only for proteins with at least two or three SSEs. To avoid artificially low QCS values, as the subscores are set to zero by default if they are not defined, we calculate QCS only for the 236 proteins with at least three SSEs.

## 3 Results

### 3.1 Is a Single Score Sufficient? Similarity and Redundancy Analysis of Scores

In this subsection, we investigate whether a single score is sufficient to represent the structural differences captured by all examined scores based on the final snapshots of 268 proteins (Set268) simulated for 1 μs. To this end, we analyze (i) score distributions (Section 3.1.1), (ii) correlation between scores (Section 3.1.2), (iii) principal components (Section 3.1.3), and (iv) the predictive power of score subsets (Section 3.1.4).

#### 3.1.1 Score Distributions

All reference-based score distributions (see Figure 2) are unimodal and overall exhibit a similar shape, characterized by a steep flank toward high-similarity and an elongated tail toward low-similarity values, which is more pronounced for the global superposition scores RMSD, TM-score, and GDT. While most of the scores are defined on the interval [0,1], their distributions differ in the location of their peak and how they spread across that range. For example, TM-score ranges from approximately 0.2 to 1.0, with the peak at around 0.9, while lDDT ranges from approximately 0.4 to 0.9, with the peak at around 0.7. The 53 nonphysiological monomers contained in Set268 yielded, as expected, considerably more low-similarity scores, which is particularly evident from the shifted cumulative distribution functions displayed in Figure 2 (highlighted as brown lines). Consequently, subsequent analyses also consider structures that have deviated strongly from the starting conformation, rather than only marginally. Nonetheless, at least half of the nonphysiological monomers exhibit score values near the peak of each corresponding histogram across all scores, suggesting that simulating proteins in nonphysiological oligomeric states does not inherently yield low-similarity score values. One reason is that certain proteins may be structurally stable and even functional as both monomers and oligomers, as demonstrated, for example, for certain aquaporines.^50^ Moreover, in-solution-stable monomers may be stable precursors of functional oligomers.^51,52^

**Figure 2:**
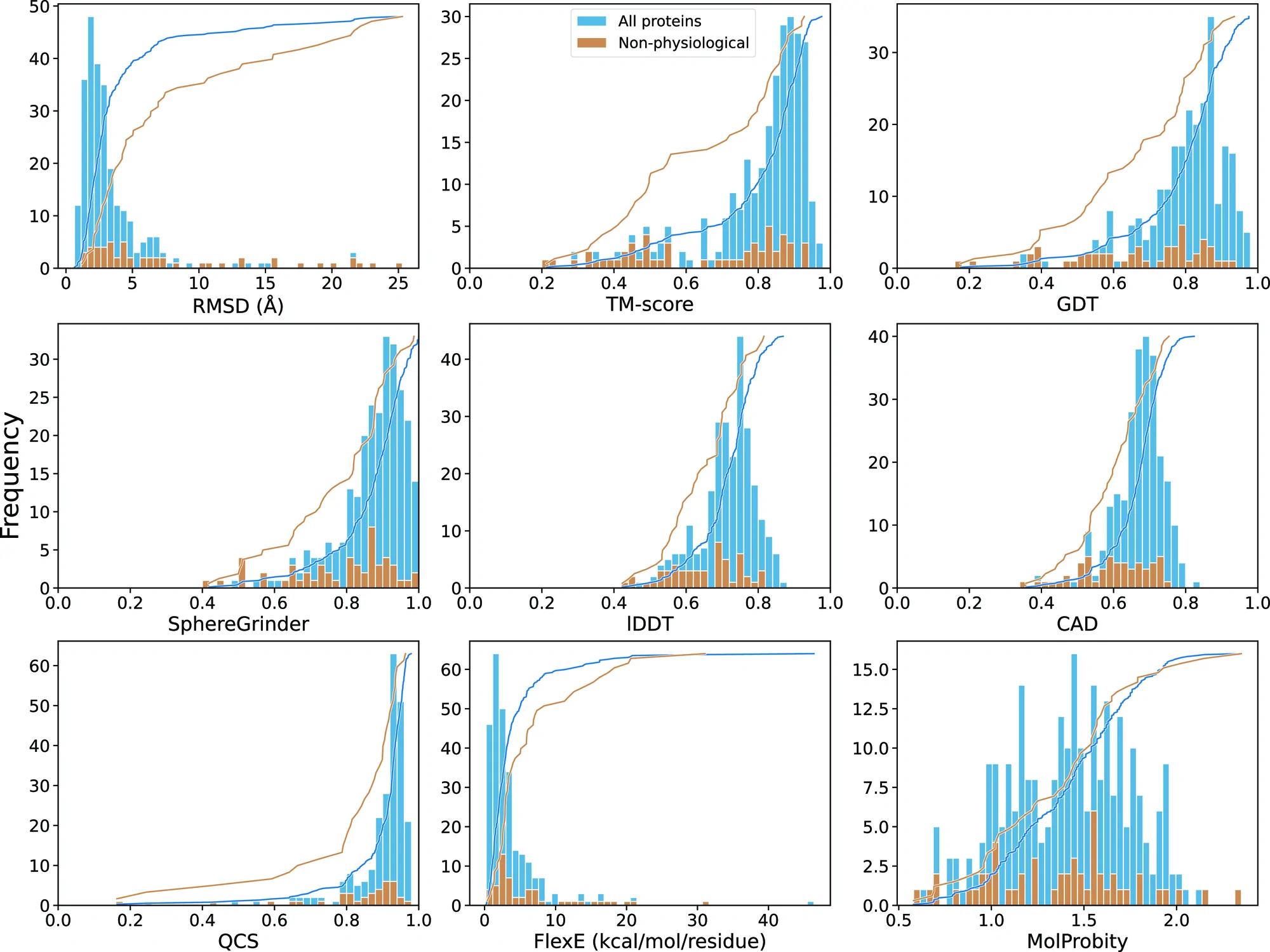
Score distributions. The histograms for all 268 proteins are shown in blue, and the subset of 53 nonphysiological monomers is displayed in brown. Cumulative distribution functions are depicted as thin lines in the same colors.

The MolProbity distribution is, in contrast to other scores, unimodal, symmetric, and roughly bell-shaped. Based on its quantile–quantile plot (see Supporting Information Figure S2), it appears approximately normal. The corresponding values range from 0.58 to 2.35, without any obvious outliers, indicating that the final snapshots are of high to excellent quality. Moreover, no obvious systematic overrepresentation of low-quality values exists for the nonphysiological monomers. Thus, the nonphysiological monomers appear to have structural quality comparable to that of physiological proteins after simulation, even though their secondary and tertiary structure tend to deviate more strongly from the starting structure on average.

Since MolProbity evaluates protein structures individually, we additionally analyzed the corresponding starting structures (i.e., crystal structures) of the MD simulations (see Supporting Information Figure S3). Overall, the MolProbity distribution of the crystal structures is quite similar to that of the final snapshots, though slightly broader, with values ranging from 0.50 to 3.46 and a single outlier at 4.57. When comparing the MolProbity subscores, the final snapshots exhibit fewer clashes than the crystal structures, most likely because they are eliminated during energy minimization and system equilibration. Additionally, the final snapshots exhibit better Rama-Z scores but worse RMSZ scores, and contain more Ramachandran outliers and fewer favorable Ramachandran compared to the crystal structures. This is expected because MD naturally introduces motion and desirable conformational diversity.

For the interpretation of absolute score values, it is important to also examine whether scores are significantly correlated with protein properties, as proteins with certain properties might systematically yield higher or lower score values. Therefore, we computed the corresponding Pearson correlation coefficients between scores and protein properties, and found that most coefficients are close to zero, except for slight to moderate correlations between TM-score and the number of residues (*r* = 0.34), MolProbity of the crystal structures and the resolution at which they were resolved (*r* = 0.34), and MolProbity of the final snapshots and the secondary structure content (helix: *r* = −0.41, beta: *r* = 0.28, unstructured: *r* = 0.17 after removal of proteins with corresponding SSE content of zero). Note that we excluded nonphysiological monomers here as their scores are systematically lower and could therefore bias this assessment. A complete and in-depth analysis of the correlation between scores and protein properties is provided in Supporting Information Section S4.

To summarize, the shapes of the distributions are highly similar for reference-based scores, although they may cover different absolute values. The MolProbity distribution appears, in contrast to other scores, approximately normal and indicates that the simulated structures are of high quality. Finally, scores are uncorrelated with protein properties, aside from slight to moderate correlation observed for FlexE and MolProbity.

#### 3.1.2 Correlation between Scores

The scores used in this study evaluate proteins from different perspectives. To understand their pairwise relationships and the extent to which they convey redundant information, we computed Pearson correlations between all scores (see Figure 3a) with reversed signs for correlations involving RMSD, FlexE, or MolProbity to ensure a consistent interpretation: positive correlation coefficients indicate that higher-similarity values (higher-quality for MolProbity) in one score tend to be associated with higher-similarity values in the other score, and vice versa. Additionally, we generated score pair plots, representative examples of which are shown in Figure 3b, with the full set provided in Supporting Information Figure S6.

**Figure 3:**
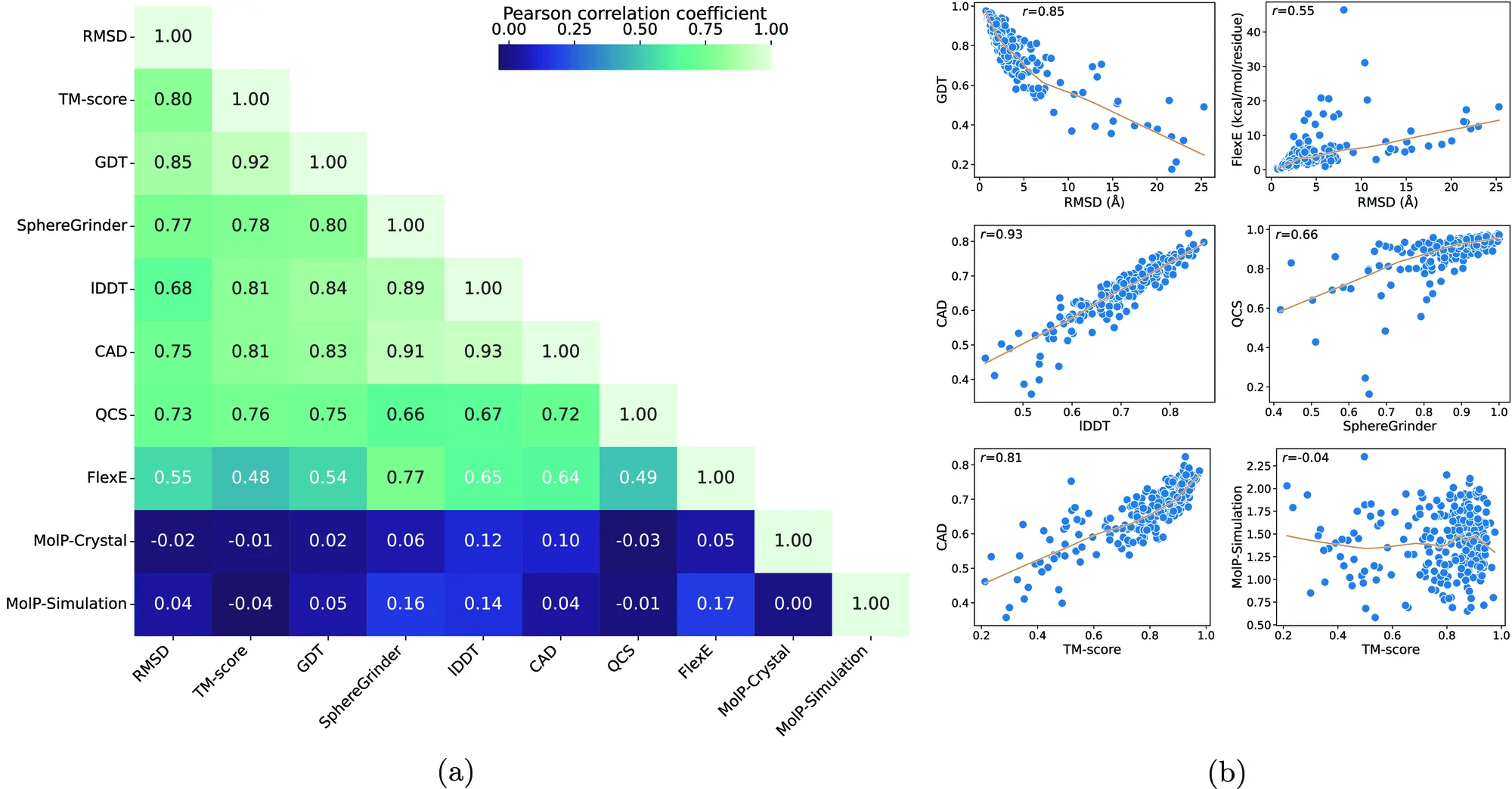
(a) Pearson correlation matrix of all scores. The signs of correlations are adjusted so that positive correlation coefficients correspond to higher-similarity (higher-quality for MolProbity) values in one score tend to be associated with higher-similarity values in the other score, and vice versa. (b) Exemplary pair plots between scores. Proteins are depicted as blue spheres, correlation coefficients are given in the upper left corners, and orange lines provide smoothed estimates of the general trend by fitting linear local weighted regressions.^55^ MolP: abbreviation for MolProbity.

The correlations among reference-based scores are high, ranging from 0.66 to 0.93. FlexE represents an exception, as it exhibits a strong correlation with SphereGrinder (*r* = 0.77), but only moderate correlations with the remaining reference-based scores (*r* = 0.48 to 0.65). This can partly be attributed to the two potential outliers 2D8E and 3MX7 with exceptionally high FlexE values of 31.1 and 46.4, respectively. Their exclusion indeed increases the correlations involving FlexE by 0.04 to 0.09, aside from the one with QCS (Δ*r* = −0.05).

Visual inspection of the pair plots indicates that some reference-based scores have a nonlinear relation because their scattered data points exhibit a slight L- or sigmoid-shape (e.g., RMSD vs GDT or TM-score vs CAD in Figure 3b). Moreover, most pair plots exhibit nonconstant variance across the score ranges, suggesting a heteroscedastic pattern. The Pearson correlation coefficients, which measure linear dependence, might therefore be biased estimations of the true association strength between scores. Hence, we also computed the Spearman’s rank correlation coefficients (see Supporting Information Figure S7), which measure the strength of monotonic relationships and are usually less susceptible to outliers and distributions with heavy tails.^53,54^ Although Spearman’s rank and Pearson correlation coefficients are not directly comparable, a relatively large or systematic change can indicate a nonlinear component. Such a systematic change was observed only for FlexE, with Spearman’s rank correlation coefficients higher by 0.10 to 0.26 than the corresponding Pearson correlation coefficients. This change is quite significant, considering that the mean absolute difference between the Pearson and Spearman’s rank correlation coefficients involving other reference-based scores is only 0.03. Considering this adjustment for FlexE, all evaluated reference-based scores are highly correlated, suggesting that they assess structural changes occurring during MD similarly.

The remaining score, MolProbity, exhibits a distinct pattern, showing no meaningful correlation with any other score for the crystal structures and the final simulation snapshots. Specifically, Pearson correlation coefficients are close to zero, and the data points appear randomly scattered in the pair plots. This is likely due to MolProbity being the only score in our study that directly assesses the geometric quality of proteins instead of capturing the structural change relative to a reference structure. Hence, a conformational change that affects reference-based scores may not influence the MolProbity score at all. In addition, MolProbity of the crystal structure is uncorrelated with MolProbity of the final snapshots. Thus, the quality of the starting structure seems to have no impact on its quality after simulation.

#### 3.1.3 Principal Component Analysis of Reference-Based Scores

In the previous section, the reference-based scores appeared to be highly correlated. To complement this pairwise and local view on dependence, we additionally performed a principal component analysis (PCA), which quantifies how much information in terms of variance is shared across all scores. Prior to PCA, the scores were preprocessed by reversing the signs of RMSD and FlexE, standardizing to a mean of zero and variance of one, and estimating missing QCS values with scikit-learn’s KNNImputer based on the two nearest neighbors. MolProbity is excluded here because it showed no meaningful correlation with any of the other scores, and a PCA conducted with MolProbity revealed that it almost exclusively contributes to the second principal component (PC) (see Supporting Information Figure S8). This suggests that the geometric quality MolProbity quantifies is a unique piece of information not present in the other scores.

We begin by assessing how much variance each principal component captures. As shown in Figure 4a, the first PC explains a predominant proportion of the total variance (78.9%), the second PC captures a considerably smaller amount (8.9%), and the remaining PCs contribute only marginally (4.8% to 0.5%). Together, the first two components account for almost 90% of the total variance, indicating that the data are effectively low-dimensional.

**Figure 4:**
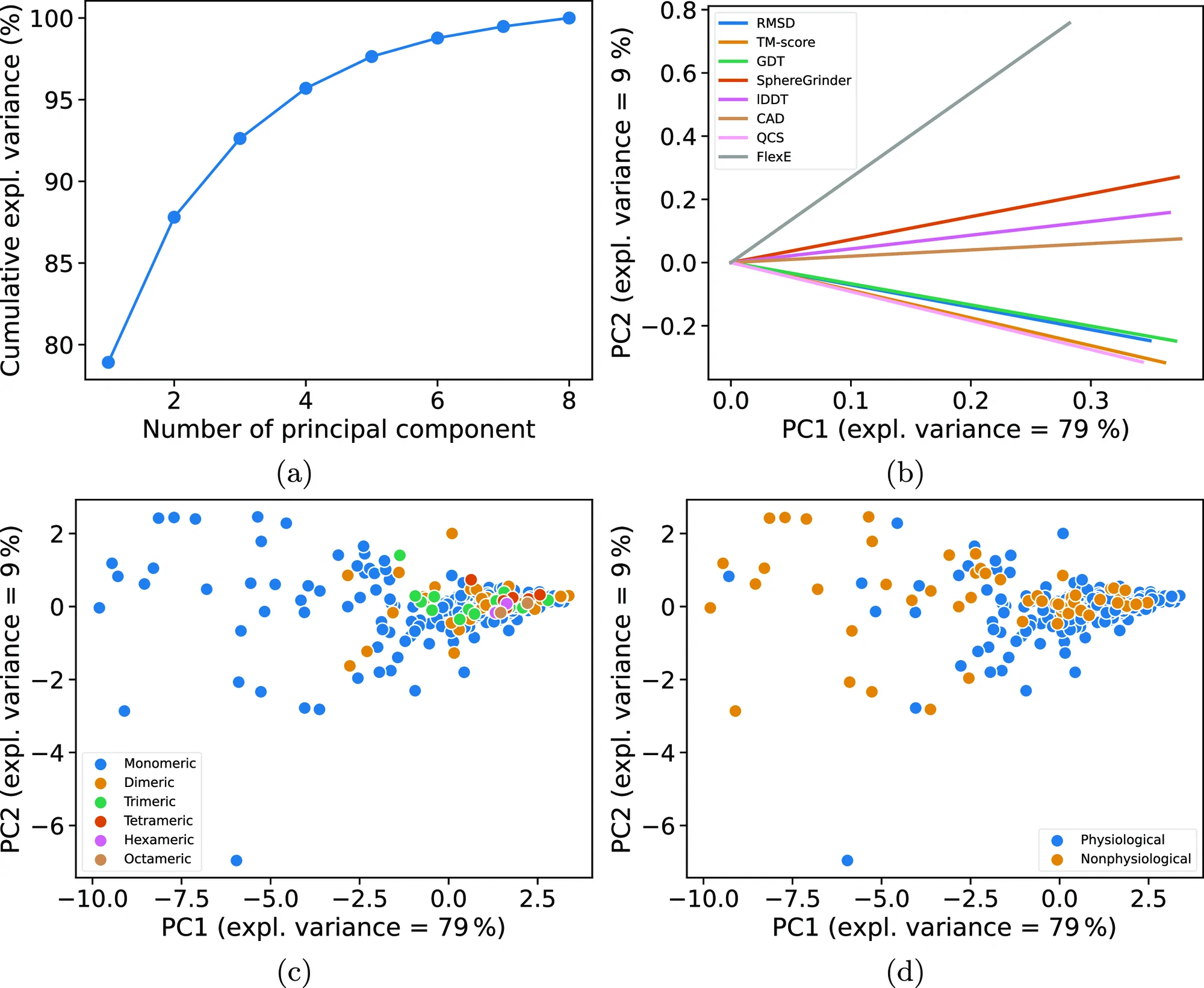
PCA of reference-based scores. (a) Cumulative explained variance of each PC. (b) PCA loadings plot of the first two PCs. (c)–(d) Scores projected onto the first two PCs with proteins colored by oligomeric state (c) or by physiology (d).

Although the first two PCs capture most of the total variance, it does not indicate how each score contributes to these PCs. In general, each PC is a weighted combination of the original scores, and these weights, called loadings, specify whether the scores contribute equally or whether some have significantly larger contributions than others. To assess this visually, we plotted the corresponding loadings for the first two PCs in Figure 4b.

All scores contribute equally to the first PC with loadings between 0.34 and 0.37, except for FlexE, which has a slightly lower loading of 0.28. Consequently, the first PC mainly captures variance that is shared, and thus redundant, among all scores. The loadings for the second PC are more diverse, and three clusters can be identified: First, QCS and the global scores RMSD, GDT, and TM-score with negative loadings between −0.32 and −0.25. Second, the local scores SphereGrinder, lDDT, and CAD with positive loadings between 0.07 and 0.27, and finally, FlexE with the largest absolute loading of 0.76. Thus, FlexE contributes most to the second PC, and the remaining two score clusters contribute with similar magnitude but opposite signs. One possible explanation why QCS groups with global scores is that the locality of QCS may be protein-specific. For example, when a protein comprises only three SSEs (minimum to compute all subscores), each element represents a substantial fraction of the overall structure. Moreover, the handedness subscore evaluates the three SSEs simultaneously, thereby providing a global assessment of the protein rather than a strictly local one.

For the third PC, data not shown, the loadings get more diverse without obvious clustering, ranging from −0.53 to 0.55. This indicates some score-specific contribution to the total variance. However, whether this reflects meaningful information or noise can not be determined from PCA alone and may be application-specific. Note that the signs of the loadings are arbitrary and can be reversed without affecting the interpretation.

In the last step, we projected the data onto the first two PCs, and highlighted the oligomeric state and the nonphysiological monomers to identify potential clusters (see Figure 4c and 4d). However, no clearly distinguishable clusters are observed. Instead, the projection resembles the score pair plots involving FlexE (see Figure 3b upper right and Supporting Information Figure S6), which might be due to the equal contributions of all scores to the first PC and FlexE having the largest impact on the second PC.

In summary, the first two PCs account for almost 90% of the total variance. Among them, only the second PC shows distinct contributions from global scores (including QCS), local scores, and FlexE, as reflected by their respective loadings. Thus, it is interesting to hypothesize here that a few scores, ideally one score from each category, could be sufficient to capture most of the structural differences between the starting and final simulation snapshots.

#### 3.1.4 Selection of Optimal Score Combinations

To test the hypothesis that only a few scores can account for the majority of structural differences between the final and starting simulation snapshots, we address the following question: Given a small subset of scores, how accurately can we predict the remaining scores? Specifically, we aim to identify individual scores, score pairs, and score triplets, hereafter collectively referred to as score combinations, that best predict the remaining scores using an ordinary least-squares (OLS) model. In this context, best refers to model performance evaluated by the coefficient of determinations $R_{\mathrm{pred}}^{2}$ and $R_{\mathrm{all}}^{2}$ defined as

$$\begin{array}{c} R_{\mathrm{pred}}^{2}=1-\frac{\mathrm{RSS}}{\mathrm{TSS}}=\frac{∥Ŷ{∥}_{F}^{2}}{∥Y{∥}_{F}^{2}},\\ R_{\mathrm{all}}^{2}=1-\frac{{\mathrm{RSS}}_{\mathrm{all}}}{{\mathrm{TSS}}_{\mathrm{all}}}=\frac{∥\mathrm{X̂}{∥}_{F}^{2}}{∥X{∥}_{F}^{2}}, \end{array}$$

where RSS and TSS denote the residual and total sum of squares, respectively. *Y* and *Ŷ* represent the true and predicted values of the remaining scores. Likewise, *X* contains all score values, while *X̂* = [*X*_*S*_, *Ŷ*] is composed of the values of the current score combination and of the predicted remaining scores. While $R_{\mathrm{pred}}^{2}$ and $R_{\mathrm{all}}^{2}$ are similar per definition, they have different interpretations: $R_{\mathrm{pred}}^{2}$ quantifies the variance in the remaining scores while $R_{\mathrm{all}}^{2}$ assesses the variance in all scores that can be predicted from and explained by the current score combination. We focus, as before, on reference-based scores and report both variants of *R* ^2^ for the training set (train) and for 10-fold cross-validation (10-fold) to assess predictive performance more realistically. Further technical details, including a step-by-step description and a derivation of the coefficients of determination, are provided in Supporting Information Section S5.

##### 3.1.4.1 Performance Comparison of Score Combinations

We ranked score combinations by each *R* ^2^ variant and separately for individual scores, pairs, and triplets (see Figure 5). Three qualitative aspects immediately stand out: first, for identical ranks, performance metrics are typically ordered according to $R_{\mathrm{all}}^{2}\left(\mathrm{train}\right)>R_{\mathrm{all}}^{2}\left(10‐\mathrm{fold}\right)>R_{\mathrm{pred}}^{2}\left(\mathrm{train}\right)>R_{\mathrm{pred}}^{2}\left(10‐\mathrm{fold}\right)$ . Second, all metrics exhibit similar trends across individual scores, pairs, and triplets, characterized by a slight linear decline over most ranks and a sharp decline between the fifth-lowest-ranked and lowest-ranked score combination. Finally, for identical ranks, score triplets outperform score pairs, and both outperform individual scores across all performance metrics. The final finding is consistent with the PCA results presented in the previous Section 3.1.3, as each score contributes unique variance to PC3 and partly PC2 that is not shared with the remaining scores. Assuming that this unique variance is also relevant to predict the remaining scores, adding scores should increase the predictive performance. However, it does not imply that combinations with more scores are always performing better than combinations with fewer scores. In fact, the best individual score performs better than the worst score pair, and the best score pair performs better than the worst score triplet across all coefficients of determination.

**Figure 5:**
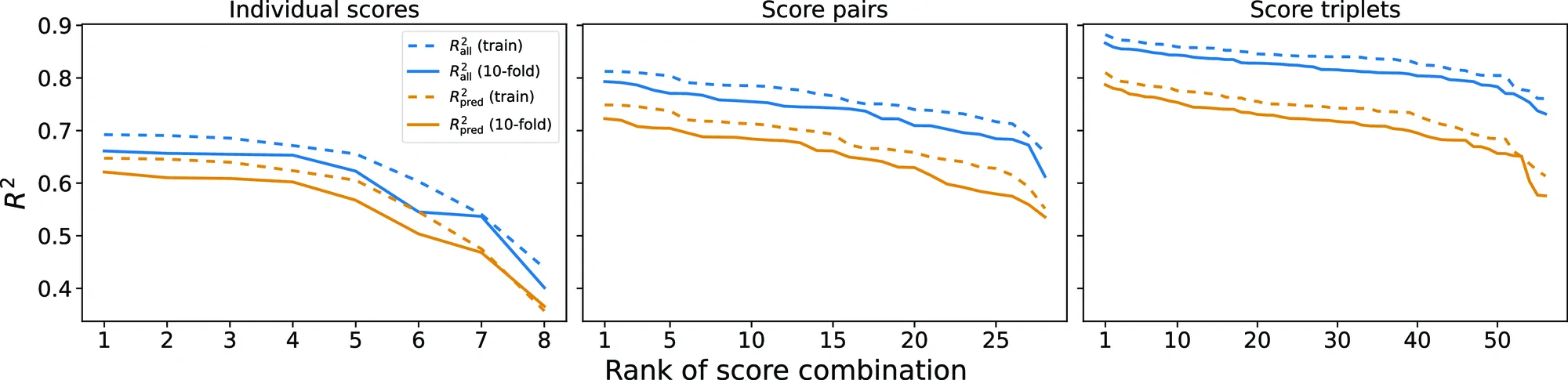
Predictive performance of score combinations. Score combinations are ranked in descending order based on $R_{\mathrm{all}}^{2}$ and $R_{\mathrm{pred}}^{2}$ . Both variants were assessed on the training set (train; dashed lines) and with 10-fold cross-validation (10-fold; solid lines).

Quantitatively, $R_{\mathrm{all}}^{2}$ (train), ranges from 0.69 to 0.44, 0.81 to 0.66, and 0.88 to 0.76 for individual scores, pairs, and triplets, respectively, and $R_{\mathrm{pred}}^{2}$ (train) from 0.65 to 0.36, 0.75 to 0.55, and 0.81 to 0.61, respectively. The corresponding 10-fold cross-validation values are lower by 0.02 to 0.03, indicating that the OLS models generalize well to unseen data. Notably, these values of $R_{\mathrm{all}}^{2}$ can be compared directly to the PCA results, as both quantify the fraction of total variance captured. Specifically, in Supporting Information Section S5 we prove that $R_{\mathrm{all}}^{2}$ is bounded above by the explained variance of the PCA for the OLS model. That is, for individual scores, the highest achievable $R_{\mathrm{all}}^{2}$ value with OLS is the variance explained by the first PC of 0.789, and analogously for score pairs 0.878 and for score triplets 0.926. The best score combinations are close to these upper bounds, in particular the score triplets, with $R_{\mathrm{all}}^{2}$ (10-fold) values being lower by only 0.128, 0.085, and 0.06 for individual scores, pairs, and triplets, respectively. We report the 10-fold cross-validation values for $R_{\mathrm{all}}^{2}$ here because it provides a more realistic performance estimate for unseen data compared to $R_{\mathrm{all}}^{2}$ (train). Taken together, the PCA revealed that most of the variance lies in a 2- or 3-dimensional subspace, and the analysis of optimal score combinations via OLS and $R_{\mathrm{all}}^{2}$ (10-fold) indicates that these 2- and 3-dimensional subspaces can be well approximated by two and three scores, respectively.

##### 3.1.4.2 Characteristics of High- and Low-Performing Score Combinations

One key finding presented in the previous paragraph is that many score combinations achieve comparable performances, indicating that although a particular score combination may be optimal according to the selected *R* ^2^ metric, several other combinations are likely to perform equally well in practical applications. That being said, some score combinations clearly perform better than others across all metrics, raising the question of what constitutes a high- and low-performing score combination. We will exemplarily investigate this question based on $R_{\mathrm{all}}^{2}$ (10-fold), as it is directly comparable across individual scores, pairs, and triplets and provides a more realistic performance estimate for unseen data compared to $R_{\mathrm{all}}^{2}$ (train). Nevertheless, the forthcoming findings are valid for all variants of *R* ^2^.

The three best and worst performing score combinations for individual scores, pairs, and triplets are shown in Table 2, with the complete lists provided in Supporting Information Table S2, S3, and S4, respectively. For individual scores, the best performing according to $R_{\mathrm{all}}^{2}$ (10-fold) are CAD (0.661), GDT (0.657), and SphereGrinder (0.655), whereas the worst are RMSD (0.545), QCS (0.537), and FlexE (0.401). This ranking is closely related to the pairwise score correlations presented in Figure 3a. Namely, the Pearson correlation between CAD and the remaining reference-based scores is, on average, highest at 0.80 (similarly, GDT = 0.80 and SphereGrinder = 0.79) and lowest for RMSD, QCS, and FlexE at 0.73, 0.68, and 0.59, respectively.

**Table 2:** Best- and Worst-Performing Score Combinations According to $R_{\mathrm{all}}^{2}$ and 10-Fold Cross-Validation (10-Fold)^a^

	Individual scores		Score pairs		Score triplets	
Rank	Scores	$R_{all}^{2}$ (10-fold)	Scores	$R_{all}^{2}$ (10-fold)	Scores	$R_{all}^{2}$ (10-fold)
1	CAD	0.661	GDT–FlexE	0.793	GDT–lDDT–QCS	0.866
2	GDT	0.657	TM-score–FlexE	0.791	RMSD–lDDT–QCS	0.859
3	SphereGrinder	0.655	RMSD–lDDT	0.786	GDT–CAD–QCS	0.855
⋮		⋮		⋮		⋮
n-3	RMSD	0.545	RMSD–TM-score	0.683	TM-score–GDT–QCS	0.754
n-2	QCS	0.537	RMSD–QCS	0.672	SphereGrinder–lDDT–CAD	0.738
n-1	FlexE	0.401	QCS–FlexE	0.613	RMSD–TM-score–GDT	0.732

a Metrics for all score combinations are given in Supporting Information Tables S2, S3, and S4.

For score pairs, the best-performing combinations are GDT–FlexE (0.793), TM-score–FlexE (0.791), and RMSD–lDDT (0.786), while the worst are RMSD–TM-score (0.683), RMSD–QCS (0.672), and QCS–FlexE (0.613). The scores FlexE, TM-score, and RMSD are therefore among both the best as well as the worst performing score pairs. This apparent contradiction largely reflects the results of the PCA analysis, specifically of the loadings plot shown in Figure 4b. Therein, most scores contributed similarly to the first PC, and the following three score clusters formed for the second PC: First, RMSD, GDT, TM-score, and QCS, second, SphereGrinder, lDDT, and CAD, and third, FlexE. Scores within one cluster contribute similarly to PC2 and thus contain similar information regarding this PC. For the best predictive performance of the OLS models, pairs for which each score contributes unique variance are apparently preferable. Therefore, the best-performing score pairs, even far beyond the top three, typically stem from different clusters, while the worst-performing stem from identical clusters. The exception is the lowest-ranked combination QCS–FlexE, where the low performance is potentially due to both scores having comparatively low correlations with all other scores.

For score triplets, the best-performing combinations are GDT–lDDT–QCS (0.866), RMSD–lDDT–QCS (0.859), and GDT–CAD–QCS (0.855), and the worst performing are TM-score–GDT–QCS (0.754), SphereGrinder–lDDT–CAD (0.738), and RMSD–TM-score–GDT (0.732). Notably, the difference between the best and the worst performing combination is only 0.127. Similar to score pairs, the ranking is explainable by the PCA loadings. Specifically, the 20 highest-ranked score triplets either stem from different clusters or contain two scores of the first PCA loadings cluster (RMSD, GDT, TM-score, and QCS), while the lowest-ranked typically contain scores from the same cluster.

To summarize, the best-performing individual scores tend to be those that are highly correlated with the remaining ones. For pairs and triplets, score combinations that contribute differently to PC2 in the PCA solution are preferrable, which is typically the case when combinations consist of one global score (or QCS), one local score, and FlexE (or, for pairs, scores from two of the three groups).

### 3.2 Do Force-Field Parameters Affect the Scores? Comparison of Four Common Simulation Setups

In this section, we investigate how sensitive scores are to the choice of the force field and its associated parameters. To this end, we first quantify the score variability arising from the stochastic nature of MD by conducting independent replica simulations with the same simulation setup C36m. This analysis, performed on a representative subset of 10 proteins spanning the full range of observed score values, provides a baseline for score reproducibility and quantifies score variability in the absence of any methodological change. Subsequently, we assess whether systematic score differences are present between the four commonly used simulation setups C36m, C36mPs3P, C36mPs4P, and A19sb.

#### 3.2.1 Estimation of MD Randomness

We selected 10 proteins from Set268 with diverse scores, performed five independent MD simulations each using the setup C36m, and scored the final simulation snapshots. To estimate the magnitude of score variability induced by MD randomness, we now quantify the within-protein variability and subsequently compare it to the between-protein variability. Here, within-protein refers to the variability between replica simulations of the same protein, while between-protein refers to the variability between different proteins based on the means over the corresponding replicas.

The scores of replica simulations are typically highly similar, as exemplarily shown for TM-score in Figure 6a and for all others in Supporting Information Figure S9. This similarity is directly reflected in low within-protein standard deviations, ranging, for example, from approximately 0.01 to 0.04 for TM-score (see Figure 6d; Supporting Information Figure S9 for all scores). Overall, this indicates that MD randomness has a limited impact on score variability with the following important exceptions: first, one replica of protein 4G3O has significantly lower scores than the other four replicas for TM-score, GDT, lDDT, CAD, and QCS. This is due to an α-helix rearrangement illustrated in Supporting Information Figure S10. Note that we excluded this replica from the calculations of the within-protein standard deviations because it represents a potential outlier that masks the general trend of the within-protein standard deviations increasing with lower-similarity scores. Second, for FlexE, proteins with high scores exhibit disproportionately large within-protein standard deviations (see Figure 6b and 6e). We attribute this effect to a small number of highly flexible and strongly deviating residues that dominate the score. For example, the 187 residue-long protein 5X88 perfectly aligns across all replicas except for two flexible loop regions (see Supporting Information Figure S11). Because FlexE scales quadratically with distance and lacks a cutoff, these large local deviations dominate the score and amplify variability across replicas. Indeed, a single residue in 5X88 contributes 6% to 13% to the final score depending on the replica, illustrating how local deviations can disproportionately influence the overall FlexE score. Third, MolProbity exhibits much larger within-protein standard deviations, with the replicas of the proteins 1UCS, 2J6Z, and 3LE4 spanning nearly the entire range of score values observed for all proteins in Set268 (see Figure 6c and 6f).

**Figure 6:**
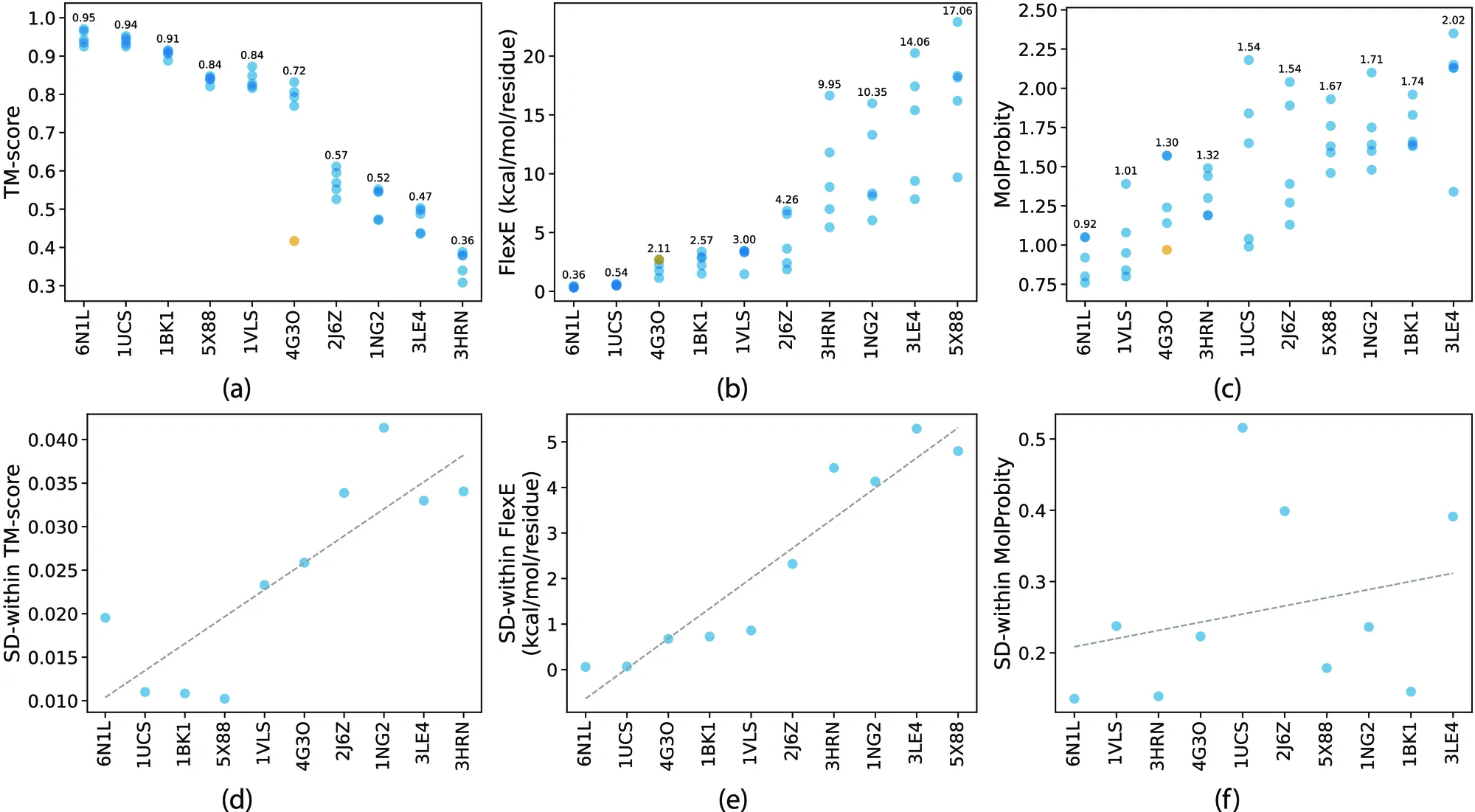
Scores and within-protein standard deviations of replica simulations for TM-score, FlexE, and MolProbity. TM-score is representative for most scores (see Supporting Information Figure S9), while FlexE and MolProbity are exceptions. (a)–(c) Score values for TM-score, FlexE, and MolProbity, respectively. For each protein, the replicas are displayed as blue spheres, with their corresponding mean value indicated above. Proteins are sorted from highest-similarity (highest-quality for MolProbity) to lowest-similarity based on the means for each score. One replica of protein 4G3O is a potential outlier and is highlighted in orange. (d)–(f) Within-protein standard deviations (SD-within) for TM-score, FlexE, and MolProbity, respectively. For 4GO3, the potential replica outlier was excluded from the calculation. The gray dashed line represents the OLS fit, illustrating that SD-within typically increases with lower-similarity scores.

Next, to compare the within-protein to the between-protein variability, we calculated their respective contributions to the total variability. The results are reported in Table 3, and computational details are given in Supporting Information Section S6. Across all scores, the within-protein variability accounts for less than 16% of the total variability, except for FlexE (19.1%) and MolProbity (39.2%). Taking the large score variations observed for high FlexE values into account, a within-protein contribution of 19.1% is still comparatively low. For MolProbity, however, almost 40% of the total variability arises from the randomness inherent to MD simulations.

**Table 3:** Decomposition of the Total Variability into Within-Protein and Between-Protein Variability Based on the Replica Simulations

Score	Within-protein variability (%)	Between-protein variability (%)
RMSD	1.8	98.2
TM-score	6.5	93.5
GDT	7.0	93.0
SphereGrinder	11.8	88.2
lDDT	15.7	84.3
CAD	12.5	87.5
FlexE	19.1	80.9
MolProbity	39.2	60.8

Taken together, scores are typically similar across replica simulations, and the within-protein variability is low compared to the between-protein variability. This indicates that scores are highly reproducible and protein-specific. Exceptions are the large within-protein variabilities observed for high FlexE values and MolProbity in general, making both scores less suitable for detecting differences between simulation setups.

#### 3.2.2 Comparison of Scores across Simulation Setups

We manually selected 31 proteins with diverse properties from Set268, simulated them with the four simulation setups C36m, C36mPs3P, C36mPs3P, and A19sb, and scored the final snapshots. Similar to Set268, we initially verified that scores are not biased by the properties of the studied proteins (analysis shown in Supporting Information Section S7). Here, we investigate whether systematic differences exist between the simulation setups. To this end, we first analyze score pair plots of simulation setups. Subsequently, we visually compare empirical score distributions across simulation setups for a qualitative assessment, quantify their absolute differences with the Earth Mover’s Distance (EMD),^56^ also known as the Wasserstein metric, and apply statistical tests to evaluate the significance of these differences.

##### 3.2.2.1 Score Pair Plots

We generated scatter plots for each score and combination of simulation setups. Figure 7 shows the results for GDT as a representative example, while plots for all scores are presented in Supporting Information Figures S15–S20. Visually, scores are highly similar and protein-specific across all simulation setups. This observation is supported by the corresponding Pearson correlation coefficients always exceeding 0.8, except those involving FlexE (*r*  > 0.69). Nevertheless, a slight but systematic shift toward higher-similarity scores is present for A19sb. This is clearly visible for GDT in Figure 7a–7c, where the majority of data points lie above the identity line.

**Figure 7:**
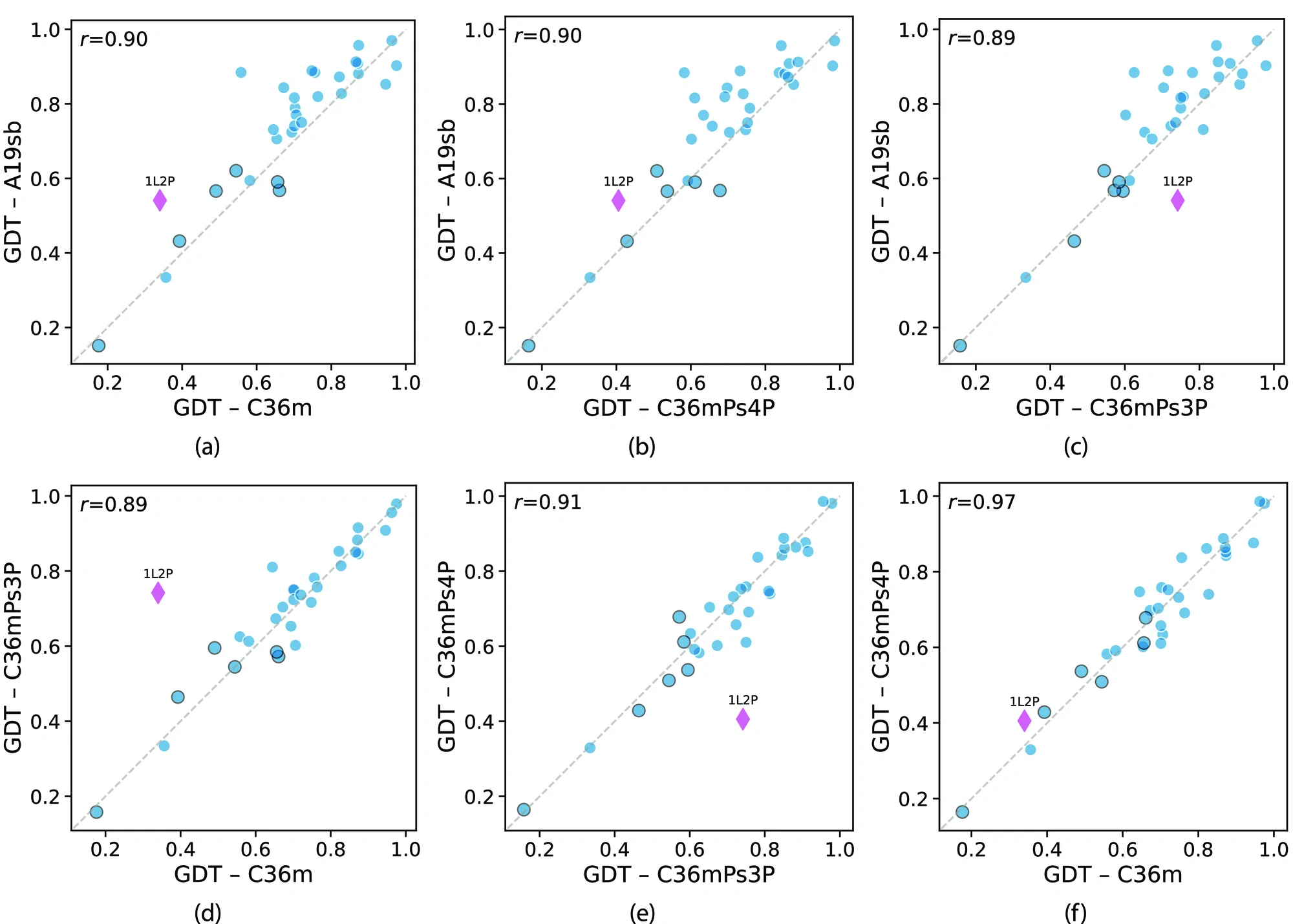
Score pair plots between the simulation setups C36m, C36mPs3P, C36mPs4P, and A19sb for GDT. Proteins are depicted as blue spheres with nonphysiological monomers marked by black circles. The potential outlier 1L2P is highlighted by purple diamonds. Pearson correlation coefficients are displayed in the upper left corners, and the gray dashed lines represent the identity lines, indicating perfectly consistent scores.

MolProbity and the protein 1L2P deviate from this otherwise consistent pattern. For MolProbity, the score variability across the simulation setups is considerably larger. Since this variability is symmetric and shows no systematic shift toward higher or lower scores for any specific simulation setup, it is most likely attributable to MD randomness from running multiple MD simulations rather than to genuine differences between the simulation setups. This interpretation is consistent with our previous analysis of MD randomness presented in Section 3.2.1.

The protein 1L2P yielded slightly and considerably higher-similarity scores for A19sb and C36mPs3P, respectively, than for C36m and C36mPs4P. 1L2P is a long α-helix of 61 residues and represents the dimerization domain of the ATP synthase b subunit, which is itself a component of the larger ATP synthase complex.^57^ It is therefore not surprising that 1L2P, as we simulated it in isolation, partially unfolded and disassembled into multiple smaller α-helices (see Supporting Information Figure S21) under all force field conditions used. Because this disassembly is highly stochastic, the observed score variability across simulation setups is likely attributable to this stochasticity rather than to systematic differences between force-field parameters.

##### 3.2.2.2 Qualitative Assessment of Score Distributions

The empirical score distributions of all simulation setups are shown for GDT, SphereGrinder, and MolProbity in Figure 8a and for every score in Supporting Information Figure S14. Visually, the distributions are highly similar in shape and range across all simulation setups despite being based on only 31 data points. Nevertheless, a systematic shift toward higher-similarity scores is present for A19sb, which is consistent with the findings of the pair plots. The observed shift becomes most evident when comparing medians across the simulation setups, as A19sb always yields the highest-similarity median for every score (see Supporting Information Table S5). MolProbity represents a partial exception. Although A19sb also yields the highest-similarity median for MolProbity, the medians across the simulation setups are virtually indistinguishable. Moreover, the MolProbity distributions have slightly different shapes and cover marginally different score ranges for each simulation setup.

**Figure 8:**
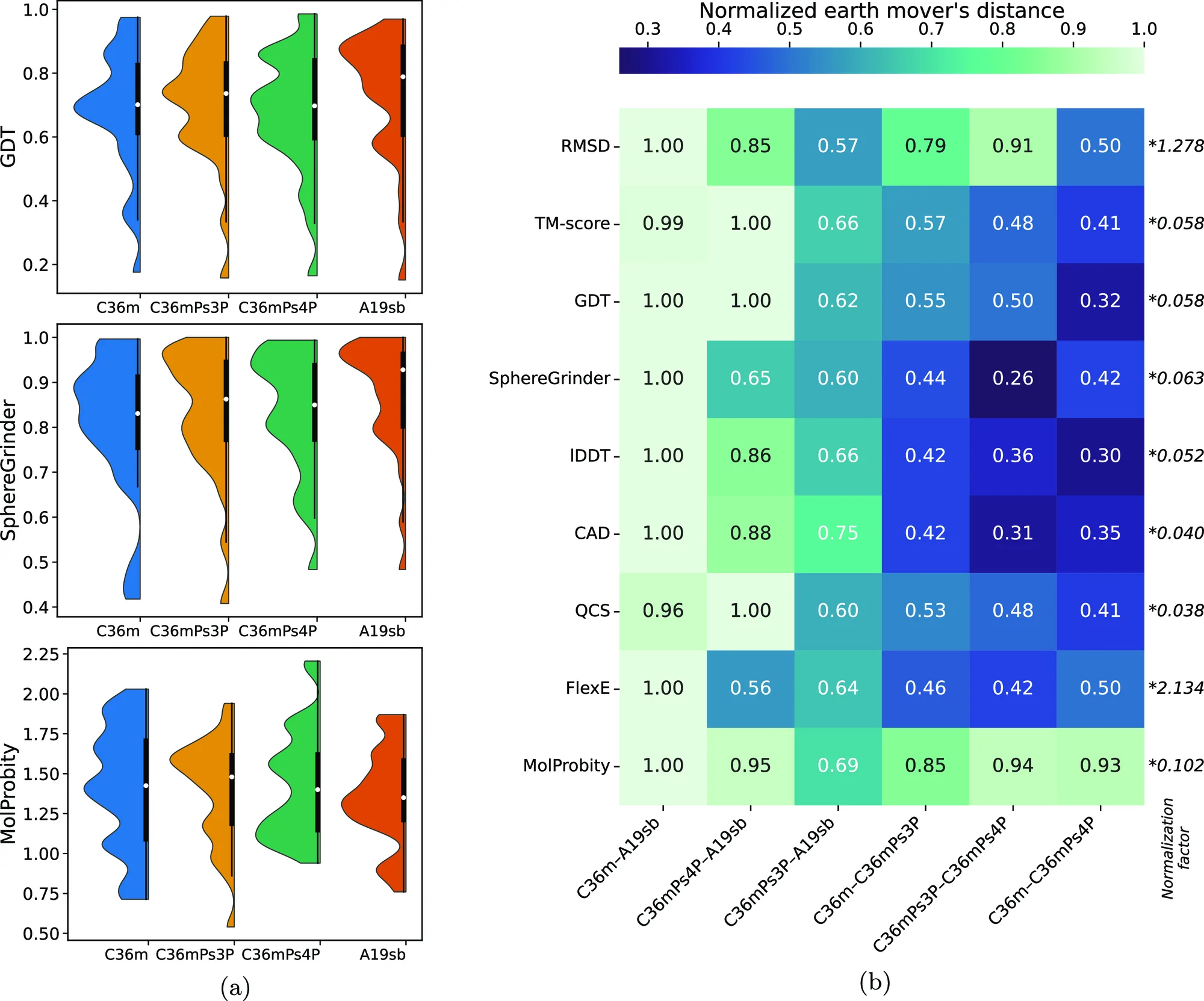
Comparison of simulation setups C36m, C36mPs3P, C36mPs4P, and A19sb. (a) The representative score distributions of GDT, SphereGrinder, and MolProbity displayed as violin plots for each simulation setup. Medians are depicted as white points, the ranges from the first quartiles to the third quartiles are marked by black boxes, and the lower and upper whiskers extend to the smallest and largest data points within 1.5 times the interquartile range from the first and third quartiles, respectively. (b) EMD for each score and pair of simulation setups. For each score (row), EMDs are normalized by the corresponding maximum value (normalization factor) to ensure consistent coloring. The pairs of simulation setups (columns) are sorted by the mean normalized EMD in descending order. Higher normalized EMDs are displayed in lighter colors and signify a larger discrepancy between the considered pair of simulation setups.

##### 3.2.2.3 Earth Mover’s Distance between Score Distributions

Intuitively, the EMD quantifies the cost of transforming one probability distribution into another.^56^ Since we are comparing one-dimensional score distributions based on identical proteins, the EMD can also be interpreted as the mean shift required to map one distribution onto the other. For each score, we calculated the EMD for all pairwise combinations of simulation setups to quantify the differences between their score distributions. We then normalized the resulting EMDs by the maximum observed EMD (normalization factor), enabling a straightforward comparison across scores. For seven out of nine scores, the normalized EMD between C36m and A19sb is the highest, while it is the second-highest for the remaining two scores (see Figure 8b), highlighting that scores from these two simulation setups differ the most overall. This trend is also reflected in the mean normalized EMD across all scores, which is highest for C36m and A19sb (0.99), followed by C36mPs4P and A19sb (0.86), C36mPs3P and A19sb (0.64), C36m and C36mPs3P (0.56), C36mPs3P and C36mPs4P (0.51), and C36m and C36mPs4P (0.46).

While the presented normalized EMDs facilitate comparisons across different scores, the corresponding normalization factors provide the quantitative scale of these differences. Specifically, the normalization factors are in the same order of magnitude as the within-protein standard deviations assessed in Section 3.2.1. Although they are not directly comparable, this nevertheless indicates that the differences between simulation setups are relatively small, consistent with the findings of the visual analysis (see Figure 8a).

##### 3.2.2.4 Friedman and Post-Hoc Wilcoxon Signed-Rank Test

Although the differences between the score distributions are comparatively small, they may still be meaningful. To examine this, we performed nonparametric statistical tests separately for each score, following a hierarchical testing strategy:^58^ first, we conducted the Friedman test^59^ to determine whether any statistically relevant differences exist among the simulation setups. If the Friedman test yielded a significant result, we carried out posthoc Wilcoxon signed-rank tests^60^ to identify which specific pairs of simulation setups differed significantly. Both tests were considered significant if the corresponding p-value, adjusted by the Benjamini–Hochberg correction,^61^ was ≤ 0.05. Note that the Friedman and Wilcoxon signed-rank test were selected instead of the more common repeated-measure ANOVA and paired *t* test because most score distributions are not normal (see Figures 2 and 8a).^58^

The Friedman tests are significant for every score except RMSD and MolProbity, and the follow-up Wilcoxon signed-rank tests are significant for C36m–A19sb and C36mPs4P–A19sb across all further-tested scores (p-values are listed in Supporting Information Table S6). For C36mPs3P–A19sb, it is significant for TM-score, SphereGrinder, lDDT, and CAD, but not for GDT, QCS, and FlexE. Differences between the simulation setups C36m, C36mPs3P, and C36mPs4P were not significant.

Taken together, our analyses indicate that for the final snapshots of 1 μs MD simulations A19sb, which uses the Amber ff19sb force field, yields significantly higher-similarity scores than the C36m, C36mPs3P, and C36mPs4P simulation setups, which employ the CHARMM36m force field. Thus, the conformations sampled after 1 μs by A19sb remain, on average, closer to the starting structure. Although the magnitude of these differences is relatively small, they are statistically significant. In contrast, varying the treatment of long-range vdW interactions and the water model for simulation setups based on the CHARMM36m force field did not have a statistically significant effect.

Thus far, our analysis has focused exclusively on score differences across simulation setups based on the final snapshots of the 1 μs MD simulations. Although a comprehensive trajectory-based analysis is beyond the scope of this study, we performed an illustrative trajectory analysis for GDT. The results are presented in Supporting Information Section S8 and are consistent with those of the final simulation snapshots, as normalized EMDs and the corresponding normalization factor were highly similar. Furthermore, A19sb yielded again higher-similarity scores than the CHARMM36m-based simulation setups.

## 4 Discussion and Conclusions

In this study, we quantified protein conformational changes in MD simulations using nine complementary scores and analyzed their similarities, as well as the extent to which they are influenced by the used force field and its parameters. Specifically, we addressed three main research questions, each of which is discussed below:

### 4.1 Are Score Correlations Equally High for Simulated Protein Structures as for Those Predicted by Machine Learning or Traditional Methods?

Our analyses reveal that scores are also highly correlated for protein structures originating from MD simulations, with Pearson and Spearman’s rank correlation coefficients consistently exceeding 0.7 (see Figure 3a and Supporting Information Figure S7). This aligns with the findings of Olechnovič et al., who reported, based on data from CASP10–12, slightly higher (approximately 0.05) Spearman’s rank correlation coefficients for a set of scores closely related to ours.^31^ The consistency between our findings for MD simulations and previous observations for predicted protein structures suggests that the scores, despite their methodological differences, characterize structural differences similarly across different types of structural data.

MolProbity is a notable exception, as it is uncorrelated with all other scores. This is unsurprising because MolProbity evaluates the quality of individual protein structures relative to idealized crystal structures rather than conformational differences between two structures. The lack of correlation between MolProbity and the other scores suggests that structural quality is largely independent of the extent of conformational changes during the simulation and that MolProbity captures information not reflected by the other metrics. However, MolProbity scores were consistently ≤ 2, indicating excellent structural quality, and exhibited substantial variability across replica simulations. We therefore argue that MolProbity is primarily useful as a rapid sanity check and during the development of new force fields, but provides limited additional value when evaluating simulations performed with established force fields.

### 4.2 Can One or a Few Scores Capture the Most Important Information Contained in All Studied Reference-Based Scores?

Our analyses consistently indicate that most of the variance is indeed shared and can be well approximated by just two or three scores. Specifically, the PCA results show that the first principal component explains 78.9% of the total variance, and the first two principal components collectively explain 87.8%. Moreover, using a small subset of scores to predict the remaining scores with an OLS model shows that two and three scores account for up to 79.3% and 86.6% of the total variance. Since an OLS model is unlikely to achieve the predictive performance of more flexible nonlinear models, these estimates are likely conservative. Nevertheless, two or three scores cannot capture all of the variance. Whether the remaining variance reflects noise or important information requires further investigation and may depend on the application or the protein under consideration.

Detailed assessment of the PCA loadings and the highest-performing score combinations reveals that one global score (or QCS), one local score, and FlexE together capture the maximum information contained across all scores. However, we observed considerable score variability at high FlexE values, likely because FlexE overpenalizes structural differences in highly flexible protein regions. This behavior arises from the absence of a distance cutoff and the quadratic scaling of the score with distance, allowing individual residues to dominate the overall score. Consequently, FlexE may reasonably be excluded for applications in which such local structural variations are not of primary interest.

### 4.3 How Much Does the Choice of Force Field and Its Parameters Affect Protein Conformations After MD, and Thus the Scores?

Our comparison of score distributions reveals small differences in the magnitude of variability typically introduced by MD randomness between the simulation setups C36m, C36mPs3P, C36mPs4P, and A19sb. Nevertheless, statistical tests consistently identify significant differences between A19sb and the CHARMM36m-based setups, as A19sb yielded systematically higher-similarity scores after 1 μs MD simulations. This indicates that force-field choices can influence structural outcomes of MD to a measurable extent. Since A19sb differs from the CHARMM36m-based setups in multiple aspects, including the force field, water model, and the treatment of long-range vdW interactions, it is challenging to attribute these differences to a single factor. The absence of significant differences between the CHARMM36m variants suggests that the force field itself might be the dominant factor.

A more comprehensive analysis with additional simulation setups and potentially also larger data sets would be required to draw definitive conclusions. Thus, this study provides an initial systematic exploration rather than an exhaustive benchmark.

### 4.4 Future Work

In this study, we focused on the final snapshots of MD simulations. A logical next step is to analyze protein dynamics over the course of MD simulations and investigate whether scores can detect interesting, sudden, and temporal conformational changes relative to the starting structures or even the previous simulation snapshots. In this context, it will be interesting to examine whether all scores assess these changes equally well.

## 5 Data and Software Availability

The following data are available in the repository https: 10.18419/DARUS-5853:

- Final simulation snapshots and corresponding starting structures of all proteins (PDB files)
- Score values of the final simulation snapshots for RMSD, TM-score, GDT, SphereGrinder, lDDT, CAD, QCS, FlexE, and MolProbity
- Jupyter Notebook files containing all analyses conducted in this study
- Python scripts of FlexE and SphereGrinder implementations. Modified python script *getQCS.py*
- Force field and mdp files

## Supplementary Material







## Notes

PDB IDs, properties, and assignment to Set268 and Set31 for the studied proteins (CSV)
Section S1: definitions of utilized scores; Sections S2–S3 and Table S1: description of protein structure acquisition and simulation, including details on simulation parameters; Figure S1: histograms of properties for the studied proteins; Figure S2: quantile–quantile plot for MolProbity; Figure S3: histograms of MolProbity subscores for crystal structures and final simulation snapshots; Section S4 and Figure S4: evaluation of Pearson correlation between protein properties and scores; Figure S5: pair plots between protein properties and scores; Figure S6: pair plots between scores; Figure S7: Spearman’s rank correlation matrix of scores; Figure S8: PCA loadings plot including MolProbity; Section S5: column subset selection problem (CSSP) and proofs for interpreting *R* ^2^ as a fraction of total variance; Tables S2–S4: *R* ^2^ performance of all score combinations (CSSP); Figure S9: scores for replica simulations and the corresponding within-protein standard deviation; Figures S10–S11: final snapshots of 4G3O and 5X88 across replica simulations; Section S6: variance decomposition of replica simulations; Section S7 and Figures S12–S13: assessment of score-property Pearson correlation for the simulation setups C36m, C36mPs3P, C36mPs4P, and A19sb; Figure S14 and Table S5: score distributions for the simulation setups C36m, C36mPs3P, C36mPs4P, and A19sb, and the corresponding ranking of medians; Table S6: p-values for Friedman and Wilcoxon signed-rank tests to assess differences in score distributions across simulation setups; Figures S15–S21: score pair plots for each combination of simulation setups; Figure S21: final snapshots of 1L2P across replica simulations; Section S8, Table S7, and Figure S22: comparison of the simulation setups C36m, C36mPs3P, C36mPs4P, and A19sb based on entire simulation trajectories and GDT (PDF)
Alignment to UniProt sequences for the studied proteins (CSV)
