# Supplementary material for: Which Metrics Best Capture Protein Structural Changes in Molecular Dynamics Simulations? Evaluating Score Combinations and Force-Field Effects
Source: J Chem Inf Model. 2026 Aug 26;66(17):11211–25. doi: 10.1021/acs.jcim.6c01218 (PMC13580131; doi:10.1021/acs.jcim.6c01218)
Supplement: Supplementary file 1 [file ci6c01218_si_002.pdf]

Supporting Information: Which Metrics Best Capture Protein Structural Change in Molecular Dynamics Simulations? Evaluating Score Combinations and Force Field Effects

Christian Pfaendner[1,2], Thilo Paul[1], Benjamin Unger[3], and Kristyna Pluhackova*[1]

[1]Stuttgart Center for Simulation Science, Cluster of Excellence EXC 2075, University of Stuttgart, Universitätsstr. 32, 70569, Stuttgart, Germany
[2]Artificial Intelligence Software Academy, University of Stuttgart, 70569, Stuttgart, Germany
[3]Institute for Applied and Numerical Mathematics, Karlsruhe Institute of Technology, Englerstr. 2, 76131 Karlsruhe, Germany

*Email: kristyna.pluhackova@simtech.uni-stuttgart.de

S1 Definitions of Protein Scores

Protein scores either directly assess the quality of a query structure or compare it to a reference structure. In the field of structure prediction, the query structure is predicted, for example, via AlphaFold [1], and the reference structure is obtained from experimental methods. In our case, the query structure is the final snapshot of a molecular dynamics (MD) simulation, and the reference structure is the corresponding starting snapshot, which represents the crystal structure obtained from the RCSB database [2]. In the following, we define and describe the scores used in this study, assuming that the reference and the query structure contain the same set of atoms.

S1.1 RMSD

The root mean square deviation (RMSD) [3] quantifies how well the query structure can be superimposed onto the reference structure by minimizing the objective function

$$\text{RMSD} = \sqrt{\frac{1}{N}\sum_{i=1}^{N} d_i^2}, \tag{E1}$$

where N is the total number of atoms, and d_i is the Euclidean distance between atom i of the query and the reference structure. RMSD can be calculated for the entire structure or for a specific subset of atoms. In this study, RMSD is based on the Cα atoms and given in Å. RMSD is always zero or positive: $\text{RMSD} \in \mathbb{R}_0^+$, with lower values indicating better agreement between the query and the reference structure. RMSD was calculated together with TM-score and GDT with code downloaded from Zhang Lab: `https://zhanggroup.org/TM-score/`.

S1.2 TM-score

The template modelling score (TM-score) [4] assesses how accurately the query structure can be superimposed onto the reference structure. Unlike RMSD, which minimizes Equation E1, the TM-score maximizes

$$\text{TM-score} = \frac{1}{N}\sum_{i=1}^{N} \frac{1}{1 + (\frac{d_i}{d_o})^2}, \tag{E2}$$

where N is the total number of Cα atoms, d_i is the Euclidean distance between Cα atom i of the query and the reference structure, and $d_0 = 1.24\sqrt[3]{N-15} - 1.8$ is a normalization factor to mitigate the effect of different protein lengths. TM-score considers only Cα atoms and ranges from zero to one: $\text{TM-score} \in [0, 1]$, with higher values indicating better agreement between the query and the reference structure. TM-score was calculated together with RMSD and GDT using code downloadable from Zhang Lab: `https://zhanggroup.org/TM-score/`.

S1.3 GDT

The global distance test (GDT) [5, 6] superimposes the query onto the reference structure multiple times to evaluate their similarity. Each superposition is performed such that the fraction F_d of atoms in the query structure that lie within the Euclidean cutoff distance d of their corresponding atoms in the reference structure is maximized. The GDT total score (GDT-TS), using the cutoffs 1, 2, 4, and 8 Å, is then given by

$$\text{GDT-TS} = \frac{1}{4}(F_1 + F_2 + F_4 + F_8). \tag{E3}$$

Another variant is the GDT high accuracy score (GDT-HA), which uses the cutoff distances 0.5, 1, 2, and 4 Å. Both GDT variants focus on well-modeled residues and are therefore more robust against outliers than, for example, RMSD. Throughout this study, the GDT-TS score is exclusively used and referred to as GDT for simplicity. GDT considers only Cα atoms and ranges from zero to one: $\text{GDT} \in [0, 1]$, with higher values indicating better agreement between the query and the reference structure. GDT was calculated together with RMSD and TM-score with code downloaded from Zhang Lab: `https://zhanggroup.org/TM-score/`.

S1.4 lDDT

The local distance difference test (lDDT) [7] quantifies how well interatomic distances in the query structure are preserved relative to the reference structure, without requiring structural superposition. Specifically, it evaluates all pairs of atoms $p \in P$ in the reference structure whose atoms belong to different residues and are separated by a distance of at most 15 Å (by default). For each pair p, the absolute difference between the interatomic distances in the query and the reference structure is calculated as $\Delta d_p = |d_p^{ref} - d_p^{mod}|$. Subsequently, the number of pairs M_c for which $\Delta d_p < c$ is counted for a set of distance thresholds $c \in 0.5, 1, 2, 4$ Å. lDDT is then defined as

$$\text{lDDT} = \frac{1}{4}\frac{M_{0.5} + M_1 + M_2 + M_4}{|P|}, \tag{E4}$$

where P is the total number of atom pairs. lDDT considers all heavy atoms and ranges from 0 to 1: lDDT $\in [0, 1]$, with higher values indicating better agreement between the query and the reference structure. lDDT was computed with OpenStructure [8]: `https://openstructure.org/`.

S1.5 SphereGrinder

SphereGrinder [9] assesses how similar local environments are between the query and the reference structure. For each central atom (Cα by default) in the reference structure, all neighboring atoms within a specified distance d are determined. In our work, we set $d = 6$ Å, following the choice made in a study by Olechnovič *et al.* [10] and CASP10 [11]. Subsequently, the corresponding neighboring atoms in the query structure are used to calculate RMSD for each central atom's environment. The final score is then given by

$$\text{SphereGrinder} = \frac{M_2 + M_4}{2N}, \tag{E5}$$

where N is the total number of central atoms, and M_2 and M_4 denote the number of central atoms whose local environments were superimposed with an RMSD smaller than 2 Å and 4 Å, respectively. This definition was also used in the study by Olechnovič *et al.* [10], whereas SphereGrinder was defined as M_2/N in CASP10 [11]. Here, Cα atoms are designated as the central atoms, while all heavy atoms are considered neighboring atoms. SphereGrinder ranges from 0 to 1: SphereGrinder $\in [0, 1]$, with higher values indicating better agreement between the query and the reference structure. SphereGrinder was calculated with an in-house implementation.

S1.6 QCS

The Quality Control Score (QCS) [12] was originally developed to approximate the outcomes of query structure inspection and places a strong emphasis on secondary structure elements (SSEs). SSEs in the reference structure are first identified using PALSSE [13]. Each SSE is then linearized by constructing a vector from the first to the last Cα atom. It is assumed that the residues forming an SSE in the reference structure form the same SSE in the query. Consequently, the corresponding SSE vectors are constructed in the query without reapplying PALSSE. QCS is calculated as the weighted average of individual subscores, each quantifying a different structural aspect: length, global position, angles, handedness and interaction of SSEs, and Cα–Cα contact similarity. By default, each subscore contributes equally to QCS. The exact calculation of the subscores can be found in the original study by Cong *et al.* [12]. Note that the angle and interaction subscores are only defined for proteins containing at least two SSEs, while the handedness subscore requires at least three SSEs. In cases where these conditions are not met, the respective subscores are set to zero, which can artificially lower the final value of QCS. To avoid this bias, we consider QCS to be undefined for proteins with fewer than three SSEs. QCS ranges from 0 to 1: QCS $\in [0, 1]$, with higher values indicating better agreement between the query and the reference. QCS was calculated with code downloaded from the website `http://prodata.swmed.edu/QCS/`. Notably, this QCS implementation does not support parallel comparisons because all temporary files are written to a single directory. Consequently, analyses of multiple proteins must be carried out sequentially. We therefore modified the script `getQCS.py` so that temporary files are appended by a unique identifier.

S1.7 CAD

The contact area difference (CAD) score [14] quantifies the agreement between residue–residue contact areas in the query and the reference structure. It is defined as

$$\mathrm{CAD} = 1 - \frac{\sum_{p \in P} \min(|C_p^{\mathrm{ref}} - C_p^{\mathrm{que}}|, C_p^{\mathrm{ref}})}{\sum_{p \in P} C_p^{\mathrm{ref}}}, \tag{E6}$$

where P is the set of all contacting residues in the reference structure, C_p denotes the contact area between the residues forming pair $p \in P$, and ref and que indicate the reference and query structure, respectively. The contact area C_p is calculated at the atomic level via the following steps:

1. Determine the Voronoi diagram for all atoms in the structure
2. For each atom, construct a sphere whose radius is the sum of the atom's van der Waals (vdW) radius and the radius of a water molecule (1.4 Å)
3. A point x on the contact sphere of atom i with respect to atom j contributes to their contact surface area if:
   - i and j are Voronoi neighbors, and
   - x is closer to j than to any other neighbor of i

The contact area between two residues is then calculated as the sum of the contact areas of all their constituent atoms. By default, all heavy atoms are included in the calculation, although custom atom selections can be specified. In this study, we keep the default settings. CAD ranges from 0 to 1: $\mathrm{CAD} \in [0, 1]$, with higher values indicating better agreement between the query and the reference structure. CAD was calculated using the Voronota package [15], which is available on GitHub: `https://github.com/kliment-olechnovic/voronota`.

S1.8 FlexE

FlexE [16] is based on elastic network models and estimates the energy required to deform the reference into the query structure. In the reference structure, Cα atoms are connected by springs if they are at most third-degree neighbors along the backbone. Additionally, any Cα atoms that are spatially close (within 8 Å) are connected by springs whose force constants depend on the interatomic distance - stronger for atoms that are closer. The deformation energy is then computed as the energy required to morph the reference network into the query configuration. FlexE is given in kcal/mol/residue and is always zero or positive: $\mathrm{FlexE} \in \mathbb{R}_0^+$, with lower values indicating better agreement between the query and the reference structure. FlexE was calculated with an in-house implementation. Our code is a modified version of the original implementation (`https://github.com/laufercenter/FlexE`) referenced in the work by Perez`et al.` [16].

S1.9 MolProbity

MolProbity [17, 18] assesses the quality of a single protein structure, without requiring a reference structure for comparison. To this end, it evaluates interatomic clashes, bonds, angles, and dihedral angles through multiple subscores by comparing them to their expected values and distributions. Specifically, these subscores include, among others:

- Clashscore: Number of overlaps per 1000 atoms. Two atoms are clashing if their vdW radii overlap by at least 0.4 Å.
- RMSZ-bonds and RMSZ-angles: Deviation of bond lengths and bond angles, respectively, from expected ideal geometries. For each bond or angle, a Z-score is computed by subtracting the expected mean value and dividing by the standard deviation of the expected distribution. The final RMSZ value is then obtained by calculating the root mean square (RMS) of all individual Z-scores.

- Percentage of favored and disfavored rotamer/Ramachandran angles: Rotamer and Ramachandran reference distributions are defined by contouring empirical data derived from high-resolution crystal structures. Individual values are classified as favored if they fall within the contour and as disfavored if they lie outside. For rotamers, approximately 1 % of the reference data falls outside the defined contour, while for Ramachandran angles, this proportion is even smaller—less than 0.1 %.

- Rama-Z: Also evaluates Ramachandran backbone angles, but unlike hard thresholds based on contouring, it assesses the overall distribution of backbone angles in the query structure compared to an expected reference distribution. Expected is a Rama-Z score of 0, while values exceeding ± 3 suggest potential structural anomalies or systematic deviations. Details on the calculation of the Rama-Z score can be found in the original study by Hooft *et al.* [19].

MolProbity is calculated as a log-weighted sum of the Clashscore, the percentage of unfavorable Ramachandran angles, and the percentage of unfavorable rotamers. Intuitively, it corresponds to the crystallographic resolution (in Å) at which a structure with comparable geometric quality would typically be observed. MolProbity is always zero or positive: MolProbity $\in \mathbb{R}_0^+$, with lower values indicating a higher agreement with expected values. The code for determining the MolProbity score, which was used in this study, is available on GitHub: `https://github.com/rlabduke/MolProbity`.

S2 Protein Structure Acquisition of Set268 and Set31

Initially, the RCSB advanced search query builder [2] was used to select 3196 crystal structures, which only contain a single polymer entity (chain), consist of 50 to 250 residues per chain, and were resolved at 2 Å or less. As a result, the candidate structures are either monomers or multimers consisting of copies of the same chain. Subsequently, they were filtered with the RCSB API [2] to include only proteins without missing residues within the sequence and with exactly one corresponding UniProt ID [20] with a single aligned region. The candidate with the lowest resolution was chosen if multiple candidates mapped to the same UniProt ID. The PDB files for the resulting 1467 candidate structures were downloaded, and in the final filtering step, any structure containing molecules other than protein and water was discarded. The 1156 filtered candidate structures were then analyzed using DSSP [21, 22] to determine their secondary structure composition. From these, 207 final structures were selected based on the following criteria: 50 structures with the highest α-helix content; 10 with the highest 3_{10}-helix content; 10 with the highest π-helix content; 50 with the highest β-sheet content; 50 with the highest unstructured content (sum of turn, bend, and loop contents); 30 with α-helix content between 0.3 and 0.7; 10 with β-sheet content between 0.45 and 0.55; 10 with unstructured content between 0.45 and 0.55. If more structures met a criterion than the target number, a random selection was made. Note that a single protein could satisfy multiple criteria, resulting in overlap and a discrepancy between the theoretical maximum of 220 selections and the 208 actual unique structures. Additionally, one protein was removed because score computation failed in downstream analyses, resulting in a final set of 207 protein structures. Their properties, that is, the number of residues per chain, the resolution at which the structures were resolved, the number of disulfide bonds, and the secondary structure composition, are visualized as histograms in Figure S1.

We also generated the corresponding oligomeric structures from each PDB file whenever a higher-order assembly was indicated by the ‘author determined biological unit’ or the ‘software determined quaternary structure’ field in remark 350. To this end, we applied the given transformation matrix. In cases where the author and software disagreed on the oligomeric state (e.g., dimer vs. tetramer), we generated both versions. This process resulted in 62 additional oligomeric structures: 36 dimers, 14 trimers, seven tetramers, two hexamers, two octamers, and one 24-mer, whereby the latter was excluded due to its large size. The monomers and oligomers together form the complete set of 268 proteins, called Set268.

From Set268, 31 proteins were manually selected to represent a wide range of protein properties (see Figure S1). This subset is denoted as Set31 and consists of 20 monomers (14 physiological and six non-physiological), six dimers, three trimers, one tetramer, and one octamer. The corresponding PDB IDs are provided in the supplementary file *set268_set31_properties.csv*.

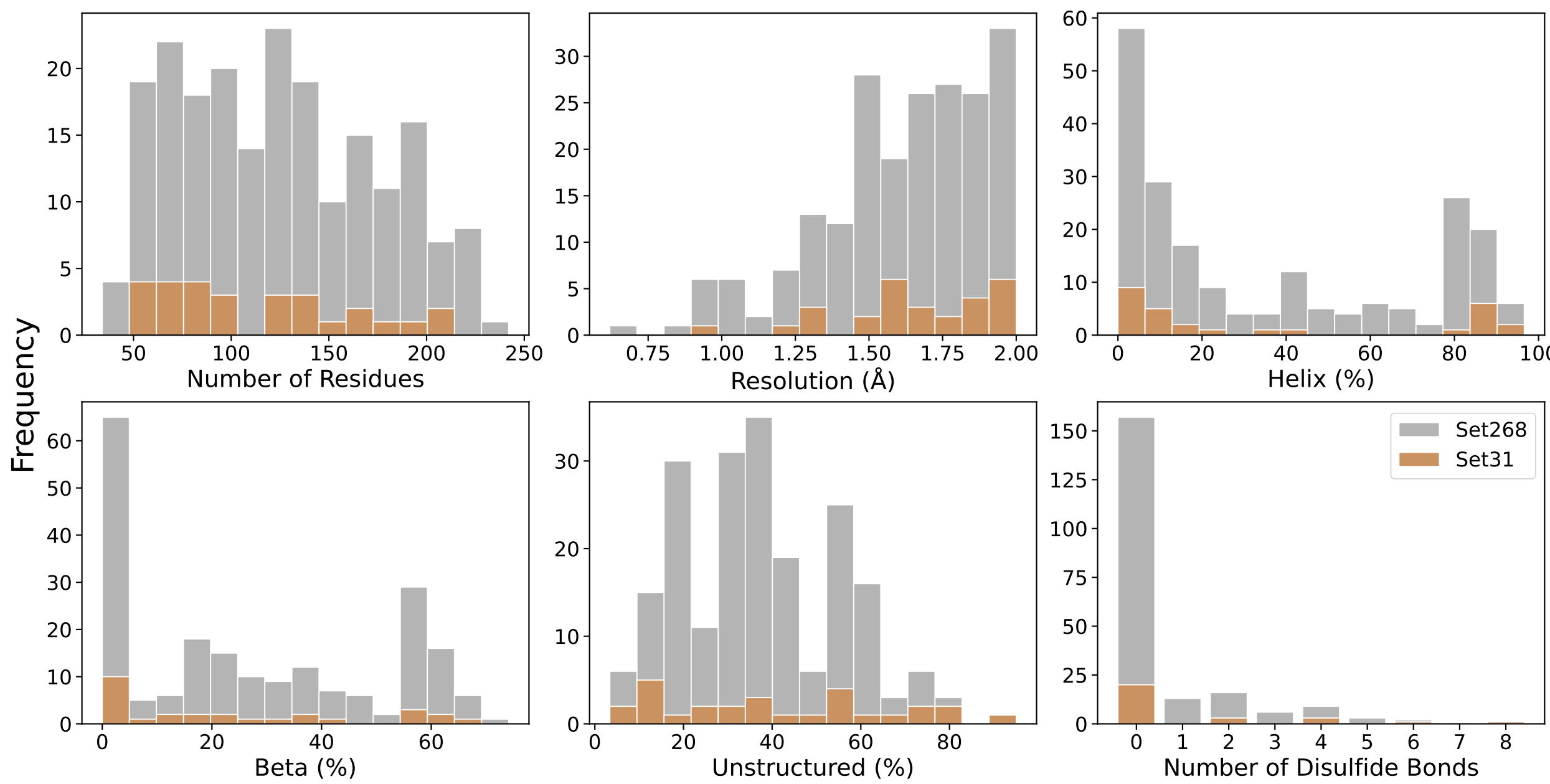

Figure S1: Property distributions of investigated proteins in Set268 and Set31. Resolution refers to the level of detail at which each protein structure was experimentally resolved. Helix is the percentage of residues in each protein classified as α-helix, 3_{10} helix, π-helix, or PPII helix by DSSP [21, 22]. Beta is the percentage of residues in each protein classified as β-bridge or β-sheet by DSSP. Unstructured is the percentage of residues in each protein classified as turn, bend, or loop by DSSP. Oligomers were always derived from their monomers, and their properties consequently differ only in the number of residues. Including both would duplicate information and therefore distort the distributions. Hence, for Set268 only the properties of the contained 207 monomers are shown. For Set31, if both the monomer and its corresponding oligomer are in the set, only the monomeric properties are included in the distributions. If only the oligomer is present, the properties of one of its monomers are used for consistency. Due to this strategy, only the properties of 28 proteins are effectively shown for Set31.

S3 Molecular Dynamic Simulations

All simulations were run with GROMACS version 2023 [23, 24].

S3.1 Preparation and Simulation Protocol

The following sequential steps were conducted for all proteins: First, we extracted the protein from each PDB file and removed crystallographic water molecules. We note that while retaining specific crystallographic waters can be advantageous for studies on conserved hydration sites, active-site interactions, or ligand binding [25], where such waters may play a structural or functional role, our study focuses on the systematic comparison of scores that quantify conformational differences, rather than hydration effects. In the next step, we added missing heavy atoms with the program Modeller [26] and hydrogen atoms via the GROMACS built-in command `gmx pdb2gmx`. All titratable residues were assigned their standard protonation state at pH 7. Next, we compared the N- and C-terminal sequences with their corresponding full-length sequences from UniProt to assign the appropriate protonation states to the terminal residues. If the terminal residue matched the respective terminus of the full-length UniProt sequence, we assumed the protein was complete at that terminus and applied standard protonation: a positively charged amine group for the N-terminus and a negatively charged carboxylate for the C-terminus. Conversely, if the terminus did not represent the true start or end of the full-length sequence, suggesting the protein was truncated or incomplete, we treated the respective terminal group as neutral. In simulations using the Amber ff19sb force field, the neutral termini were capped, as the Amber force field doesn't include parameters for simply neutralized termini. Specifically, the N-terminus was capped with an acetyl group (replacing the hydrogen atom bonded to the nitrogen), while the C-terminus was capped with an amino group (replacing the single-bonded oxygen). Subsequently, a dodecahedron simulation box was added with a distance of 1.5 nm to the protein using GROMACS `gmx editconf`. Afterwards, a single energy minimization (EM) of 100 steps was performed in vacuum. The system was then solvated in water with GROMACS `gmx solvate`, and random water molecules were replaced by chloride and sodium ions to obtain concentrations of $0.15\,\mathrm{mol\,L^{-1}}$ using GROMACS `gmx genion`. This was followed by three successive EMs. The first EM comprised 500 steps with the entire protein frozen in all directions, the second consisted of 1000 steps with only the backbone frozen, and a final 1000-step minimization with all restraints removed. Next, two 1 ns position-restraint simulations were conducted to equilibrate the system. In the first simulation, all heavy atoms of the protein were position restrained, whereas for the second simulation, only the heavy atoms of the backbone were constrained. For both position-restraint simulations a force constant of $1000\,\mathrm{kJmol^{-1}nm^{-2}}$ was used. Finally, a production 1 µs MD simulation was carried out. Detailed simulation parameters beyond this general description can be found in the next Section S3.2.

S3.2 Simulation Parameters

S3.2.1 CHARMM36m

For EMs, the steepest descent algorithm was used with a force tolerance of $10\,\mathrm{kJ\,mol^{-1}\,nm^{-1}}$ and an initial step size of 0.01 nm. Additionally, the CHARMM TIP3P (or TIP4P - see Section S3.1) water was treated as flexible. Position-restraint and production simulations were carried out by the leap-frog algorithm with a step size of 2 fs, and the LINCS algorithm[27] constrained the bonds to the hydrogen atoms. The Verlet cutoff scheme [28] was used for neighbor list generation with a buffer tolerance of $0.005\,\mathrm{kJ\,mol^{-1}\,ps^{-1}}$ per atom and an update frequency of 10 steps. The vdW interactions were calculated with a cutoff of 1.2 nm and force-switched (or potential-switched - see Section S3.1) to zero between 0.8 and 1.2 nm. Coulomb interactions were computed directly up to 1.2 nm, with the potential-shift modifier applied from 0 nm to 1.2 nm. Long-range electrostatics beyond 1.2 nm were treated using the particle mesh Ewald (PME) method [29]. Periodic boundary conditions were applied throughout and in all simulations the compressibility amounted to $4.5 \times 10^{-5}\ \mathrm{bar^{-1}}$.

Specifics for EM in Vacuum: The EM in vacuum was performed without PME.

Specifics for Position Restraint Equilibrations: The Berendsen thermostat [30] coupled the systems to 310 K every 0.5 ps separately for protein and solvent and the Berendsen barostat [30] coupled the systems to 1 bar every 5 ps.

Specifics for Production Run MD Simulation: The linear center-of-mass motion was removed every 500 steps. The Nosé-Hoover thermostat [31] was used for temperature coupling to 310 K every 0.5 ps (separately for protein and solvent) and the c-rescale barostat [32] for pressure coupling to 1 bar every 5 ps. Coordinates of the protein and the ions were written out every 100 ps for each system.

S3.2.2 Amber ff19sb

EMs used the steepest descent algorithm with a force tolerance of 10 kJ mol^{-1} nm^{-1} and an initial step size of 0.01 nm. OPC water molecules were treated as flexible. Position-restraint and production simulations were performed with a time step of 2 fs, compressibility of 4.5×10^{-5} bar^{-1} and the LINCS algorithm [27] constrained the bonds to the hydrogen atoms. The Verlet cutoff scheme [28] was used for neighbor list generation, with a buffer tolerance of 0.005 kJ mol^{-1} ps^{-1} per atom and an update frequency of 10 steps. The vdW interactions were computed up to a cutoff of 1.0 nm and the potential-shift modifier was used. Additionally, a dispersion correction [33] of energy and pressure was applied for long-range vdW interactions. Coulomb interactions were calculated directly up to 1.0 nm, with a potential-shift modifier applied over the 0 nm to 1.0 nm interval. Long-range electrostatics beyond this range were handled using the PME method [29]. Periodic boundary conditions were applied in all simulations. To implement amino-acid-specific CMAPs in GROMACS versions prior to 2025, we have replaced the XC atom type of Cα atoms by XC0 till XC15, which are assigned exactly the same interactions (both bonded and non-bonded) as the original XC atom type, yet carry different CMAPs (see Table S1).

Table S1: Atom types for Cα atoms of groups of amino acids allowing assignment of diverse CMAP potentials. The bonded and nonbonded parameters for atom types XC0-XC15 are equal to the original XC type. The same CMAP is used for all different protonation states of each amino acid marked with a star. In detail, the different protonation states are CYS, CYX, and CYM for CYS; HID, HIE, and HIP for HIS; LYS, and LSN for LYS.

Atom type	Amino Acids
XC0	ALA
XC1	SER
XC2	GLU
XC3	HIS*, MET, LEU, TRP, PHE, TYR
XC4	LYS*
XC5	ARG
XC6	ILE, VAL
XC7	GLY
XC8	PRO
XC9	ASN
XC10	GLN
XC11	THR
XC12	ASP
XC13	CYS*
XC14	ASH
XC15	GLH

Specifics for EM in Vacuum: The EM in vacuum was performed without PME and dispersion correction.

Specifics for Position Restraint Equilibrations: The systems were coupled to the reference temperature of 310 K every 0.5 ps separately for protein and solvent with the Berendsen thermostat [30] and to a reference

pressure of 1 bar every 5 ps with the Berendsen barostat [30]. The modified termini of the proteins were not restrained.

Specifics for Production Run MD Simulation: The linear center-of-mass motion was removed every 500 steps. The Nosé-Hoover thermostat [31] coupled the systems to 310 K every 0.5 ps (separately for protein and ions) and the c-rescale barostat [32] coupled the systems to 1 bar every 5 ps. Coordinates of proteins and ions were written out every 100 ps.

S3.3 Background on Utilized Force Fields, vdW Modifiers, and Water Models

S3.3.1 Force Fields

We simulated proteins either using the CHARMM36m or the Amber ff19sb force field. In general, CHARMM36 [34–36], which was extended in version CHARMM36m by backbone parameters for disordered proteins [37], is a widely used biomolecular force-field [38]. It is particularly valued for the MD simulation of membrane proteins [39–41], due to its extensive set of force-field parameters for hundreds of lipids and sterols, as well as the ease of system preparation via CHARMM-GUI [42]. Amber is also a commonly used force field, particularly for studying the structure and dynamics of biomolecules like proteins and DNA in solution [43, 44]. The current version of the Amber force field for proteins is Amber ff19sb [45], which extends the successful Amber ff14sb version [46] by specific amino-acid backbone parameters. This refinement improves helical propensities and more accurately reproduces amino-acid–specific Ramachandran plots.

S3.3.2 vdW Modifiers

For the vdW modifiers, GROMACS provides the options force switching, which is the default option and switches the forces to zero as the interaction distance approaches the defined cutoff, potential switching, which smoothly reduces the potential instead of the forces to zero in the defined range, and potential shifting, which subtracts a constant so that the potential is zero at the cutoff. Each method has its tradeoffs: Force switching ensures continuous potentials and forces at a moderate computational overhead. Potential switching also provides continuity of potentials and forces, and, additionally, explicitly alters the potentials compared to force switching. However, the computational cost is higher, and artificially large forces are introduced in the switching region. In contrast, potential shifting preserves the shape of the potentials with negligible overhead, though it alters the entire potentials even before the cutoff. Moreover, it introduces a discontinuity of the potentials at the cutoff [47, 48]. For CHARMM36m, the forces or potentials were switched to zero between 8 and 12 Å, which is the recommended setting of the original CHARMM-GUI input generator publication [42] and is the common setting for lipid membrane simulations [49, 50]. For the A19sb parameter set, the Amber-specific cutoff for potential shifting was 10 Å, a common setting in GROMACS.

S3.3.3 Water Models

We performed CHARMM36m simulations either with the CHARMM TIP3P or the TIP4P water model, while the Amber ff19sb simulations utilized the OPC water model. The CHARMM TIP3P water model, which includes Lennard–Jones parameters also for hydrogens [51], was utilized during parametrization of the CHARMM force fields [35–37, 52] and is therefore recommended for simulations with CHARMM force fields. Another contributing factor to its wide usage is the approximately 7 % faster runtime compared to TIP4P (according to the original publication [53]), although the latter is recommended for critical cases by the original implementation of the CHARMM force field in GROMACS [53]. It is also important to note that CHARMM lipids were parametrized with TIP3P [35, 49], which diffuses faster than TIP4P [54]. Therefore, lipid diffusion is realistic only when TIP3P is used because the slower TIP4P unnaturally slows down the lipids [55], potentially leading to a liquid-to-gel phase transition at temperatures well above the experimental melting point. The 4-point OPC water model is recommended by Tian *et al.* to be combined with Amber ff19sb, although it is about one-third more computationally expensive than the TIP3P water model [45]. We adopted this recommendation and additionally used the 12-6 HFE parameter set for ions in simulations with Amber ff19sb [56].

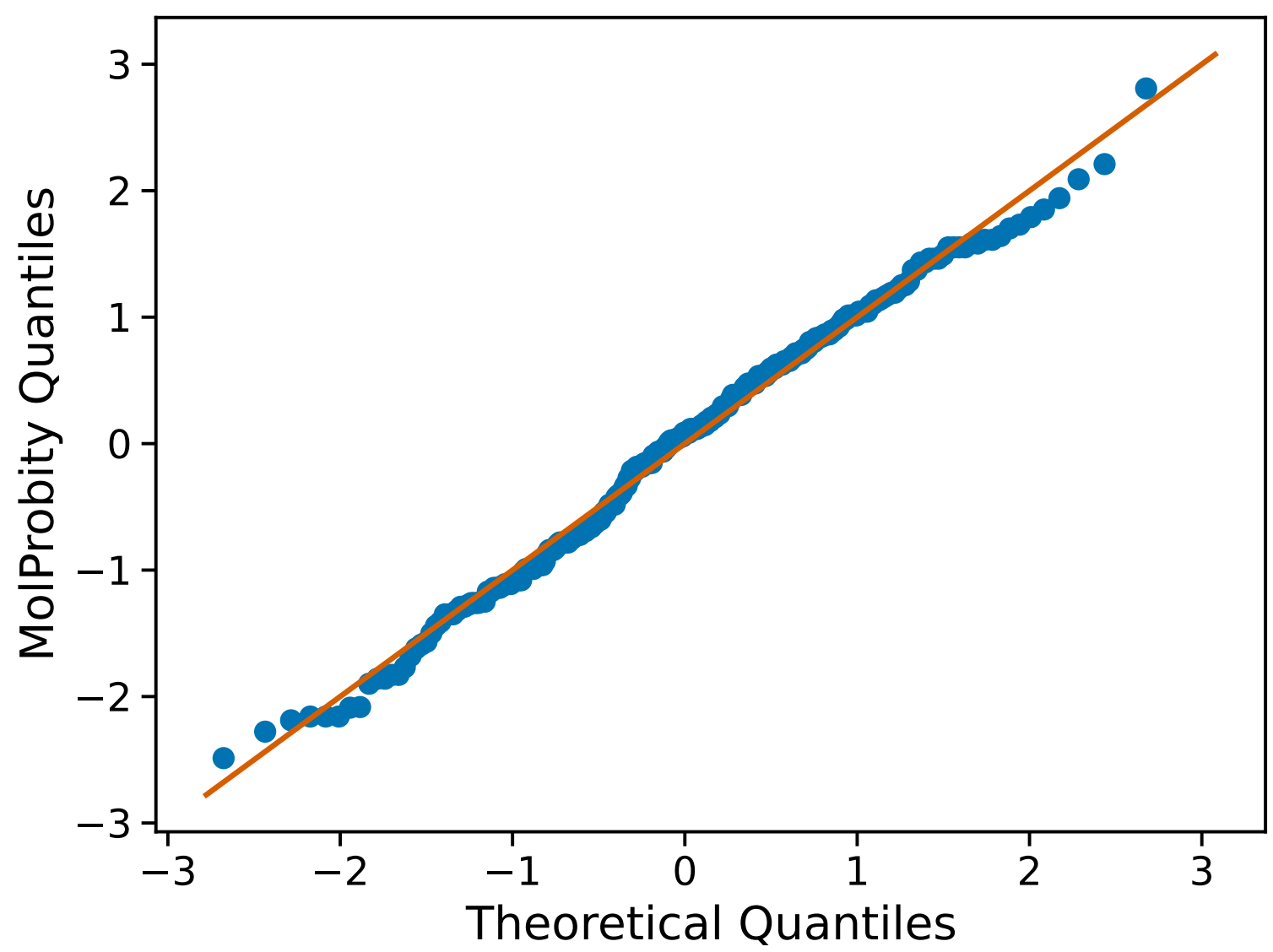

Figure S2: Quantile-quantile plot of MolProbity. For Set268, the quantiles of MolProbity after standardization are plotted against those of a standard normal distribution. The data points are in close proximity to the identity line (red), indicating that MolProbity approximately follows a normal distribution.

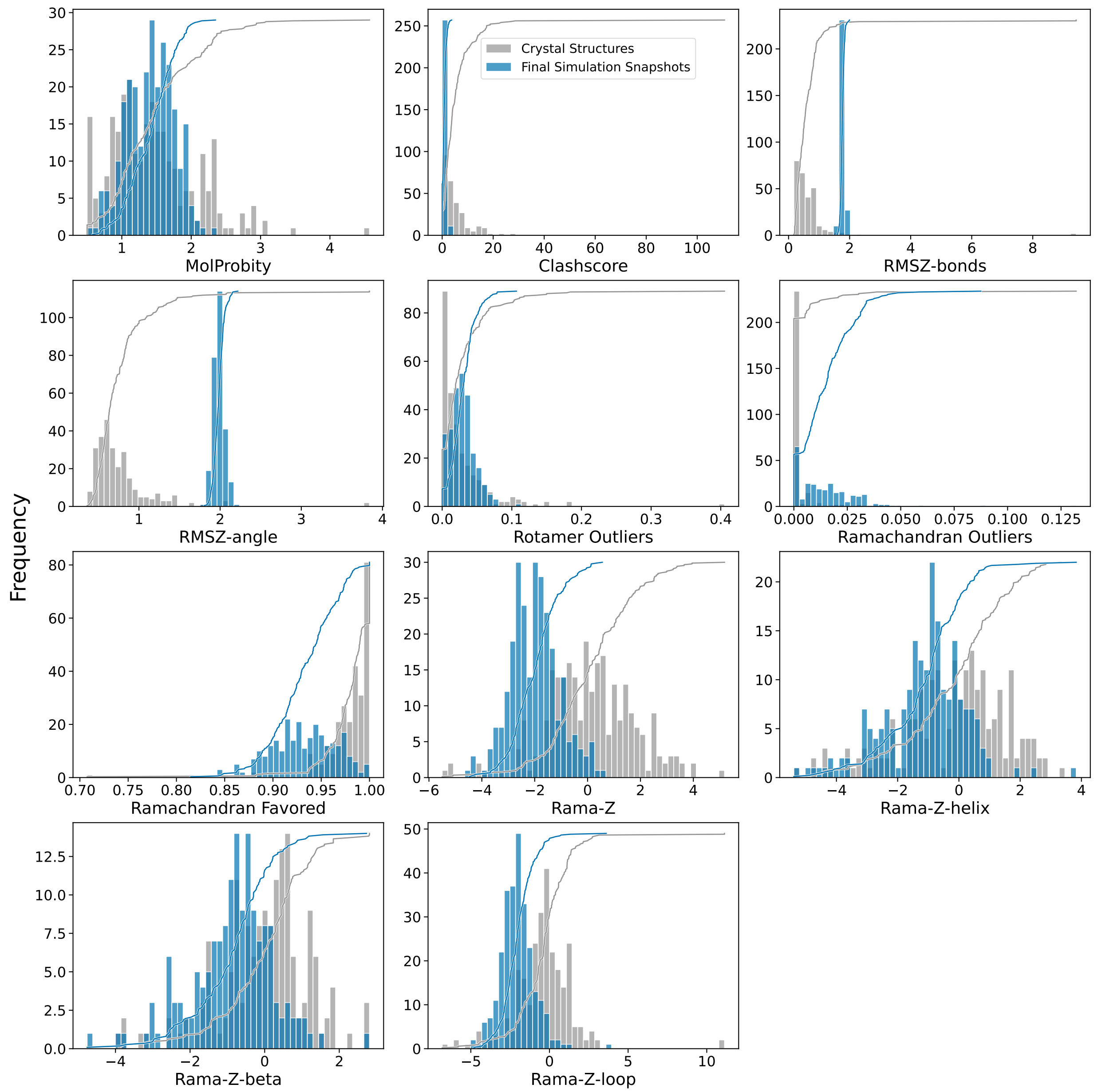

Figure S3: MolProbity subscore distributions based on Set268. The distributions for the final snapshots are shown in blue, whereas those of the corresponding starting structures (i.e., crystal structures) are depicted in grey. Cumulative distribution functions are displayed as thin lines in the same colors.

S4 Correlation Between Scores and Protein Properties

We examine whether scores are significantly correlated with protein properties, as high correlations would imply that the scores partly reflect these protein properties rather than purely assessing structural differences. Moreover, high correlations would alter the interpretation of the scores, as proteins with certain properties might systematically yield higher or lower score values. The considered properties are the number of residues per chain, the resolution of the experimentally resolved structure, the number of disulfide bonds, and the secondary structure content, specifically the percentage of residues categorized as helix (α-helix, 3_{10} helix, π-helix, or PPII), beta (β-bridge or β-sheet), or unstructured (turn, bend, or loop) by DSSP [21, 22]. Non-physiological proteins are excluded as their scores are systematically lower and therefore could bias this assessment. The resulting Pearson correlation matrix is depicted in Figure S4.

Most Pearson correlation coefficients are close to zero with two exceptions. First, TM-score and the number of residues are slightly correlated ($r = 0.34$). This is surprising, as it was originally designed to overcome precisely this characteristic of RMSD [4]. On the other hand, a length dependence of the TM-score was already observed for predicted structures in a study by Olechnovič *et al.* [10], and together with our findings, this suggests that larger proteins tend to yield higher TM-scores in practical applications. Interestingly, RMSD is not correlated with the number of residues ($r = -0.03$), possibly due to the correlated, non-random nature of MD fluctuations, which reduces the probability of small local perturbations propagating into large global conformational changes, compared to random sampling of the conformational space, a method used to estimate the relationship between RMSD and protein size previously [57].

The second exception is MolProbity, where we observed, for the crystal structures, a slight correlation with the resolution at which they were resolved ($r = 0.34$). This is expected because, according to the initial publication, a MolProbity value reflects the crystallographic resolution at which structures with comparable geometric quality would typically be observed [17, 18]. Moreover, MolProbity of the final simulation snapshots was moderately correlated with the secondary structure content (helix: $r = -0.61$, beta: $r = 0.5$, unstructured: $r = 0.46$). This must have been either induced or revealed by the MD simulations since MolProbity of the crystal structures is uncorrelated with the secondary structure content. Inspection of the proteins' property histograms shown in Figure S1 and the score-property pair plots depicted in Figure S5 reveals that proteins with no helix or beta content are comparatively abundant, thus having a disproportionate influence on the correlations without providing relevant information. Excluding these proteins reduces the correlations to $r = -0.41$, $r = 0.28$, and $r = 0.17$ for helix, beta, and unstructured, respectively. Nevertheless, a slight correlation persists, and its underlying cause remains to be investigated.

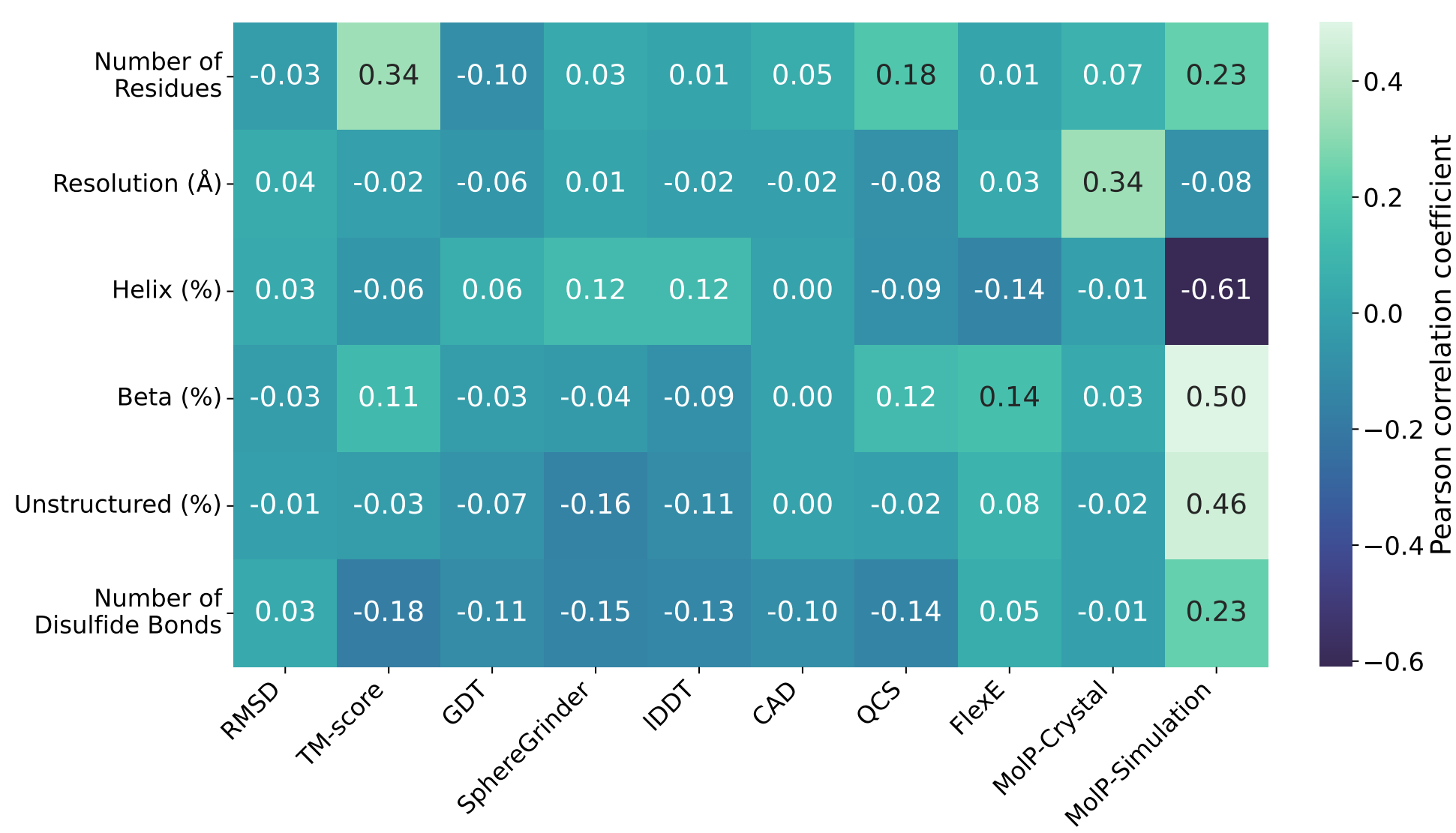

Figure S4: Pearson correlation matrix of protein properties and scores. Correlations are based on the physiological proteins in Set268. MolP: abbreviation for MolProbity.

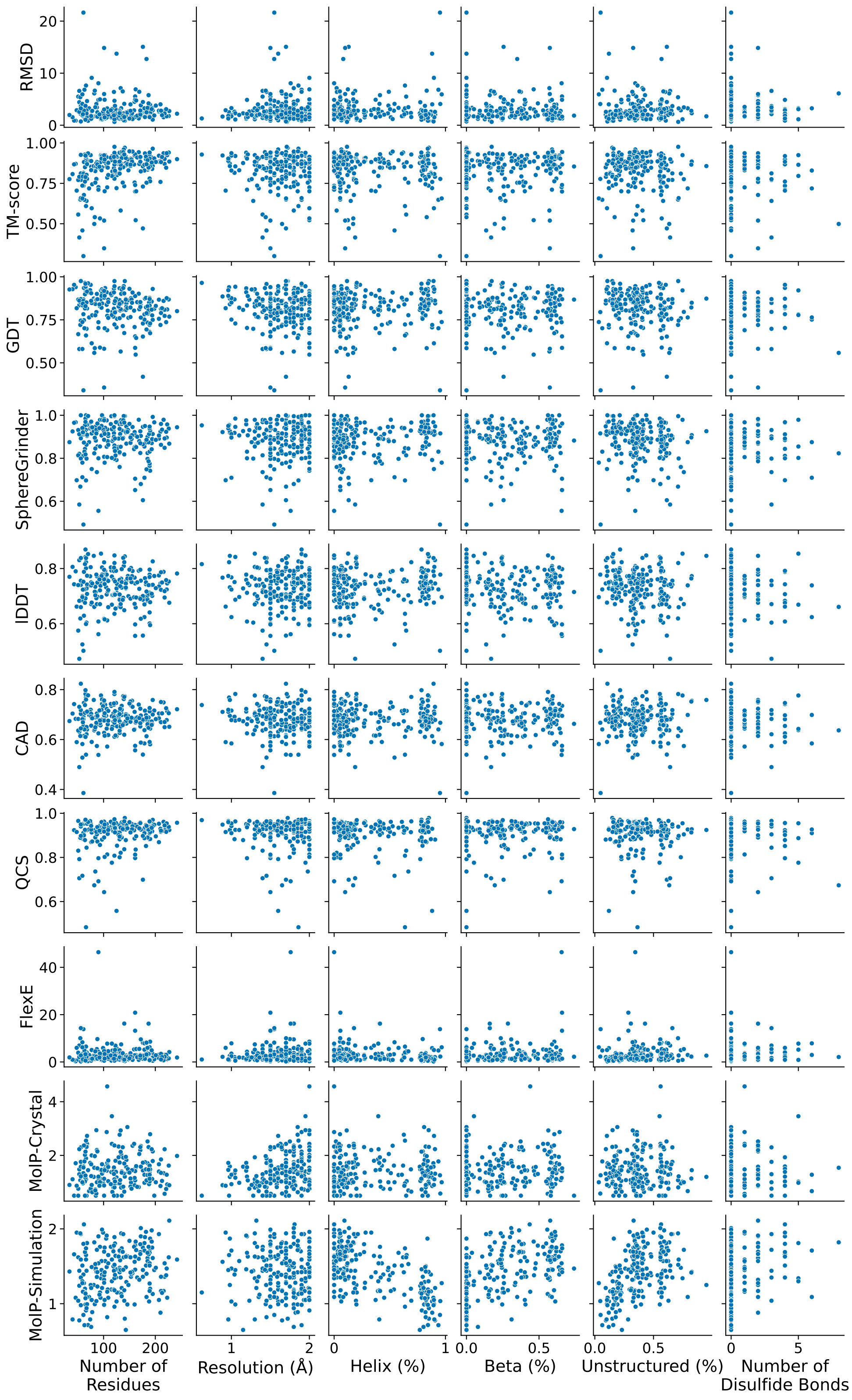

Figure S5: Pair plots between scores and protein properties based on Set268. Resolution refers to the level of detail at which each protein structure was experimentally resolved. Helix is the percentage of residues in each protein classified as α-helix, 3_{10} helix, π-helix, or PPII helix by DSSP [21, 22]. Beta is the percentage of residues in each protein classified as β-bridge or β-sheet by DSSP. Unstructured is the percentage of residues in each protein classified as turn, bend, or loop by DSSP. RMSD values are given in Å and FlexE in kcal/mol/residue.

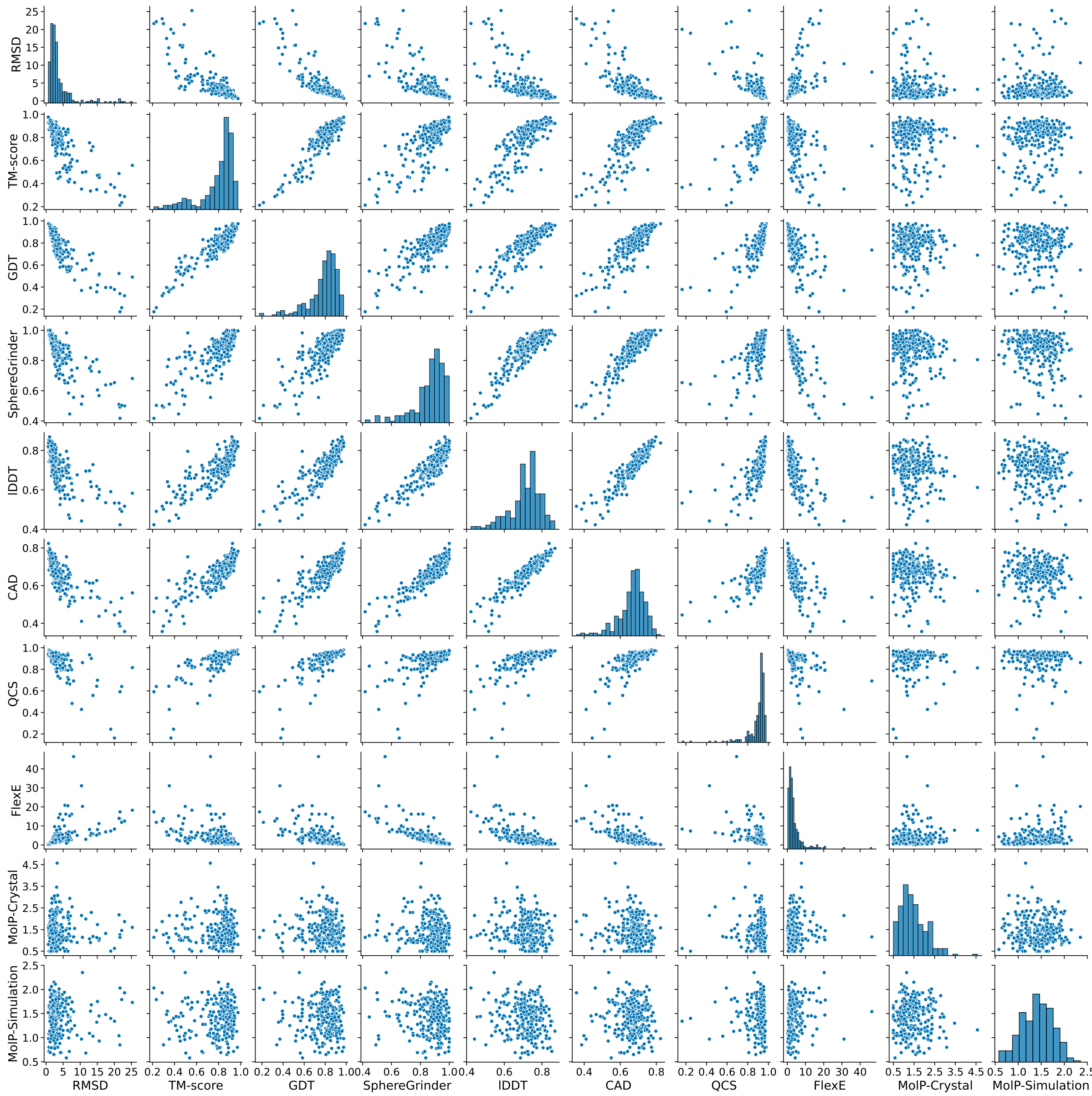

Figure S6: Pair plots between scores based on Set268. RMSD values are given in Å and FlexE in kcal/mol/residue.

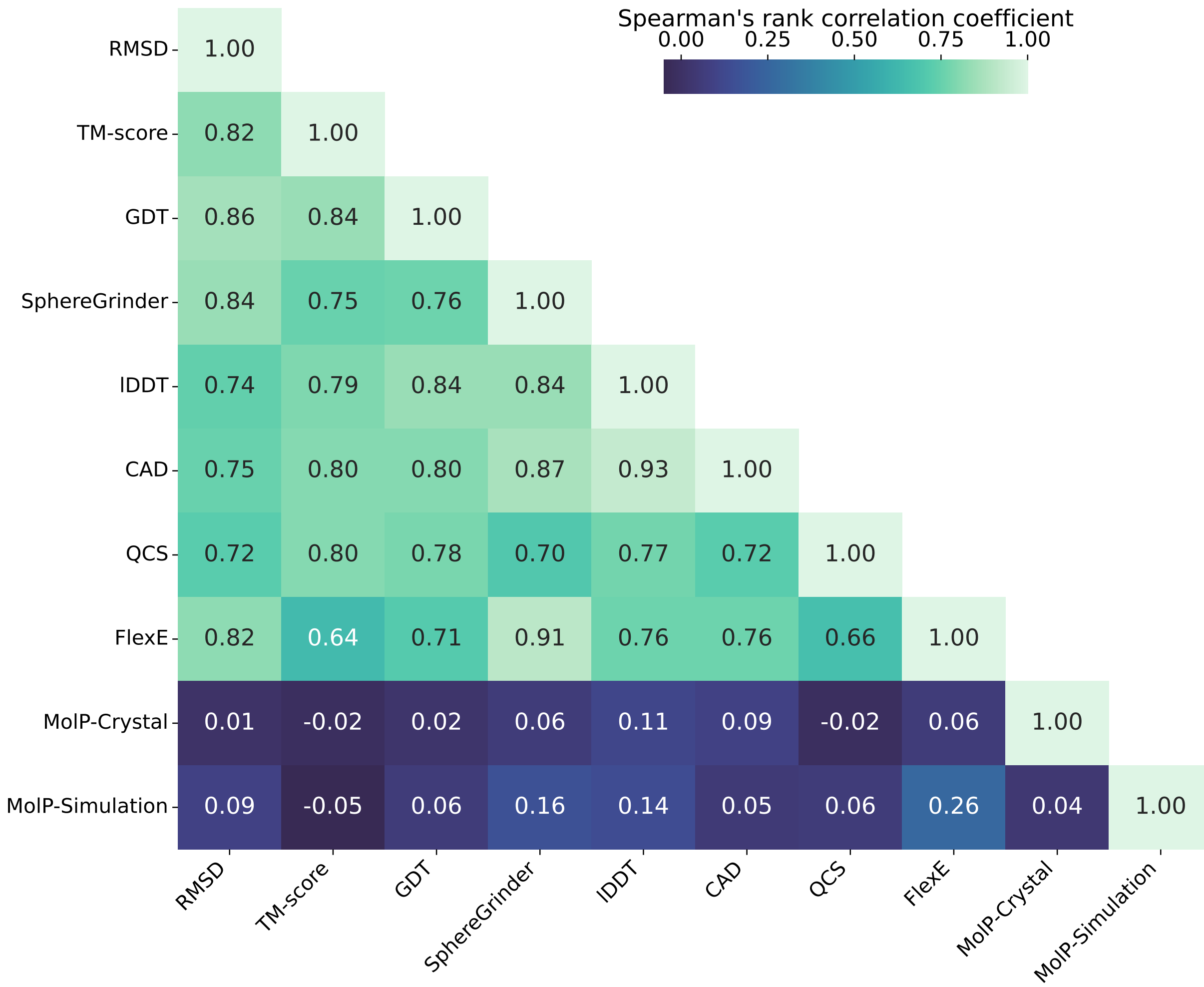

Figure S7: Spearman correlation matrix of all scores based on Set268. The signs of correlations involving RMSD, FlexE, or MolProbity are reversed to ensure consistent interpretation: positive correlation coefficients indicate that higher-similarity (higher-quality for MolProbity) values in one score tend to be associated with higher-similarity values in the other, and vice versa for negative correlation coefficients.

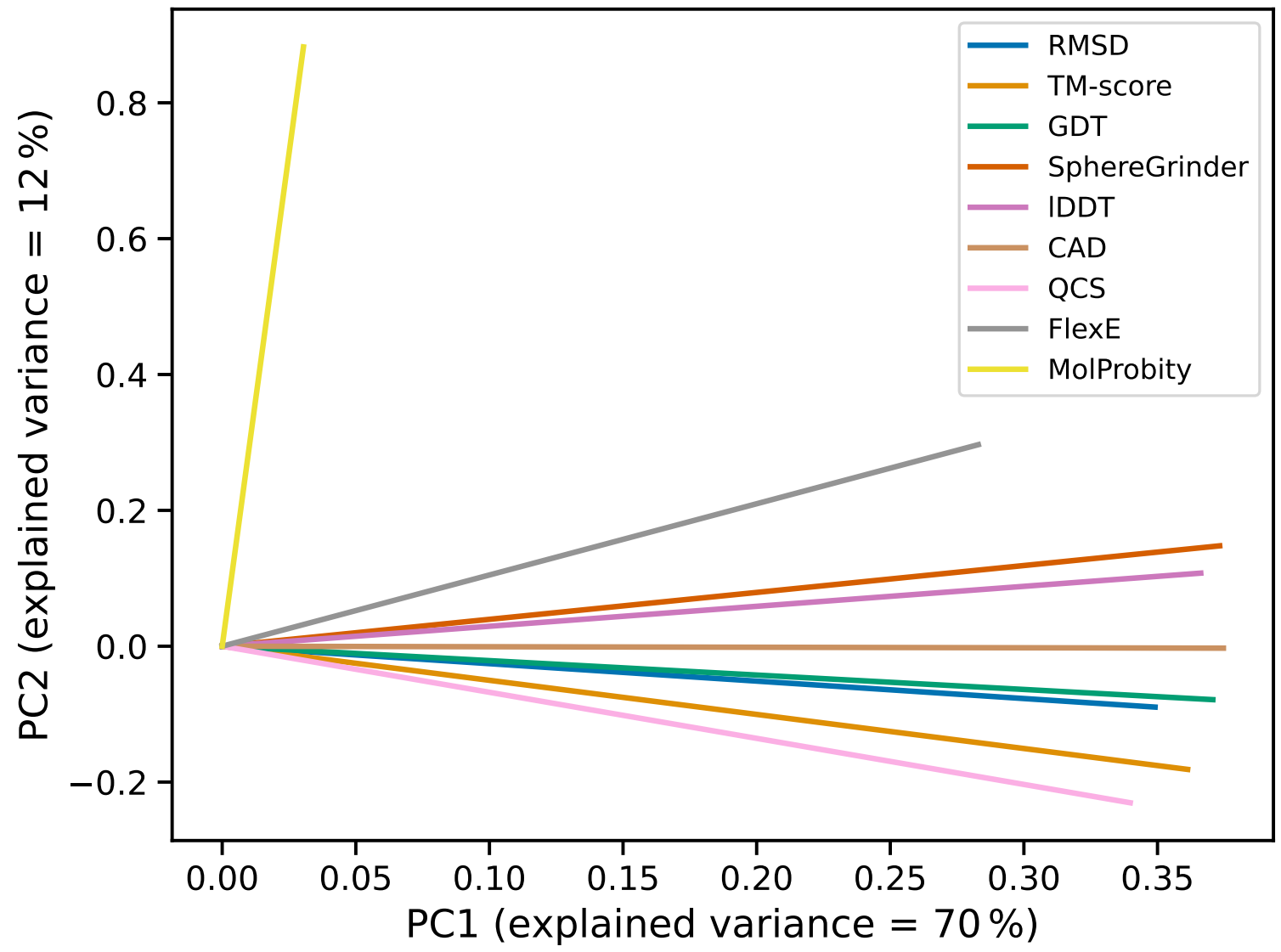

Figure S8: PCA loadings plot of scores including MolProbity based on Set268. Scores were standardized (mean zero, variance one), missing QCS values were imputed with `scikit-learn's KNNImputer`, and signs of RMSD, FlexE, and MolProbity were reversed. MolProbity has by far the highest contribution to the second principal component, with a loading of 0.88, while all other scores have absolute loadings below 0.3. Therefore, MolProbity provides largely unique, non-redundant information in terms of variance relative to all other scores.

S5 Selection of Optimal Score Combinations - OLS, Coefficient of Determination, and Explained Variance Interpretation

This analysis is based on dataset $X \in \mathbb{R}^{268\times 8}$, with the 268 proteins as observations and the eight reference-based score values of the corresponding final simulation snapshots of C36m–Set268–(n=268) as features. The considered reference-based scores are RMSD, TM-score, GDT, SphereGrinder, lDDT, CAD, QCS, and FlexE. Assuming an ordinary least squares (OLS) model, we aim to select the k (with $k \in \{1,2,3\}$) scores of X that best predict the remaining $(8-k)$ scores of X based on the coefficient of determination. Thus, we are faced with a column subset selection problem, and while solving it is NP-complete in general, cf. [58], the small number of features k we want to select allows us to solve the problem exactly. To this end, we perform the following sequential steps separately for each $k \in \{1,2,3\}$: First, all combinations of k scores, for example, RMSD–QCS or GDT–SphereGrinder for $k = 2$, are determined, and the corresponding k-column submatrices $X_S \in \mathbb{R}^{268\times k}$ of X are formed. For each combination, the dataset is split into $X = [X_S, Y]$, where $Y \in \mathbb{R}^{268\times(8-k)}$ are the remaining columns of X, that is, the data for the remaining $(8 - k)$ scores. Subsequently, X_S and Y are preprocessed, consisting of column-wise standardization to a mean of zero and a standard deviation of one, and imputation of missing QCS values with `scikit-learn's KNNImputer` using the two nearest neighbors and the Euclidean distance if QCS is in X_S. Thereafter, the OLS model $Y = X_S B + E$, where E is the error term, is fitted to the data to obtain the coefficient matrix $B = \left(X_S^\top X_S\right)^{-1} X_S^\top Y$. The resulting orthogonal projection onto the column space of X_S is given by $P := X_S\left(X_S^\top X_S\right)^{-1} X_S^\top$, such that the predicted values are

$$\hat{Y} = X_S B = PY, \tag{E7}$$

Since P is an orthogonal projection, we conclude

$$\|Y\|_F^2 = \|PY\|_F^2 + \|(I-P)Y\|_F^2 = \|\hat{Y}\|_F^2 + \|Y-\hat{Y}\|_F^2, \tag{E8}$$

where $\|\cdot\|_F$ denotes the Frobenius norm. Using the abbreviations RSS and TSS for the residual and total sum of squares, respectively, we obtain the coefficient of determination

$$R^2_{\text{pred}} := 1 - \frac{\text{RSS}}{\text{TSS}} = 1 - \frac{\|Y-\hat{Y}\|_F^2}{\|Y-\bar{Y}\|_F^2} = 1 - \frac{\|Y-\hat{Y}\|_F^2}{\|Y\|_F^2} = \frac{\|\hat{Y}\|_F^2}{\|Y\|_F^2}, \tag{E9}$$

where $\bar{Y}$ contains the column means of Y. Since Y is centered in the preprocessing step, it follows that $\bar{Y} = 0$. The performance metric R^2_{pred} precisely represents the variance in the remaining scores Y that can be predicted from and explained by X_S.

By construction, $\hat{Y} = PY$ is a matrix of rank at most k. Thus, the Eckart–Young–Schmidt–Mirsky theorem (e.g., [59, Thm. 3.6]) implies

$$\min_{\text{rank}(\tilde{Y})\le k} \|Y-\tilde{Y}\|_F \le \|Y-\hat{Y}\|_F \tag{E10}$$

and the left-hand side is minimized by the projection onto the leading k left principal directions. Thus, the coefficient of determination is bounded above by the explained variance of PCA.

Apart from that, we are also interested in the amount of the total variance in X that can be explained by X_S. Using $\hat{X} = PX = [X_S, \hat{Y}]$, which is due to the fact that $PX_S = X_S$, we conclude $\|X\|_F^2 = \|\hat{X}\|_F^2 + \|X-\hat{X}\|_F^2$ (following the same reasoning as before). Therefore, the total variance in X that can be explained by X_S is given by

$$R^2_{\text{all}} = 1 - \frac{\|X-\hat{X}\|_F^2}{\|X-\bar{X}\|_F^2} = \frac{\|\hat{X}\|_F^2}{\|X\|_F^2}. \tag{E11}$$

Again, observing that $\text{rank}(PX) \le k$, we conclude that also R^2_{all} is bounded above by the explained variance of PCA. Finally, R^2_{pred} and R^2_{all} are compared between all combinations of k scores, and the best performing ones are selected.

QCS contains 32 missing values that require specific handling: If QCS is in X_S, the missing values are imputed based on the two nearest neighbors, and they are exclusively used to train the model. The imputed values do not contribute to R^2_{all}, because their actual values are undefined and could distort the performance metric. If $k = 1$ and QCS is the only score in X_S, imputation based on the nearest neighbors is impossible, and the corresponding observations are removed from the training and evaluation process. On the other hand, if the missing QCS values are in Y, they are not imputed, they are not considered during training, and they do not contribute to the performance metrics.

The previously described process evaluates the training error of the OLS model for each combination of k scores via R^2_{pred} and R^2_{all}. However, the training error typically provides an overly optimistic estimate of model performance on unseen data. Thus, we additionally performed 10-fold cross-validation to obtain a more realistic estimate. For each fold, the data matrix $X = [X_S, Y]$ is split into the training data $[X_S^{\text{train}}, Y^{\text{train}}]$ and the validation data $[X_S^{\text{val}}, Y^{\text{val}}]$ sets. The preprocessing and the model training are performed as previously described on $[X_S^{\text{train}}, Y^{\text{train}}]$. Then, the identical preprocessings used for X_S^{train} and Y^{train} are applied to X_S^{val} and Y^{val}, respectively, to avoid data leakage. Subsequently, the prediction and the evaluation via R^2_{pred} or R^2_{all} is performed as previously described on the validation set. The final model performance is then obtained by computing the median over the 10 folds. In cross-validation, the variance decomposition into explained and unexplained parts does not hold exactly because $\hat{Y}^{\text{val}}$ is not the orthogonal projection of Y^{val} onto the column space of X_S^{val}. Rather, it is obtained by applying the projection operator estimated from X_S^{train}. Nevertheless, the interpretation in terms of variance is still reasonable in practical applications, given that the training and validation data are drawn from the same underlying distribution.

Table S2: Predictive performance of individual scores. The performance of individual scores in predicting the remaining scores is ranked in descending order via R^2_{all} and R^2_{pred} for the training set (train) and for 10-fold cross-validation (10-fold).

Scores	R^2_{all} (train)	Scores	R^2_{all} (10-fold)	Scores	R^2_{pred} (train)	Scores	R^2_{pred} (10-fold)
CAD	0.692	CAD	0.661	CAD	0.648	GDT	0.621
SphereGrinder	0.691	GDT	0.657	SphereGrinder	0.646	SphereGrinder	0.610
GDT	0.686	SphereGrinder	0.655	GDT	0.640	CAD	0.609
lDDT	0.671	lDDT	0.653	lDDT	0.624	lDDT	0.602
TM-score	0.656	TM-score	0.623	TM-score	0.606	TM-score	0.568
RMSD	0.603	RMSD	0.545	RMSD	0.546	RMSD	0.504
QCS	0.541	QCS	0.537	QCS	0.475	QCS	0.468
FlexE	0.439	FlexE	0.401	FlexE	0.357	FlexE	0.366

Table S3: Predictive performance of score pairs. The performance of two scores in predicting the remaining scores is ranked in descending order via R^2_{all} and R^2_{pred} for the training set (train) and for 10-fold cross-validation (10-fold).

Scores	R^2_{all} (train)	Scores	R^2_{all} (10-fold)	Scores	R^2_{pred} (train)	Scores	R^2_{pred} (10-fold)
GDT–SphereGrinder	0.813	GDT–FlexE	0.793	GDT–SphereGrinder	0.749	GDT–FlexE	0.723
GDT–FlexE	0.812	TM-score–FlexE	0.791	GDT–FlexE	0.748	RMSD–lDDT	0.720
TM-score–SphereGrinder	0.810	RMSD–lDDT	0.786	TM-score–SphereGrinder	0.746	GDT–SphereGrinder	0.708
TM-score–FlexE	0.807	RMSD–CAD	0.777	TM-score–FlexE	0.741	TM-score–FlexE	0.705
RMSD–lDDT	0.804	GDT–SphereGrinder	0.771	RMSD–lDDT	0.737	RMSD–CAD	0.704
GDT–CAD	0.791	TM-score–SphereGrinder	0.770	GDT–CAD	0.721	TM-score–SphereGrinder	0.696
RMSD–CAD	0.789	TM-score–CAD	0.767	SphereGrinder–QCS	0.718	lDDT–QCS	0.688
TM-score–CAD	0.787	CAD–FlexE	0.758	RMSD–CAD	0.717	CAD–FlexE	0.688
RMSD–SphereGrinder	0.786	RMSD–SphereGrinder	0.757	TM-score–CAD	0.714	GDT–CAD	0.687
SphereGrinder–QCS	0.785	GDT–CAD	0.755	RMSD–SphereGrinder	0.713	CAD–QCS	0.684
GDT–lDDT	0.784	GDT–lDDT	0.753	GDT–lDDT	0.710	RMSD–SphereGrinder	0.682
TM-score–lDDT	0.780	TM-score–lDDT	0.746	TM-score–lDDT	0.705	TM-score–CAD	0.681
CAD–FlexE	0.777	CAD–QCS	0.745	CAD–FlexE	0.701	GDT–lDDT	0.677
CAD–QCS	0.770	lDDT–QCS	0.744	CAD–QCS	0.698	TM-score–lDDT	0.662
lDDT–QCS	0.766	RMSD–FlexE	0.743	lDDT–QCS	0.693	SphereGrinder–QCS	0.661
lDDT–FlexE	0.756	SphereGrinder–QCS	0.741	lDDT–FlexE	0.673	SphereGrinder–FlexE	0.650
SphereGrinder–FlexE	0.751	lDDT–FlexE	0.737	SphereGrinder–FlexE	0.666	lDDT–FlexE	0.646
SphereGrinder–CAD	0.751	GDT–QCS	0.723	SphereGrinder–CAD	0.666	GDT–QCS	0.641
SphereGrinder–lDDT	0.748	SphereGrinder–FlexE	0.722	SphereGrinder–lDDT	0.662	SphereGrinder–lDDT	0.631
GDT–QCS	0.740	SphereGrinder–lDDT	0.709	GDT–QCS	0.658	RMSD–FlexE	0.630
RMSD–FlexE	0.738	SphereGrinder–CAD	0.709	RMSD–FlexE	0.649	SphereGrinder–CAD	0.615
RMSD–GDT	0.735	lDDT–CAD	0.703	RMSD–GDT	0.644	RMSD–GDT	0.598
RMSD–TM-score	0.732	TM-score–GDT	0.696	RMSD–TM-score	0.641	TM-score–QCS	0.592
lDDT–CAD	0.724	RMSD–GDT	0.693	lDDT–CAD	0.631	RMSD–TM-score	0.585
TM-score–QCS	0.717	TM-score–QCS	0.684	TM-score–QCS	0.628	TM-score–GDT	0.580
TM-score–GDT	0.713	RMSD–TM-score	0.683	TM-score–GDT	0.615	lDDT–CAD	0.575
RMSD–QCS	0.690	RMSD–QCS	0.672	RMSD–QCS	0.593	RMSD–QCS	0.559
QCS–FlexE	0.659	QCS–FlexE	0.613	QCS–FlexE	0.552	QCS–FlexE	0.536

Table S4: Predictive performance of score triplets. The performance of three scores in predicting the remaining scores is ranked in descending order via R^2_{all} and R^2_{pred} for the training set (train) and for 10-fold cross-validation (10-fold).

Scores	R^2_{all} (train)	Scores	R^2_{all} (10-fold)	Scores	R^2_{pred} (train)	Scores	R^2_{pred} (10-fold)
RMSD–lDDT–FlexE	0.882	GDT–lDDT–QCS	0.866	RMSD–lDDT–FlexE	0.810	GDT–lDDT–QCS	0.787
GDT–CAD–FlexE	0.876	RMSD–lDDT–QCS	0.859	GDT–CAD–FlexE	0.799	GDT–CAD–QCS	0.780
TM-score–CAD–FlexE	0.872	GDT–CAD–QCS	0.855	TM-score–CAD–FlexE	0.793	RMSD–lDDT–QCS	0.777
RMSD–CAD–FlexE	0.871	GDT–SphereGrinder–QCS	0.855	RMSD–CAD–FlexE	0.792	RMSD–lDDT–FlexE	0.770
GDT–lDDT–FlexE	0.869	TM-score–lDDT–QCS	0.853	GDT–lDDT–FlexE	0.789	TM-score–lDDT–QCS	0.768
TM-score–lDDT–FlexE	0.866	GDT–QCS–FlexE	0.851	GDT–QCS–FlexE	0.786	TM-score–CAD–FlexE	0.764
GDT–SphereGrinder–FlexE	0.864	TM-score–CAD–FlexE	0.848	TM-score–lDDT–FlexE	0.784	GDT–SphereGrinder–QCS	0.764
GDT–QCS–FlexE	0.864	RMSD–lDDT–FlexE	0.847	GDT–SphereGrinder–QCS	0.783	RMSD–CAD–FlexE	0.760
GDT–SphereGrinder–QCS	0.862	RMSD–CAD–FlexE	0.844	GDT–SphereGrinder–FlexE	0.781	TM-score–CAD–QCS	0.756
TM-score–SphereGrinder–FlexE	0.859	TM-score–SphereGrinder–QCS	0.843	TM-score–SphereGrinder–QCS	0.776	GDT–CAD–FlexE	0.754
TM-score–SphereGrinder–QCS	0.858	CAD–QCS–FlexE	0.842	CAD–QCS–FlexE	0.774	TM-score–lDDT–FlexE	0.749
RMSD–TM-score–FlexE	0.858	lDDT–QCS–FlexE	0.839	TM-score–QCS–FlexE	0.773	TM-score–SphereGrinder–QCS	0.744
RMSD–TM-score–SphereGrinder	0.857	TM-score–CAD–QCS	0.838	TM-score–SphereGrinder–FlexE	0.772	TM-score–SphereGrinder–FlexE	0.744
CAD–QCS–FlexE	0.857	TM-score–lDDT–FlexE	0.837	RMSD–TM-score–FlexE	0.770	GDT–QCS–FlexE	0.743
TM-score–QCS–FlexE	0.856	GDT–CAD–FlexE	0.837	RMSD–TM-score–SphereGrinder	0.769	GDT–lDDT–FlexE	0.742
RMSD–GDT–FlexE	0.853	RMSD–TM-score–FlexE	0.835	RMSD–lDDT–QCS	0.764	CAD–QCS–FlexE	0.741
RMSD–GDT–SphereGrinder	0.853	GDT–lDDT–FlexE	0.835	RMSD–GDT–FlexE	0.762	GDT–SphereGrinder–FlexE	0.740
RMSD–lDDT–QCS	0.850	RMSD–TM-score–SphereGrinder	0.829	RMSD–GDT–SphereGrinder	0.762	TM-score–QCS–FlexE	0.735
RMSD–TM-score–lDDT	0.849	RMSD–GDT–FlexE	0.828	RMSD–TM-score–lDDT	0.756	RMSD–TM-score–FlexE	0.734
GDT–SphereGrinder–lDDT	0.846	TM-score–SphereGrinder–lDDT	0.828	lDDT–QCS–FlexE	0.756	lDDT–QCS–FlexE	0.731
lDDT–QCS–FlexE	0.845	RMSD–TM-score–lDDT	0.828	GDT–SphereGrinder–lDDT	0.751	RMSD–GDT–SphereGrinder	0.730
RMSD–SphereGrinder–lDDT	0.845	GDT–SphereGrinder–FlexE	0.827	RMSD–SphereGrinder–QCS	0.751	RMSD–TM-score–lDDT	0.729
TM-score–SphereGrinder–lDDT	0.843	TM-score–GDT–FlexE	0.826	GDT–CAD–QCS	0.750	RMSD–CAD–QCS	0.726
RMSD–SphereGrinder–FlexE	0.843	RMSD–GDT–SphereGrinder	0.825	RMSD–SphereGrinder–lDDT	0.749	RMSD–TM-score–SphereGrinder	0.723
RMSD–SphereGrinder–QCS	0.842	TM-score–SphereGrinder–FlexE	0.824	SphereGrinder–QCS–FlexE	0.748	RMSD–GDT–FlexE	0.723
GDT–SphereGrinder–CAD	0.842	GDT–SphereGrinder–lDDT	0.822	TM-score–SphereGrinder–lDDT	0.747	RMSD–TM-score–CAD	0.722
GDT–CAD–QCS	0.841	TM-score–QCS–FlexE	0.821	GDT–lDDT–QCS	0.747	TM-score–SphereGrinder–CAD	0.720
RMSD–TM-score–CAD	0.841	TM-score–SphereGrinder–CAD	0.816	RMSD–SphereGrinder–FlexE	0.746	SphereGrinder–QCS–FlexE	0.720
SphereGrinder–QCS–FlexE	0.840	RMSD–SphereGrinder–QCS	0.816	GDT–SphereGrinder–CAD	0.744	RMSD–GDT–CAD	0.719
TM-score–SphereGrinder–CAD	0.840	SphereGrinder–QCS–FlexE	0.815	RMSD–TM-score–CAD	0.743	RMSD–lDDT–CAD	0.717
TM-score–GDT–FlexE	0.840	RMSD–CAD–QCS	0.814	TM-score–SphereGrinder–CAD	0.742	RMSD–SphereGrinder–lDDT	0.716
RMSD–GDT–lDDT	0.840	RMSD–TM-score–CAD	0.814	TM-score–GDT–FlexE	0.742	GDT–SphereGrinder–lDDT	0.716
GDT–lDDT–QCS	0.839	RMSD–SphereGrinder–lDDT	0.812	RMSD–GDT–lDDT	0.742	RMSD–SphereGrinder–CAD	0.711
TM-score–GDT–SphereGrinder	0.837	RMSD–lDDT–CAD	0.812	TM-score–CAD–QCS	0.742	TM-score–SphereGrinder–lDDT	0.709
TM-score–CAD–QCS	0.836	TM-score–GDT–SphereGrinder	0.810	TM-score–lDDT–QCS	0.737	GDT–SphereGrinder–CAD	0.708
RMSD–GDT–CAD	0.836	GDT–lDDT–CAD	0.810	TM-score–GDT–SphereGrinder	0.736	RMSD–SphereGrinder–QCS	0.708
RMSD–SphereGrinder–CAD	0.836	RMSD–GDT–lDDT	0.809	RMSD–CAD–QCS	0.736	GDT–lDDT–CAD	0.703
TM-score–lDDT–QCS	0.833	GDT–SphereGrinder–CAD	0.809	RMSD–GDT–CAD	0.734	TM-score–GDT–FlexE	0.702
RMSD–CAD–QCS	0.832	RMSD–SphereGrinder–CAD	0.807	RMSD–SphereGrinder–CAD	0.734	RMSD–GDT–lDDT	0.699
SphereGrinder–CAD–QCS	0.827	RMSD–GDT–CAD	0.804	SphereGrinder–CAD–QCS	0.728	RMSD–SphereGrinder–FlexE	0.695
RMSD–lDDT–CAD	0.824	RMSD–SphereGrinder–FlexE	0.804	SphereGrinder–lDDT–QCS	0.722	SphereGrinder–lDDT–QCS	0.690
SphereGrinder–lDDT–QCS	0.824	TM-score–lDDT–CAD	0.803	RMSD–lDDT–CAD	0.716	TM-score–lDDT–CAD	0.686
GDT–lDDT–CAD	0.822	SphereGrinder–CAD–QCS	0.802	GDT–lDDT–CAD	0.712	lDDT–CAD–QCS	0.683
TM-score–lDDT–CAD	0.817	RMSD–QCS–FlexE	0.797	RMSD–QCS–FlexE	0.709	TM-score–GDT–SphereGrinder	0.682
RMSD–QCS–FlexE	0.815	SphereGrinder–lDDT–QCS	0.796	TM-score–lDDT–CAD	0.705	SphereGrinder–CAD–QCS	0.682
TM-score–GDT–CAD	0.814	RMSD–TM-score–QCS	0.794	TM-score–GDT–CAD	0.700	RMSD–QCS–FlexE	0.682
TM-score–GDT–lDDT	0.808	RMSD–GDT–QCS	0.793	lDDT–CAD–QCS	0.693	TM-score–GDT–CAD	0.669
lDDT–CAD–QCS	0.805	TM-score–GDT–CAD	0.787	TM-score–GDT–lDDT	0.690	SphereGrinder–lDDT–FlexE	0.669
SphereGrinder–lDDT–FlexE	0.805	lDDT–CAD–QCS	0.786	SphereGrinder–lDDT–FlexE	0.685	TM-score–GDT–lDDT	0.664
lDDT–CAD–FlexE	0.805	TM-score–GDT–lDDT	0.783	lDDT–CAD–FlexE	0.685	lDDT–CAD–FlexE	0.656
SphereGrinder–CAD–FlexE	0.804	lDDT–CAD–FlexE	0.771	SphereGrinder–CAD–FlexE	0.684	SphereGrinder–CAD–FlexE	0.656
RMSD–GDT–QCS	0.785	SphereGrinder–CAD–FlexE	0.770	RMSD–GDT–QCS	0.661	RMSD–GDT–QCS	0.652
RMSD–TM-score–QCS	0.778	SphereGrinder–lDDT–FlexE	0.762	RMSD–TM-score–QCS	0.650	RMSD–TM-score–QCS	0.651
SphereGrinder–lDDT–CAD	0.775	TM-score–GDT–QCS	0.754	SphereGrinder–lDDT–CAD	0.637	TM-score–GDT–QCS	0.603
TM-score–GDT–QCS	0.761	SphereGrinder–lDDT–CAD	0.738	TM-score–GDT–QCS	0.624	SphereGrinder–lDDT–CAD	0.577
RMSD–TM-score–GDT	0.761	RMSD–TM-score–GDT	0.732	RMSD–TM-score–GDT	0.614	RMSD–TM-score–GDT	0.576

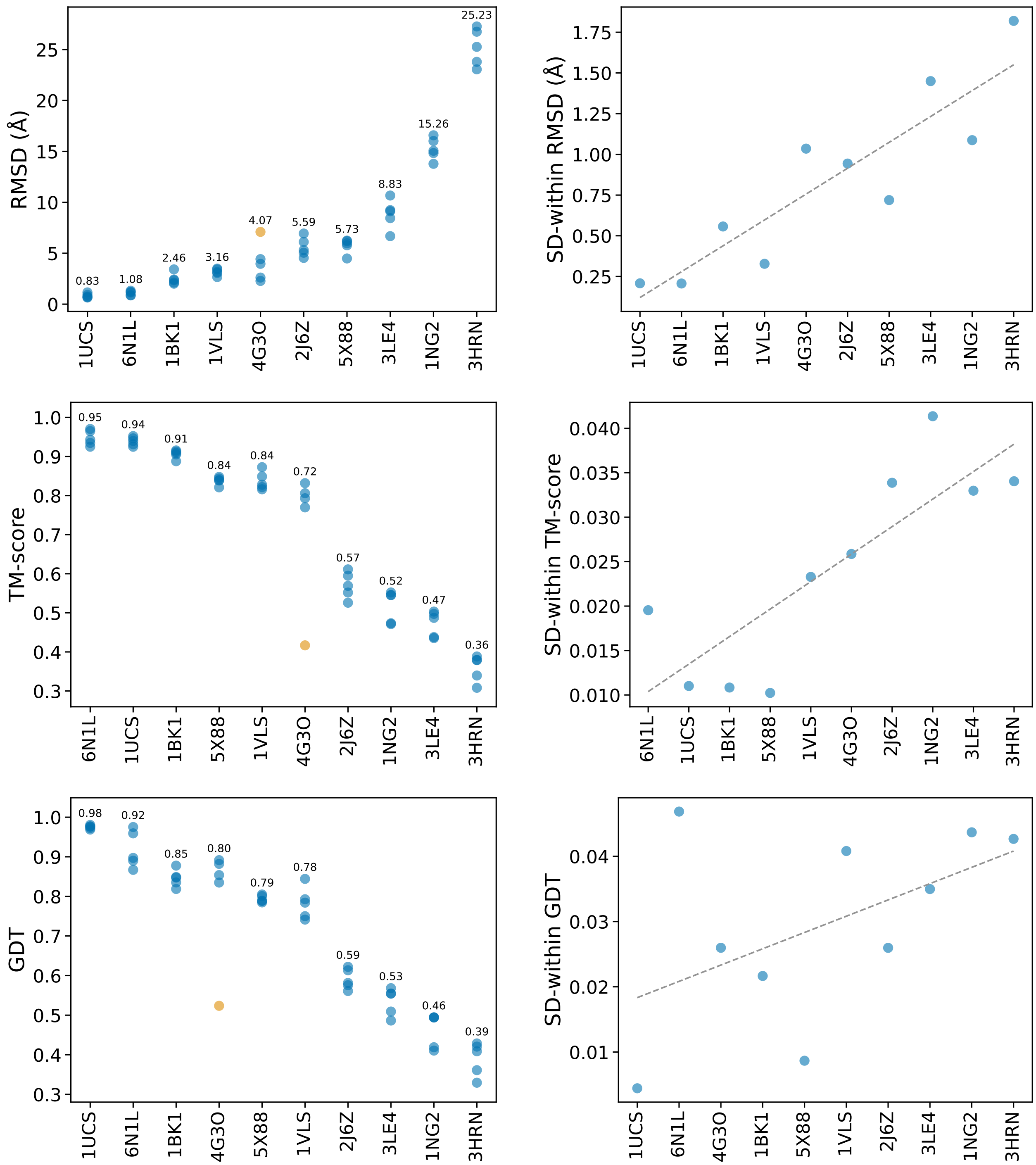

Figure S9: Scores and within-protein standard deviations of replica simulations conducted with C36m. (Left) Score values for 10 proteins selected from Set268, five replicas each. For each protein, the replicas are displayed as blue spheres, with their corresponding mean value indicated above. Proteins are sorted from highest-similarity (highest-quality for MolProbity) to lowest-similarity based on these means for each score. One replica of protein 4G3O is a potential outlier (see also Figure S10) and is highlighted in orange. QCS is undefined for 3HRN and is therefore not shown. (Right) The within-protein standard deviation (SD-within). The order of the proteins is the same as in (left). For 4GO3, the potential replica outlier was excluded from the calculation. The grey dashed line represents the OLS fit, indicating that SD-within usually increases with lower-similarity scores.

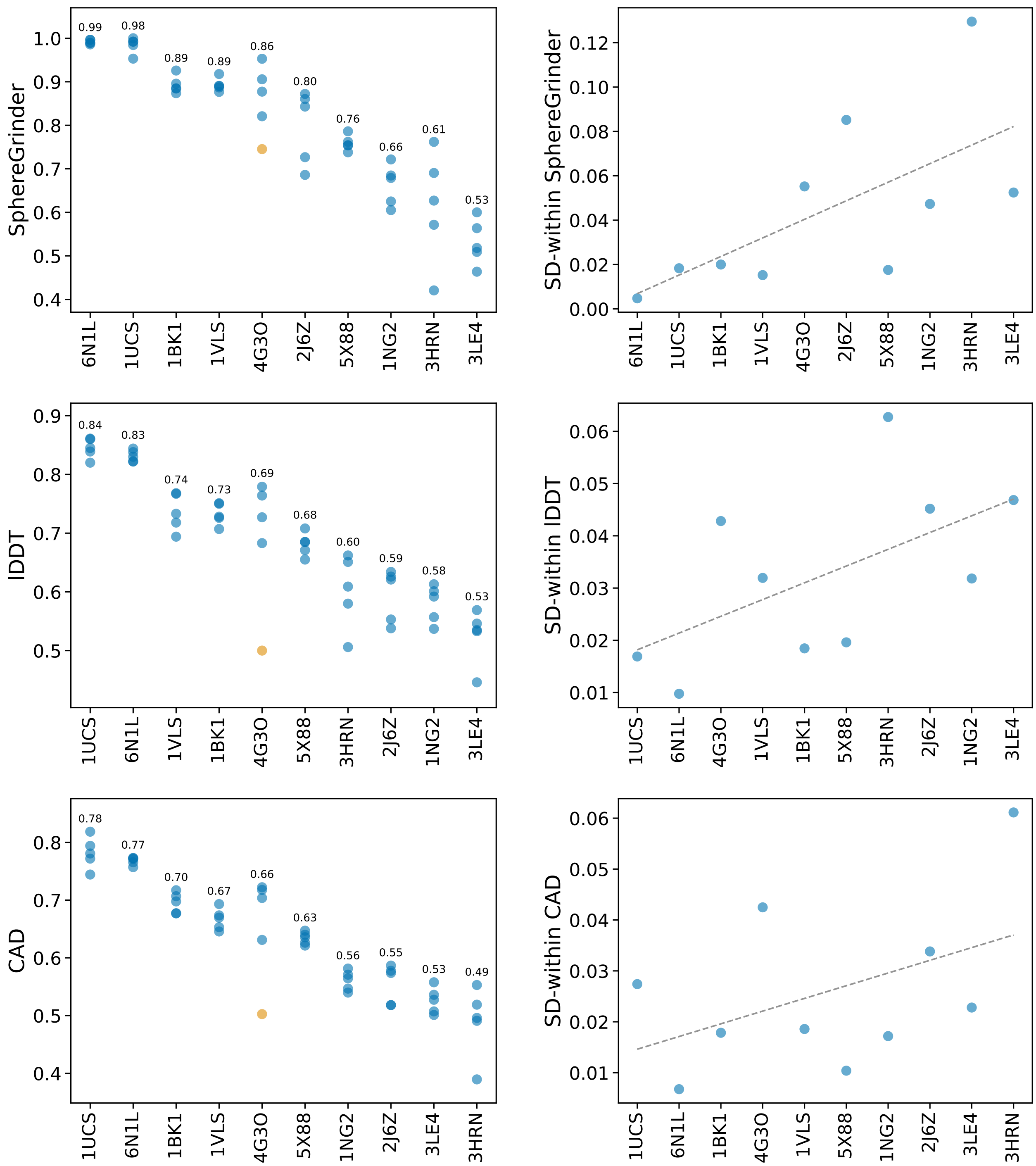

Figure S9 (continued): Scores and within-protein standard deviations of replica simulations conducted with C36m.

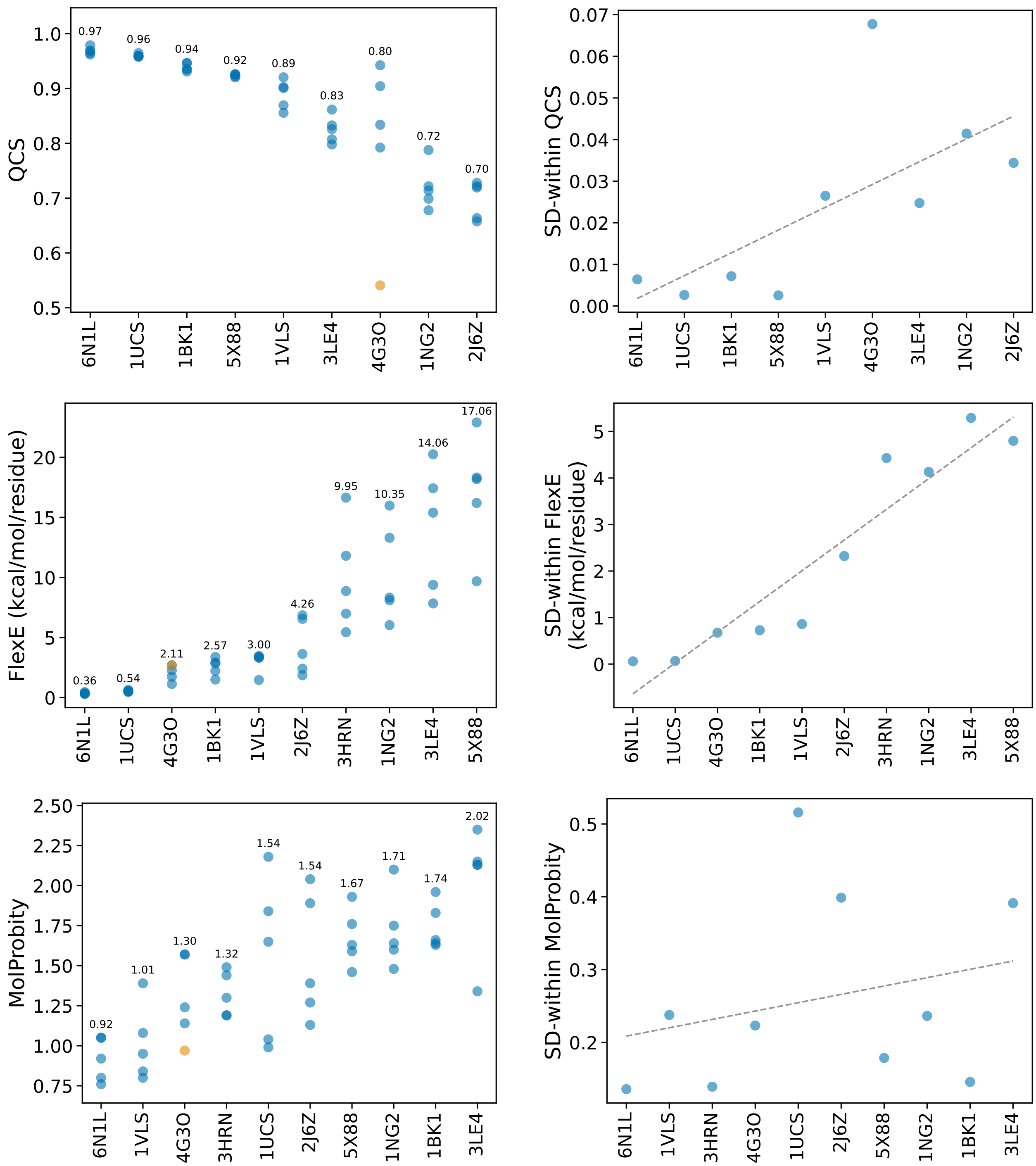

Figure S9 (continued): Scores and within-protein standard deviations of replica simulations conducted with C36m.

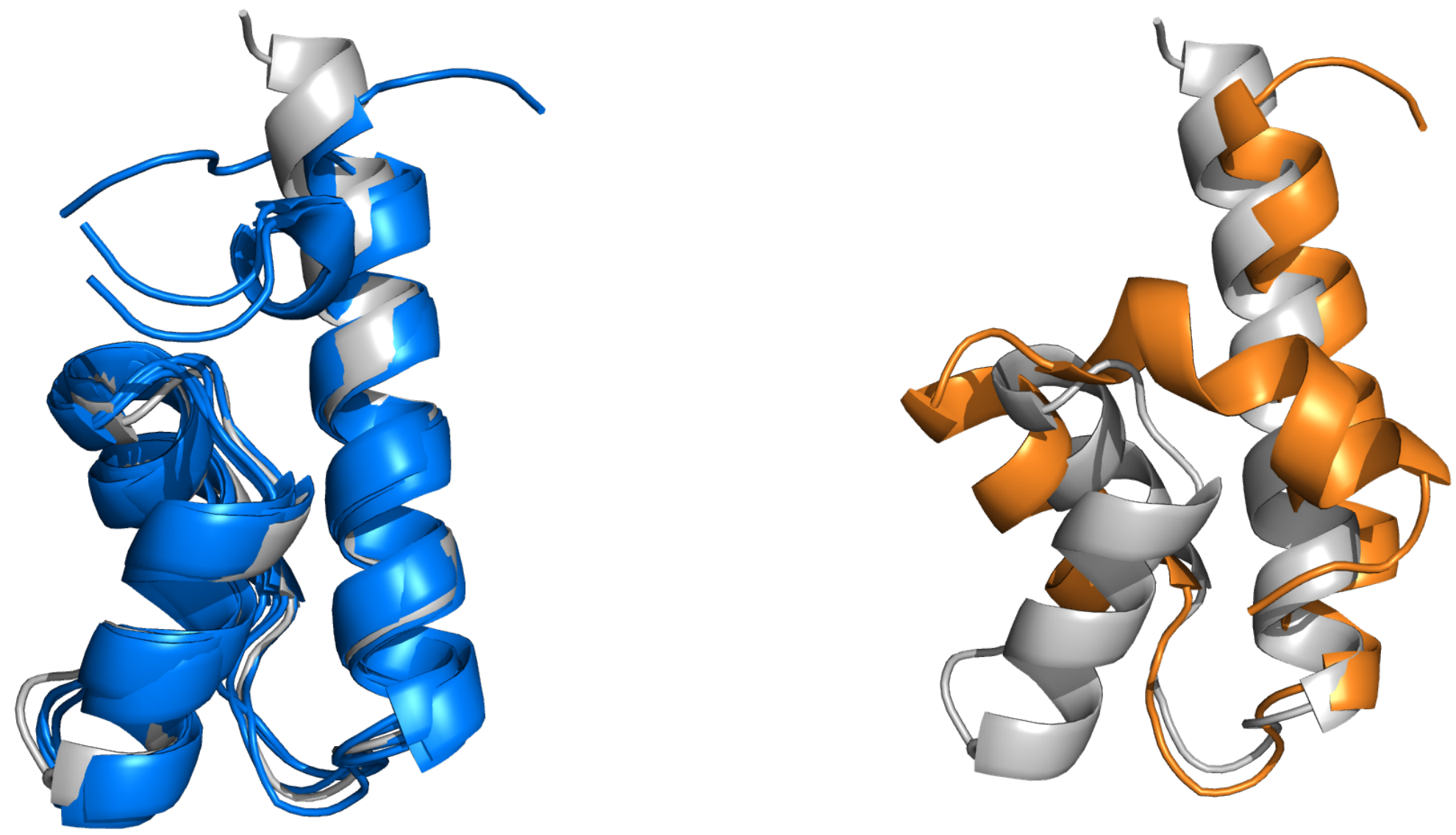

(a) (b)

Figure S10: Final snapshots of the protein 4G3O from five replica simulations using setup C36m. The crystal structure is shown in grey, the snapshots of four replica simulations in blue (a), and the potential outlier in orange (b).

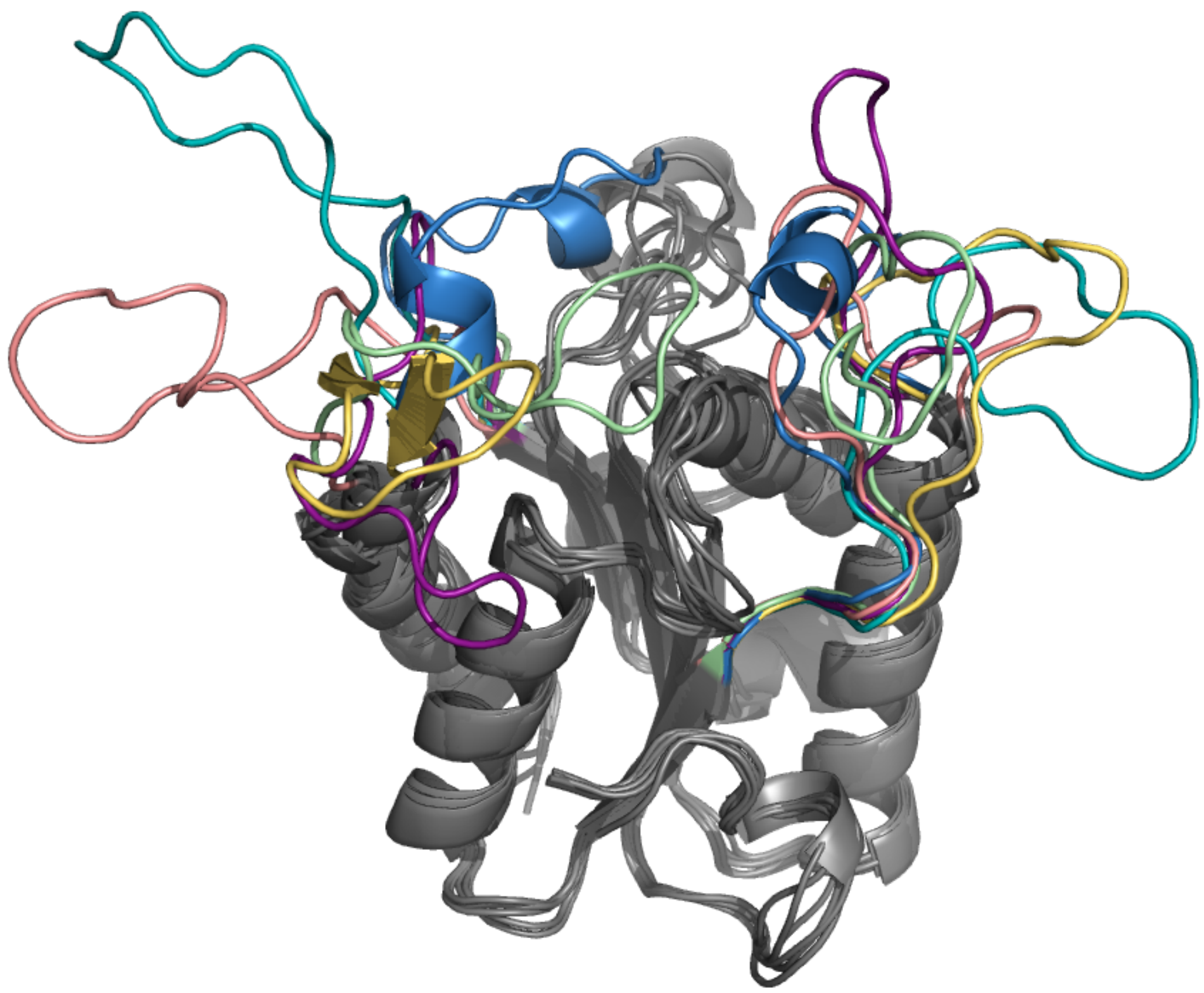

Figure S11: Final snapshots of the protein 5X88 from five replica simulations using setup C36m. The loop regions of the crystal structure are shown in blue, while the loop regions of the replicas are highlighted in red, green, cyan, purple, and yellow. The remaining protein regions are colored in grey.

S6 Decomposition of Total Score Variability for Replica Simulations

For 10 proteins, we performed five replica simulations each and evaluated the final snapshots via all nine studied scores. For each score, the total variability can be decomposed into the within-protein variability and the between-protein variability. In detail, the total sum of squares TSS of the scores can be decomposed into the within-protein sum of squares WSS and the between-protein sum of squares BSS via

$$\begin{aligned} \mathrm{TSS} &= \mathrm{WSS} + \mathrm{BSS} \\ \sum_{p=1}^{10}\sum_{r=1}^{5}(s_{pr} - \bar{s})^2 &= \sum_{p=1}^{10}\sum_{r=1}^{5}(s_{pr} - \bar{s_p})^2 + 5\sum_{p=1}^{10}(\bar{s}_p - \bar{s})^2, \end{aligned} \tag{E12}$$

where s_{pr} is the score of protein p and replica r, $\bar{s}_p$ is the score mean over all five replicas for protein p, and $\bar{s}$ is the score mean over all proteins and replicas.

S7 Correlation Between Protein Properties and Scores for Each Simulation Setup

Based on the 25 physiological proteins in Set31, we computed the Pearson correlation coefficients between the scores and the intrinsic protein properties for each of the simulation setups C36m, C36mPs3P, C36mPs4P, and A19sb (see Figure S12). Similar to the 215 physiological proteins in Set268, which were simulated using C36m, moderate to high correlations are observed between TM-score and the number of residues, MolProbity of the crystal structures and the resolution at which they were resolved, and MolProbity of the final snapshots and the secondary structure contents across all simulation setups. The remaining correlations are close to zero, though with slightly higher absolute values compared to those of the physiological proteins in Set268. This is likely due to the greatly reduced number of proteins (25 vs. 215), and not due to differences in the simulation setups, as the physiological proteins in Set31 and Set268 were both simulated using the identical simulation setup C36m. To verify this, we drew 10 000 random subsamples of 25 proteins from Set268 (physiological proteins only) and calculated the correlations between scores and properties for each subsample to estimate the distributions of correlation coefficients expected at a sample size of 25. Indeed, almost all correlation coefficients - 59, 59, 58, and 58 out of 60 for C36m, C36mPs3P, C36mPs4P, and A19sb, respectively - are within the resulting 95 % empirical reference interval, that is, the interval defined by the 0.025 and 0.975 quantiles (see Figure S13).

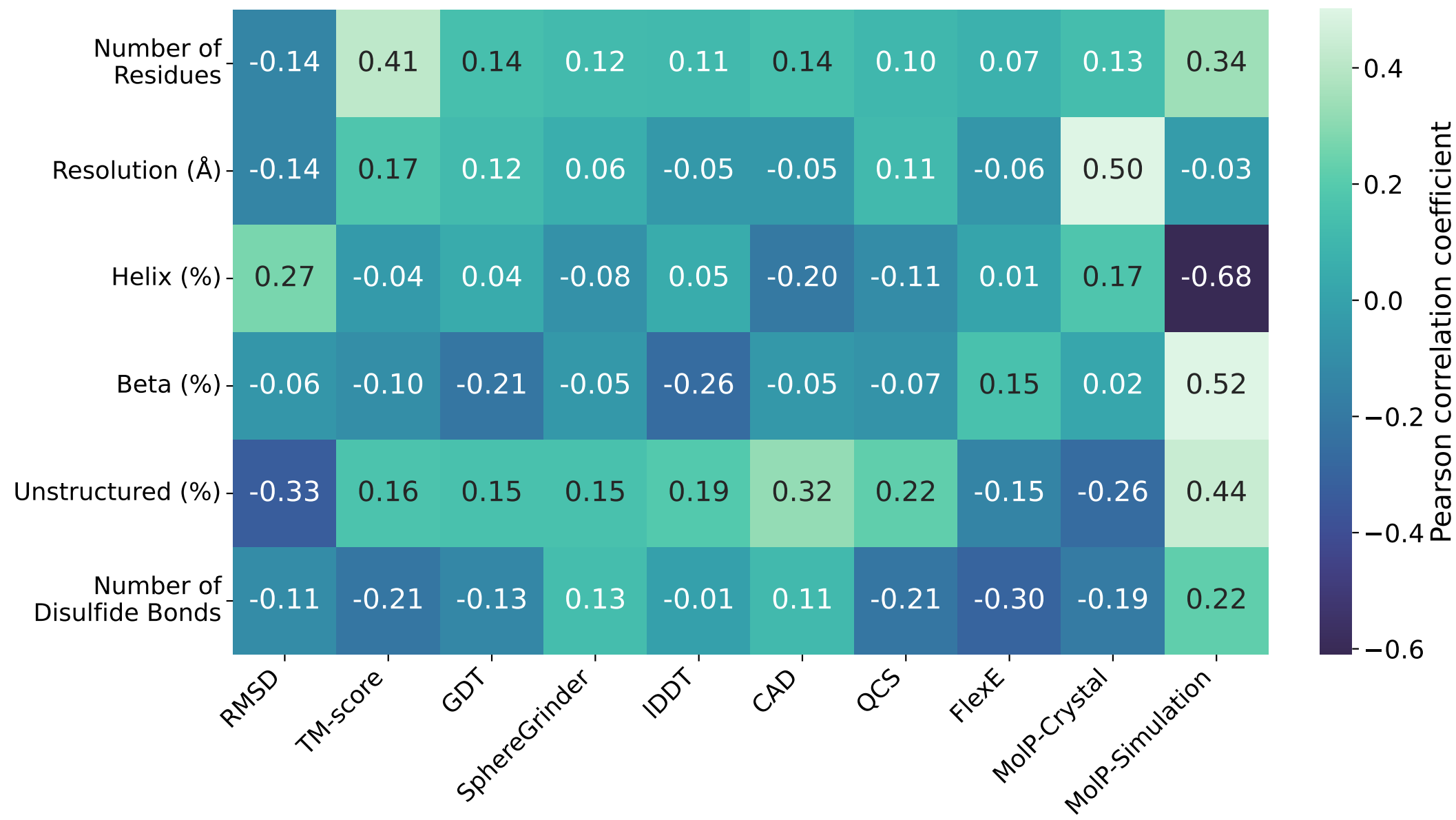

(a) C36m

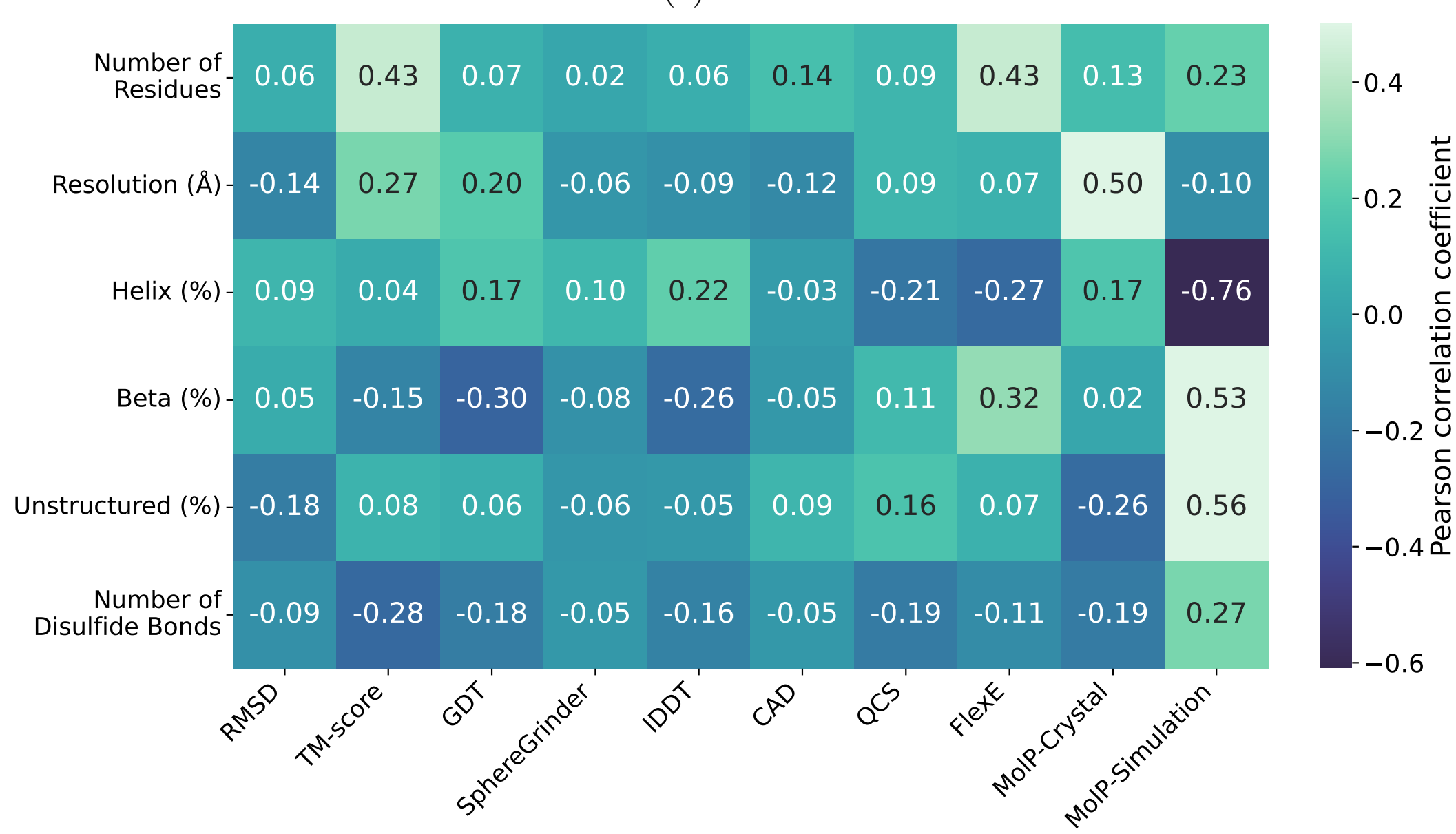

(b) C36mPs3P

Figure S12: Pearson correlations between protein properties and scores for each simulation setup. Correlations are based on the physiological proteins in Set31, and the simulation setups are C36m (a), C36mPs3P (b), C36mPs4P (c), and A19sb (d). The percentage of residues categorized as helix (α-helix, 3_{10} helix, π-helix, or PPII), beta (β-bridge or β-sheet), and unstructured (turn, bend, or loop) was calculated by DSSP [21, 22]. The correlations involving MolProbity of the crystal structures are identical across the simulation setups, as the same crystal structures were used in each case. Coloring is consistent with the correlation matrix of protein properties and scores based on the physiological proteins in Set268 (see Figure S4). MolP: abbreviation for MolProbity.

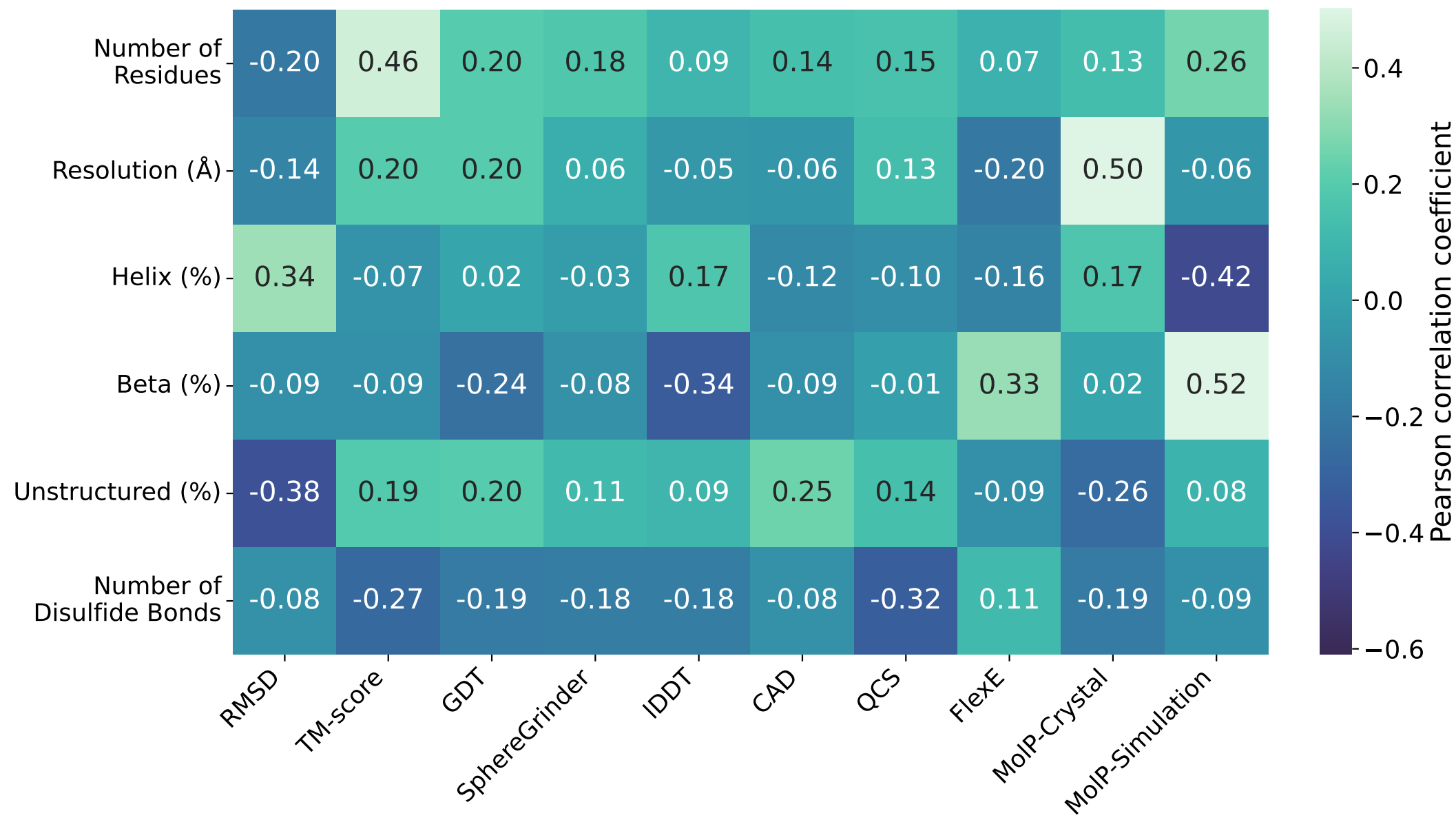

(c) C36mPs4P

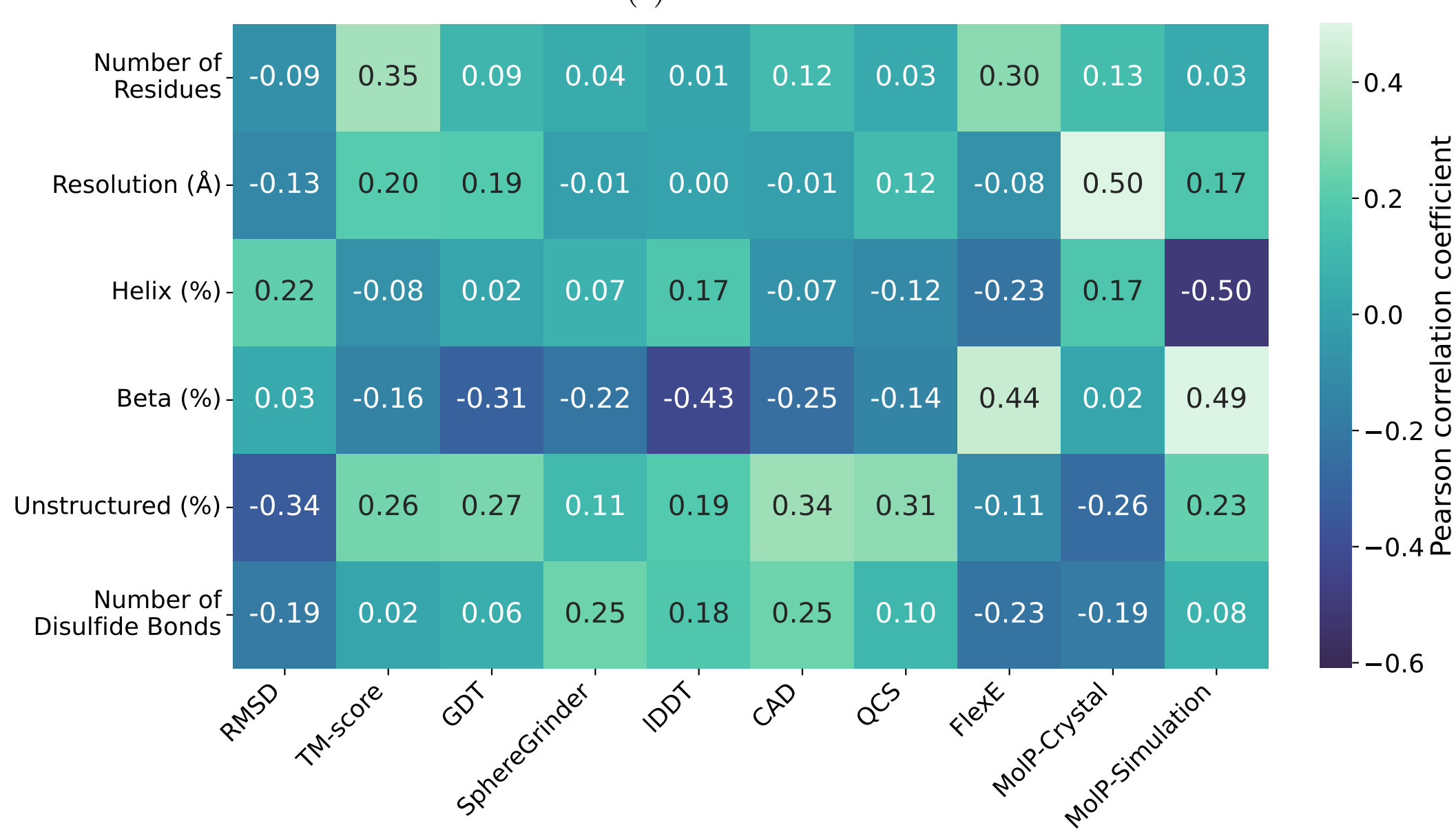

(d) A19sb

Figure S12 (continued): Pearson correlations between protein properties and scores for each simulation setup.

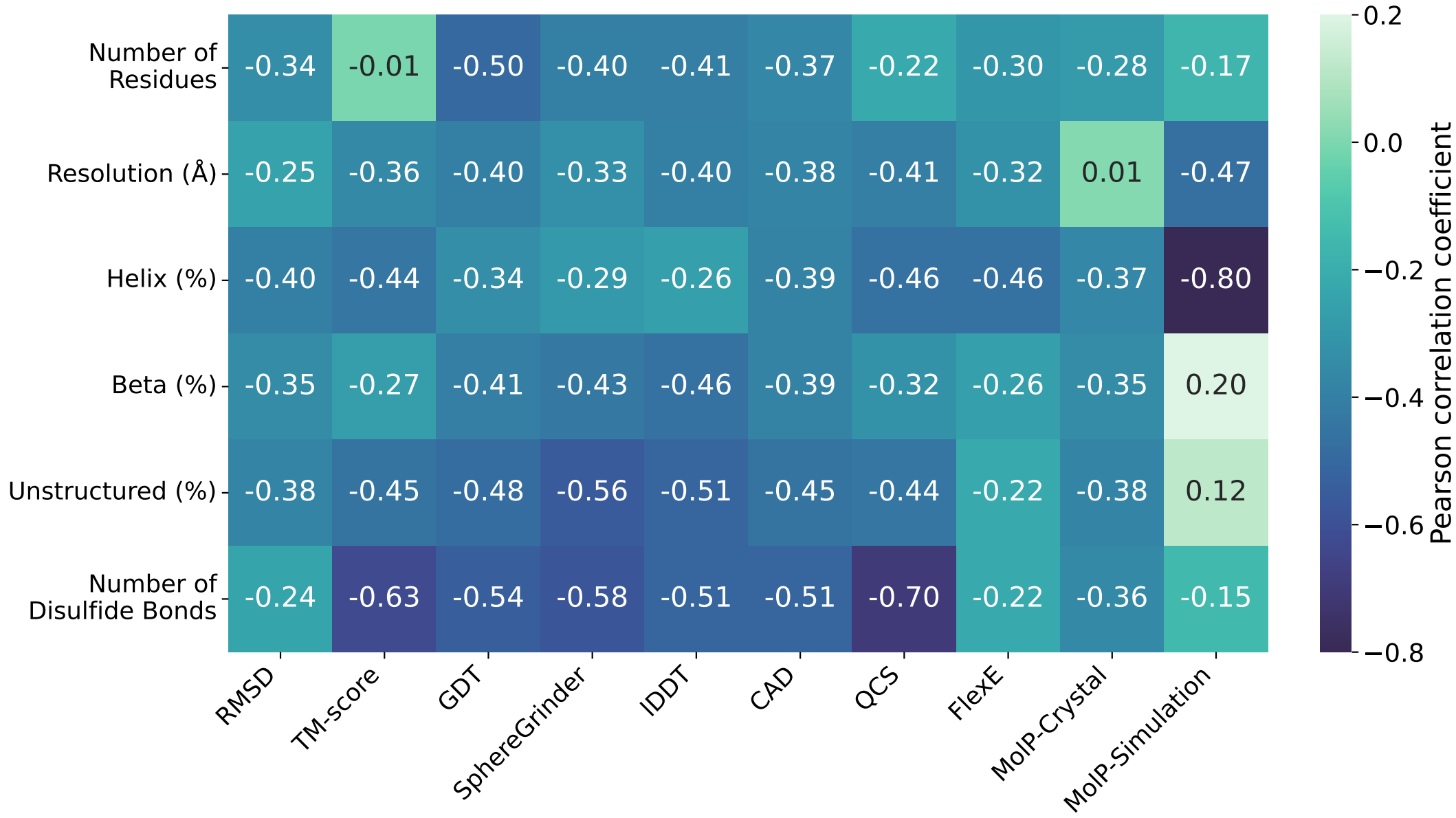

(a) 0.025 Quantiles

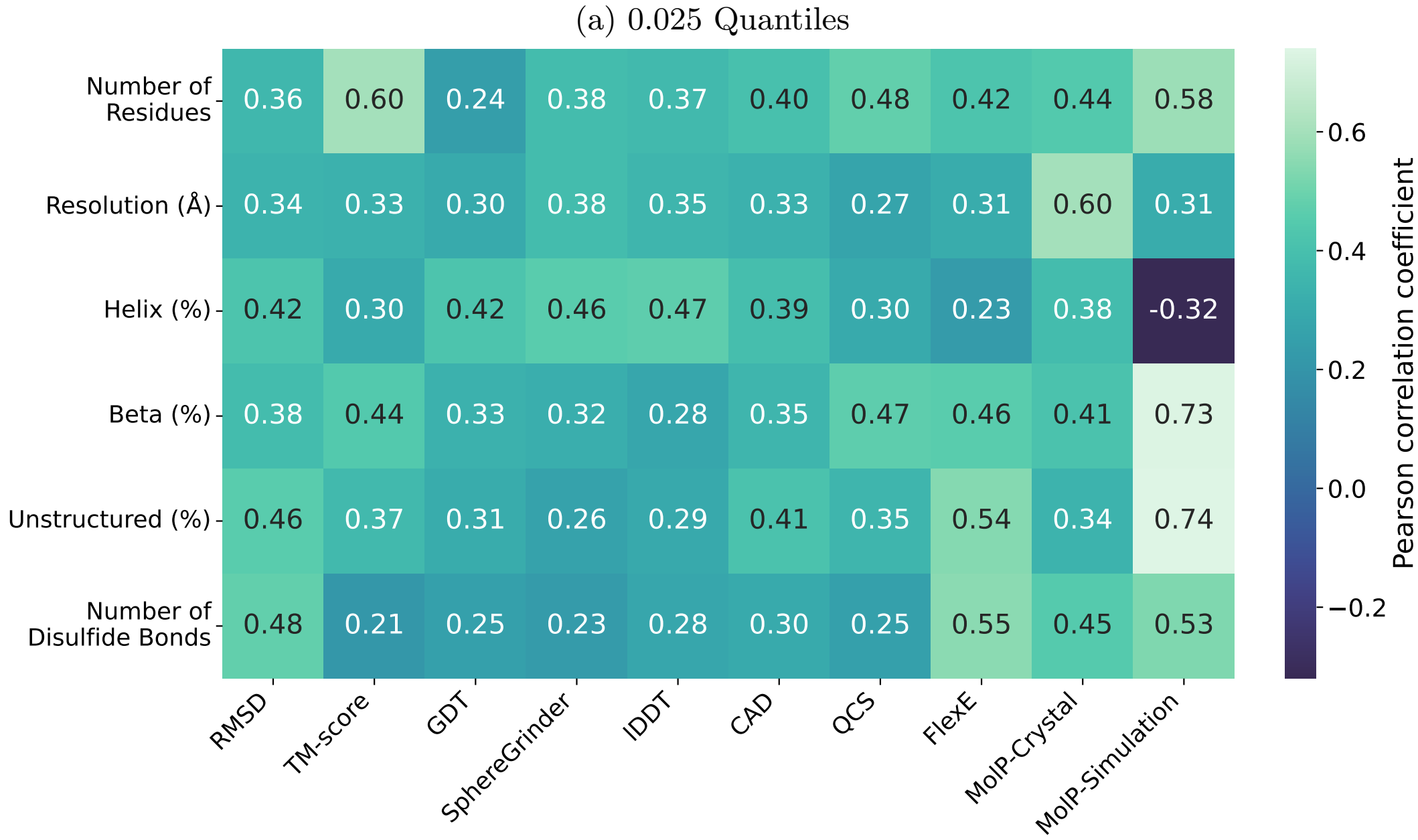

(b) 0.975 Quantiles

Figure S13: Estimation of the expected Pearson correlations between protein properties and scores. The following two steps were repeated 10 000 times: First, 25 physiological proteins were randomly selected from Set268. Second, the Pearson correlations between their respective scores (final snapshots of simulations conducted with C36m) and properties were computed. The resulting 0.025 and 0.975 quantiles are shown in (a) and (b), respectively.

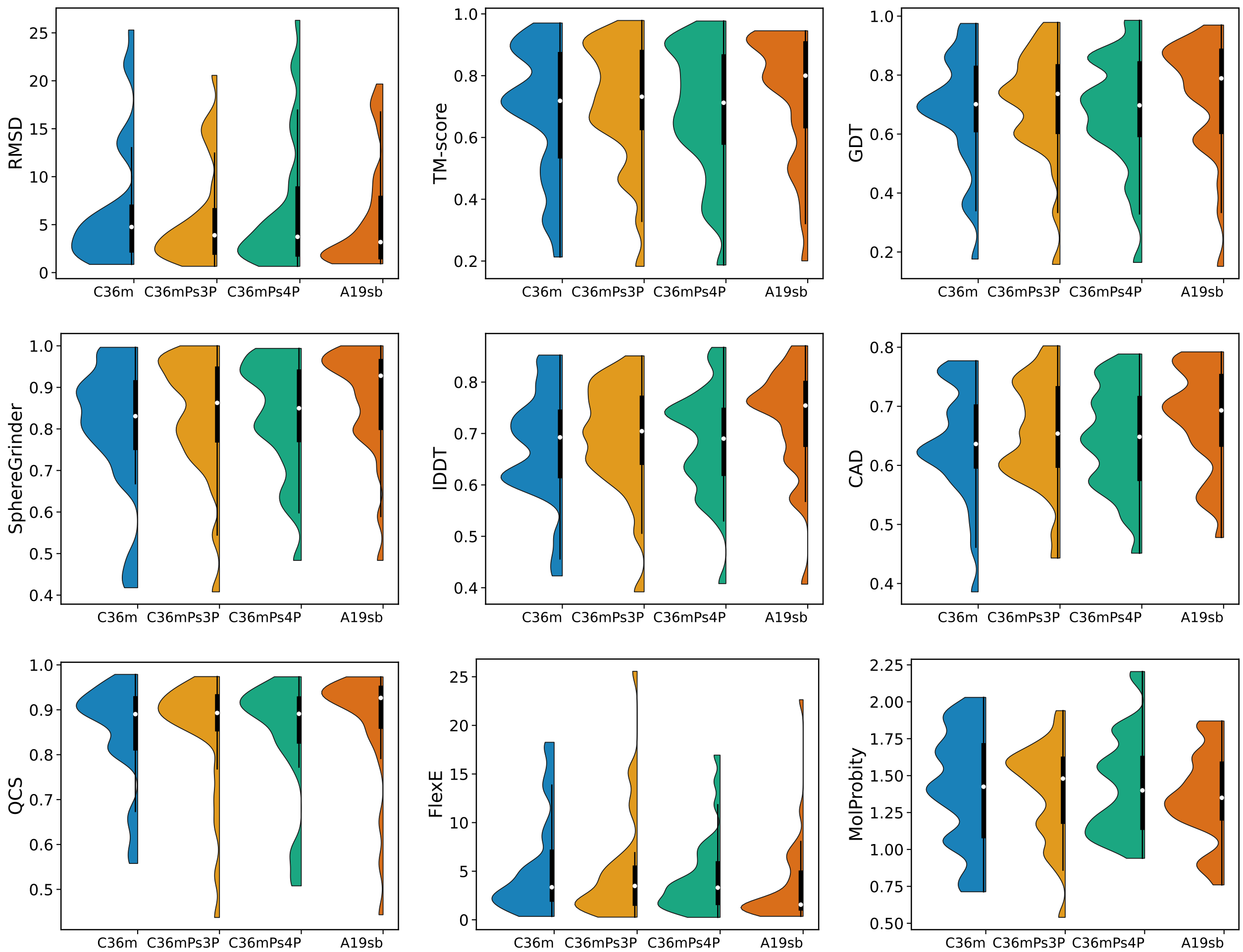

Figure S14: Score distributions for the simulation setups C36m, C36mPs3P, C36mPs4P, and A19sb based on Set31. For each violin plot, the median is depicted as a white point, the range from the first quartile to the third quartile is marked by a black box, and the lower and upper whiskers extend to the smallest and largest data points within 1.5 times the interquartile range from the first and third quartiles, respectively. RMSD values are given in Å and FlexE in kcal/mol/residue.

Table S5: Ranking of score medians across the simulation setups C36m, C36mPs3P, C36mPs4P, and A19sb based on Set31. Medians are ranked according to their similarity (quality for MolProbity) to the starting structure, from highest (rank 1) to lowest (rank 4).

Score	C36m	C36mPs3P	C36mPs4P	A19sb
RMSD	4	3	2	1
TM-score	3	2	4	1
GDT	3	2	4	1
SphereGrinder	4	2	3	1
lDDT	3	2	4	1
CAD	4	2	3	1
QCS	4	2	3	1
FlexE	3	4	2	1
MolProbity	3	4	2	1

Table S6: P-values for Friedman and post-hoc Wilcoxon signed-rank tests. For each score, the Friedman test [60] was initially conducted to test if any significant difference (Benjamini–Hochberg [61] corrected p-value ≤ 0.05) between the score distributions of the simulation setups C36m, C36mPs3P, C36mPs4P, and A19sb exists. If the Friedman test was significant for a specific score, post-hoc Wilcoxon signed-rank tests [62] were performed, one for each pair of simulation setups, to pinpoint which simulation setups are different from each other. The p-values were again adjusted via the Benjamini-Hochberg correction, and the significance level was 0.05. For the Wilcoxon signed-rank tests, only significant results are shown.

Score	Friedman adj. p-value	Setup Pair	Wilcoxon adj. p-value
RMSD	0.1069		
TM-score	0.0024	C36m–A19sb	0.0019
		C36mPs4P–A19sb	0.0041
		C36mPs3P–A19sb	0.0488
GDT	0.0070	C36m–A19sb	0.0102
		C36mPs4P–A19sb	0.0036
SphereGrinder	0.0015	C36m–A19sb	0.0027
		C36mPs4P–A19sb	0.0102
		C36mPs3P–A19sb	0.0107
lDDT	0.0006	C36m–A19sb	0.0019
		C36mPs4P–A19sb	0.0003
		C36mPs3P–A19sb	0.0027
CAD	0.0070	C36m–A19sb	0.0027
		C36mPs4P–A19sb	0.0019
		C36mPs3P–A19sb	0.0240
QCS	0.0087	C36m–A19sb	0.0321
		C36mPs4P–A19sb	0.0086
FlexE	0.0091	C36m–A19sb	0.0036
		C36mPs3P–A19sb	0.0186
MolProbity	0.7422		

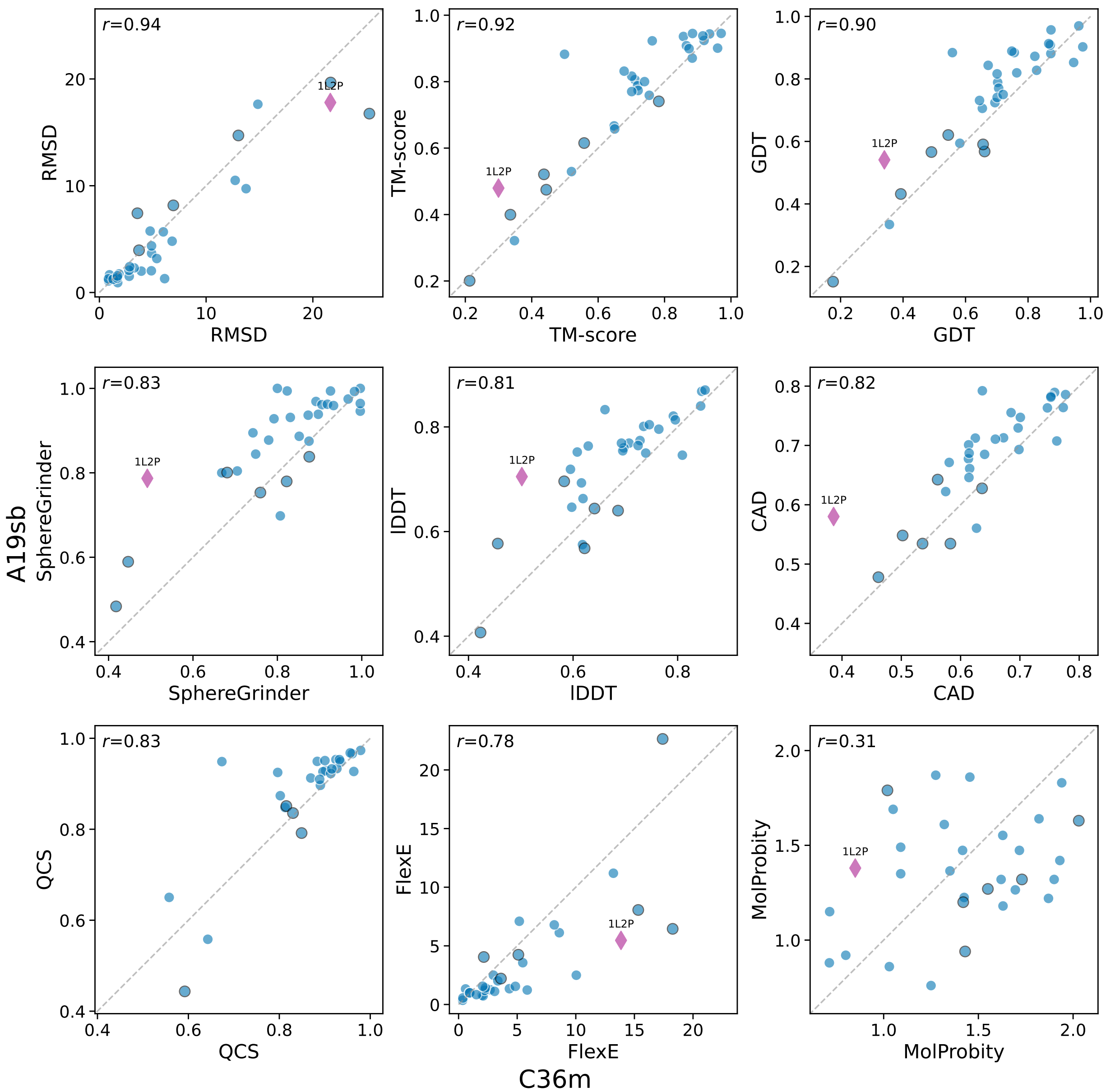

Figure S15: Score pair plots between the simulation setups C36m and A19sb based on Set31. Proteins are depicted as blue spheres, with non-physiological proteins being marked by black circles. The potential outlier 1L2P is highlighted by purple diamonds. Pearson correlation coefficients are displayed in the upper left corners, and the grey dashed lines represent the identity lines, indicating perfectly consistent scores. RMSD values are given in Å and FlexE in kcal/mol/residue.

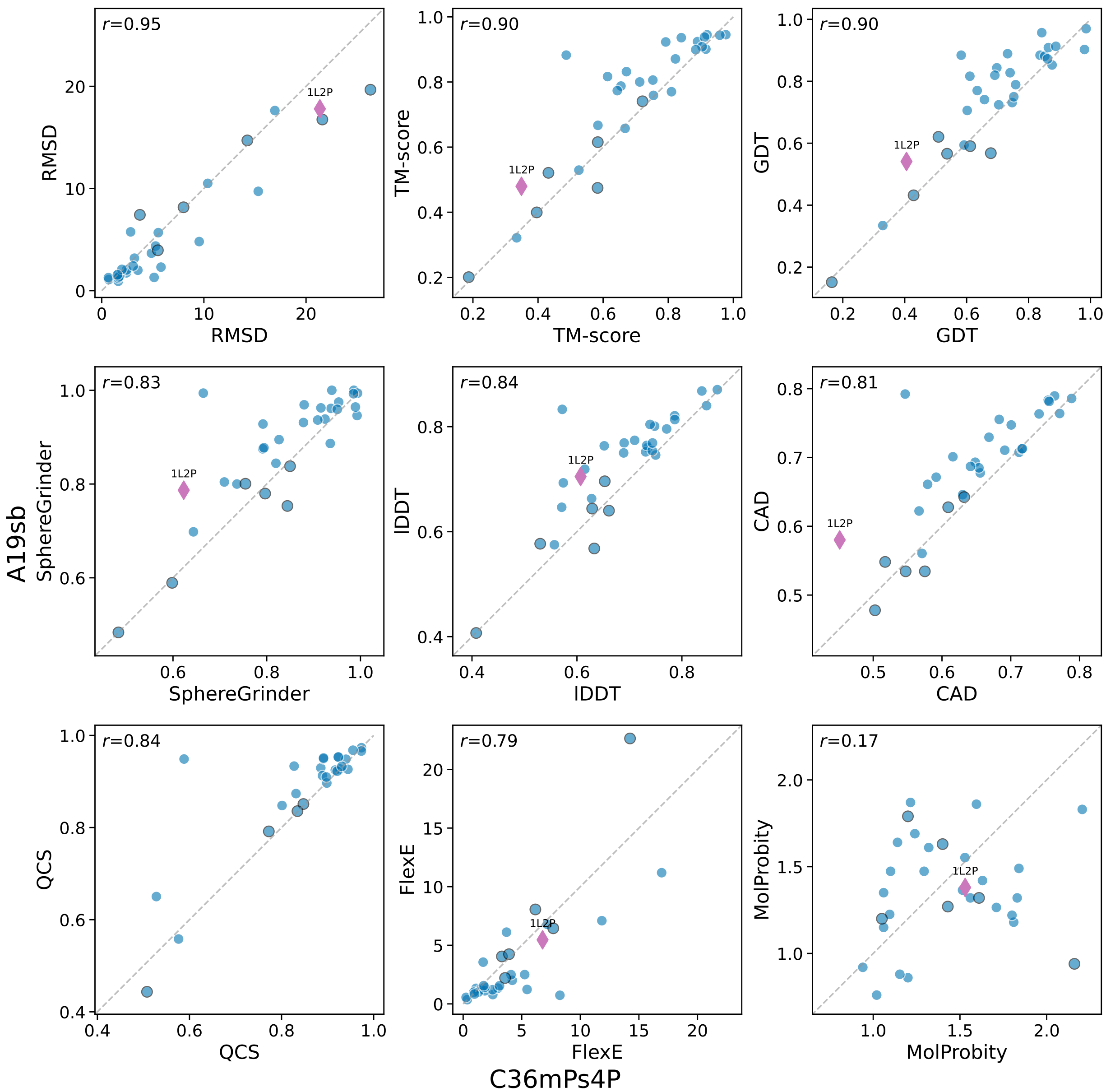

Figure S16: Score pair plots between the simulation setups C36mPs4P and A19sb based on Set31. Proteins are depicted as blue spheres, with non-physiological proteins being marked by black circles. The potential outlier 1L2P is highlighted by purple diamonds. Pearson correlation coefficients are displayed in the upper left corners, and the grey dashed lines represent the identity lines, indicating perfectly consistent scores. RMSD values are given in Å and FlexE in kcal/mol/residue.

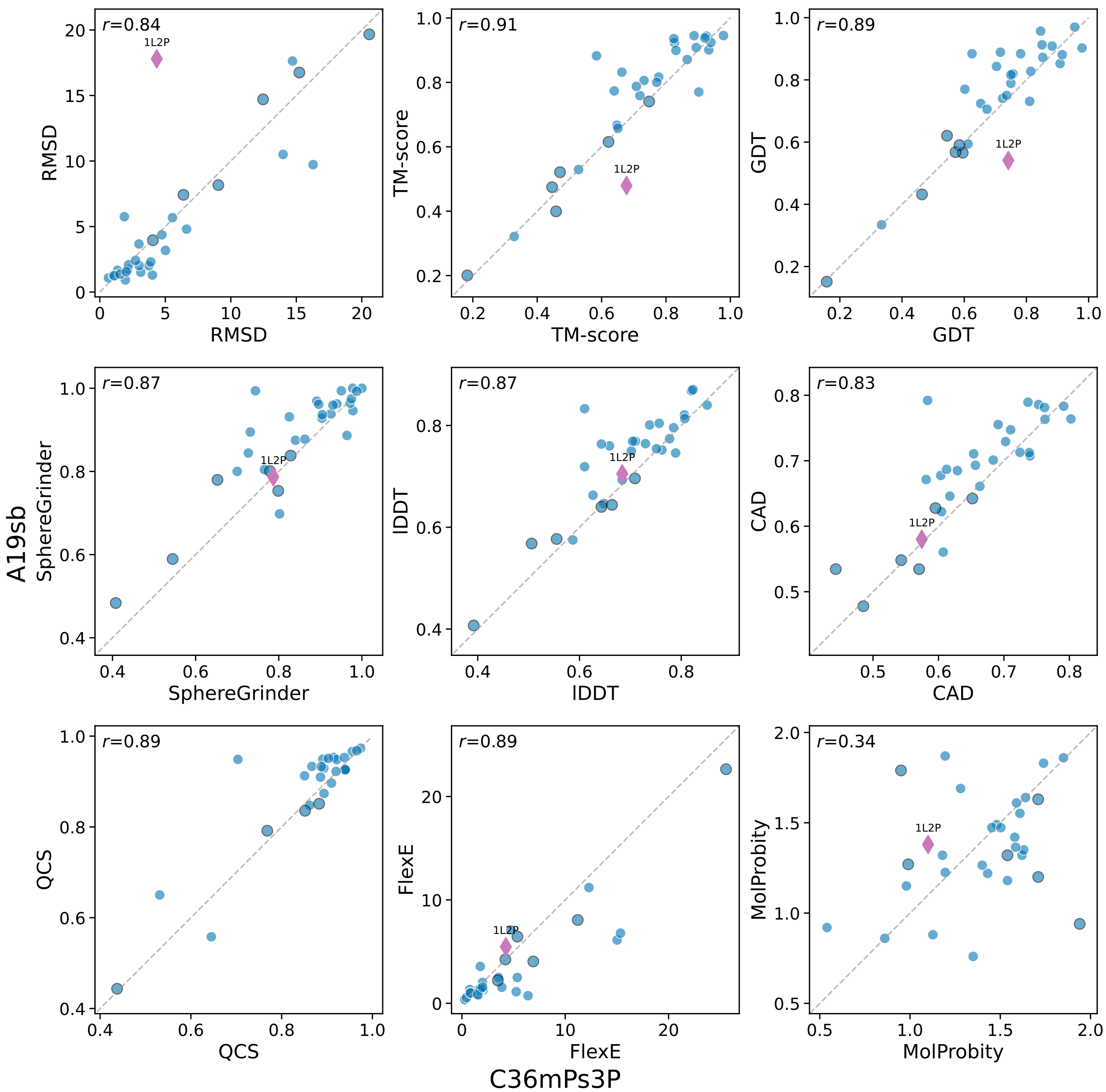

Figure S17: Score pair plots between the simulation setups C36mPs3P and A19sb based on Set31. Proteins are depicted as blue spheres, with non-physiological proteins being marked by black circles. The potential outlier 1L2P is highlighted by purple diamonds. Pearson correlation coefficients are displayed in the upper left corners, and the grey dashed lines represent the identity lines, indicating perfectly consistent scores. RMSD values are given in Å and FlexE in kcal/mol/residue.

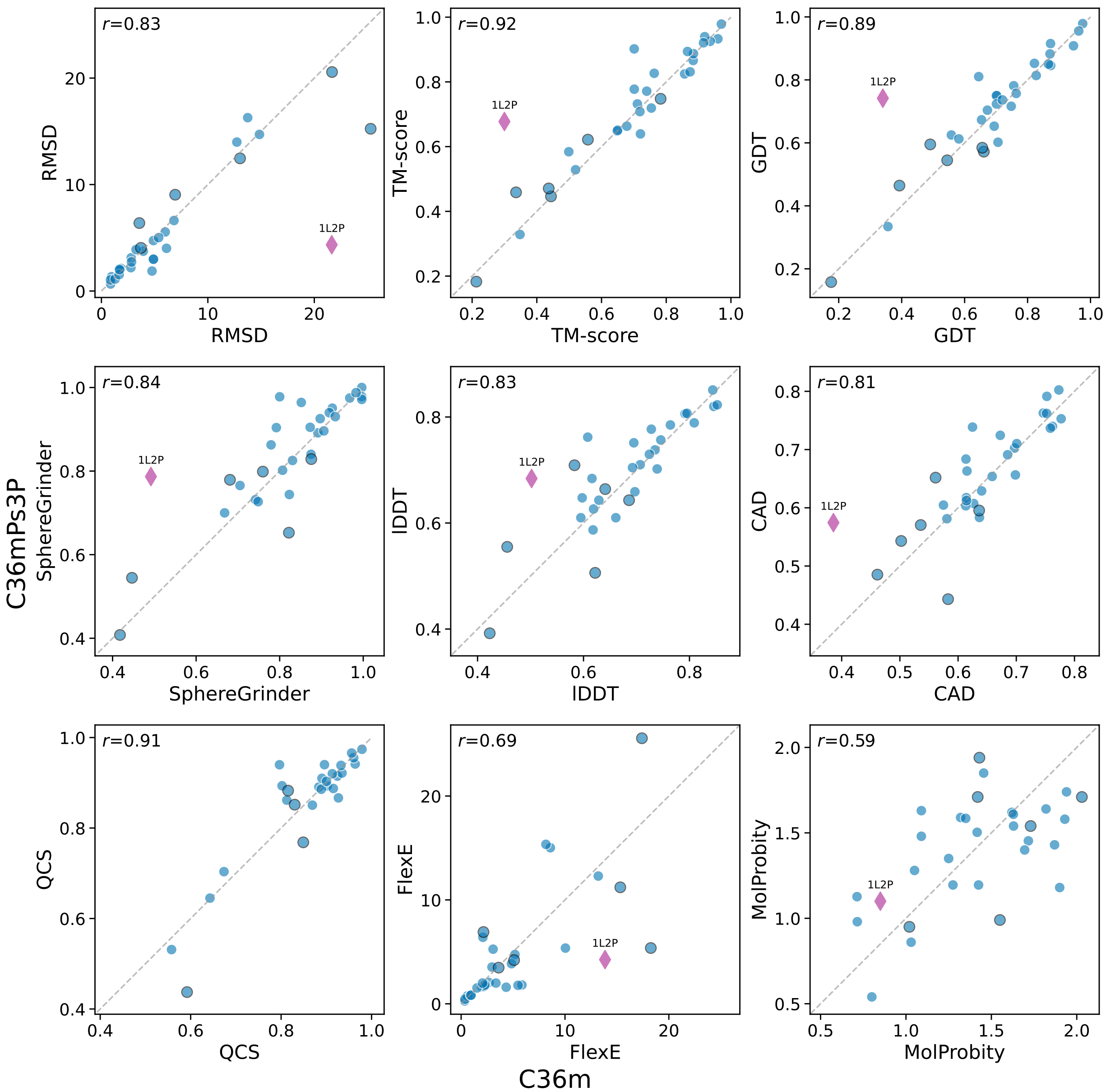

Figure S18: Score pair plots between the simulation setups C36m and C36mPs3P based on Set31. Proteins are depicted as blue spheres, with non-physical proteins being marked by black circles. The potential outlier 1L2P is highlighted by purple diamonds. Pearson correlation coefficients are displayed in the upper left corners, and the grey dashed lines represent the identity lines, indicating perfectly consistent scores. RMSD values are given in Å and FlexE in kcal/mol/residue.

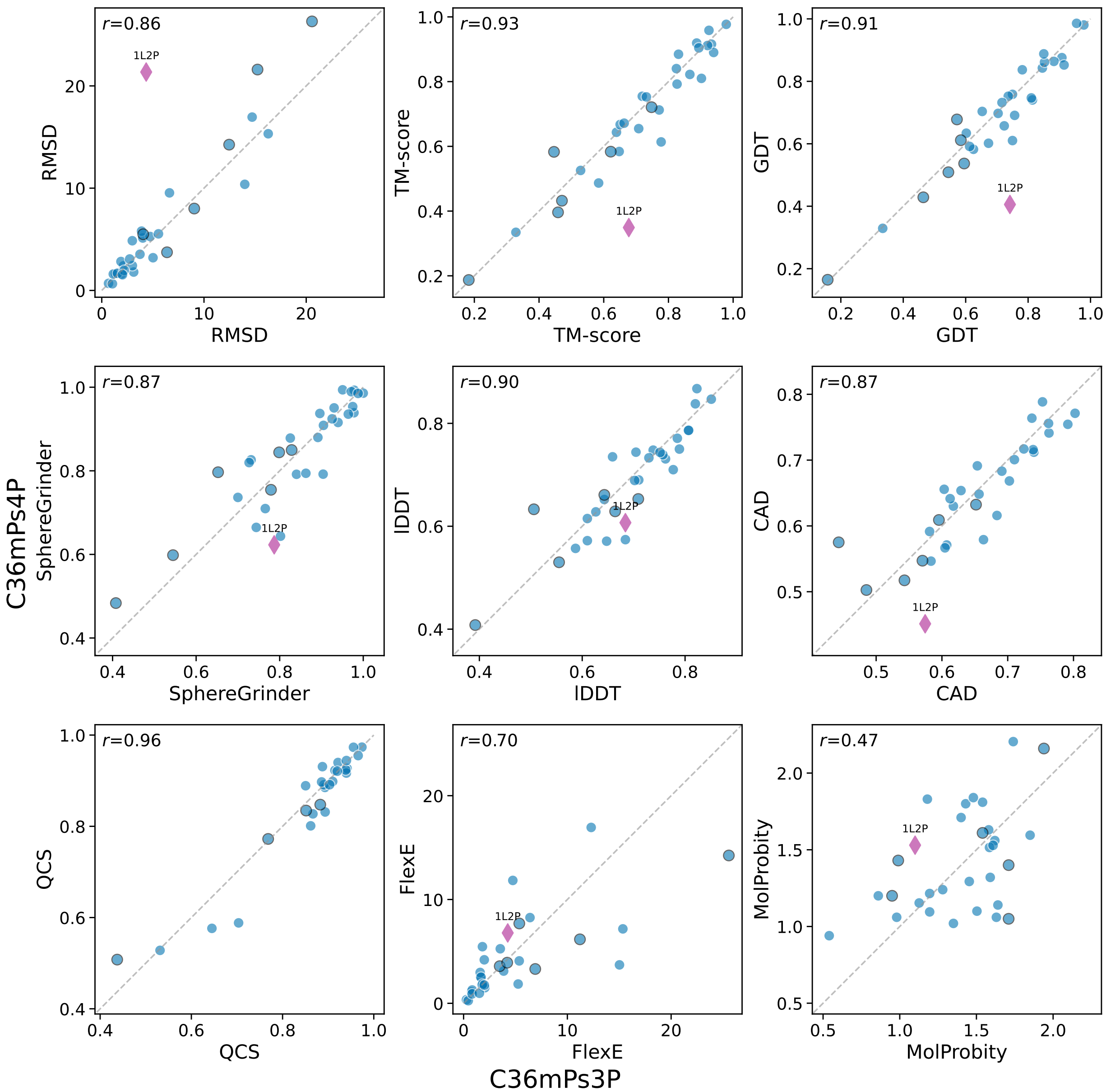

Figure S19: Score pair plots between the simulation setups C36mPs3P and C36mPs4P based on Set31. Proteins are depicted as blue spheres, with non-physical proteins being marked by black circles. The potential outlier 1L2P is highlighted by purple diamonds. Pearson correlation coefficients are displayed in the upper left corners, and the grey dashed lines represent the identity lines, indicating perfectly consistent scores. RMSD values are given in Å and FlexE in kcal/mol/residue.

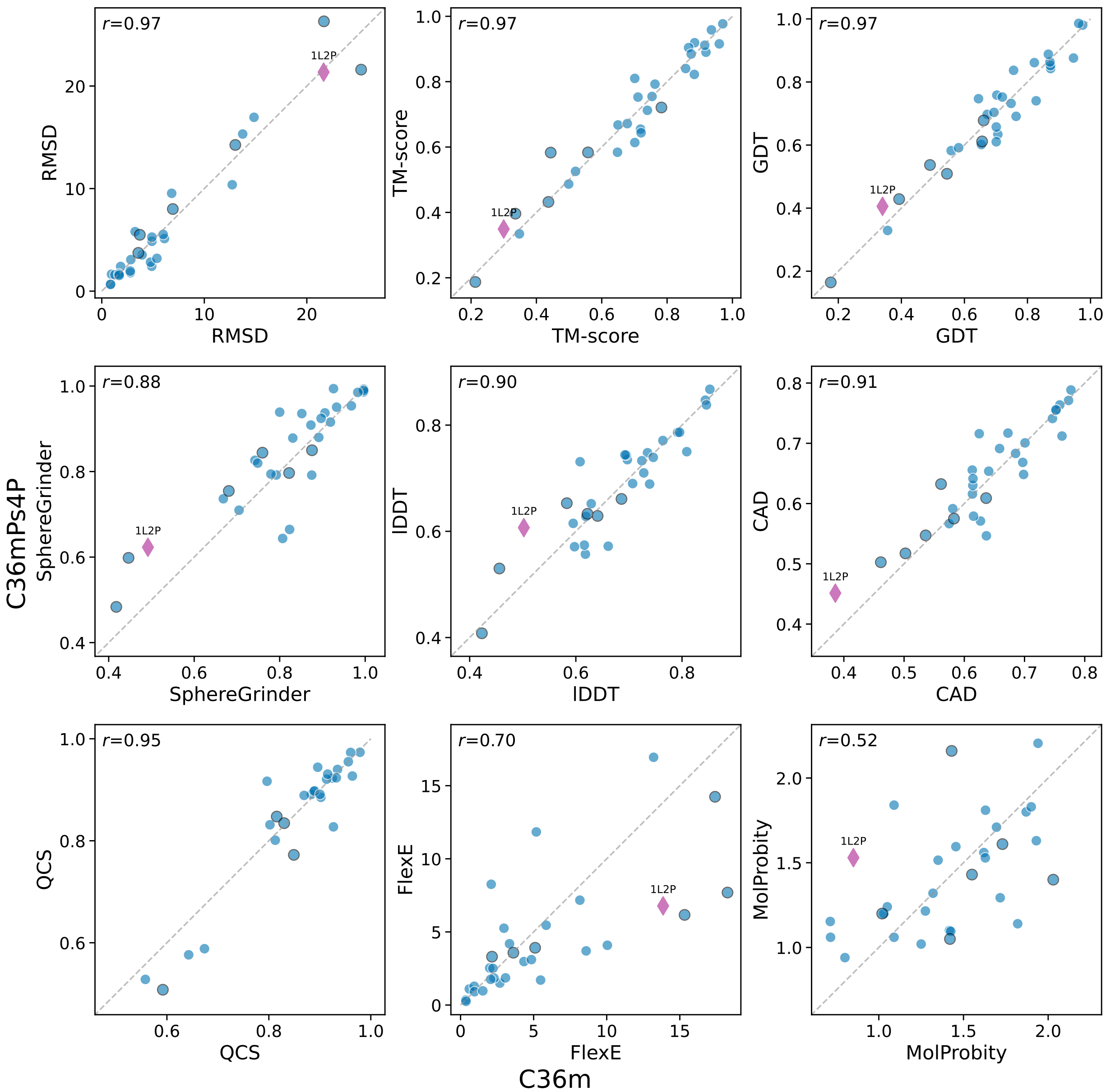

Figure S20: Score pair plots between the simulation setups C36m and C36mPs4P based on Set31. Proteins are depicted as blue spheres, with non-physical proteins being marked by black circles. The potential outlier 1L2P is highlighted by purple diamonds. Pearson correlation coefficients are displayed in the upper left corners, and the grey dashed lines represent the identity lines, indicating perfectly consistent scores. RMSD values are given in Å and FlexE in kcal/mol/residue.

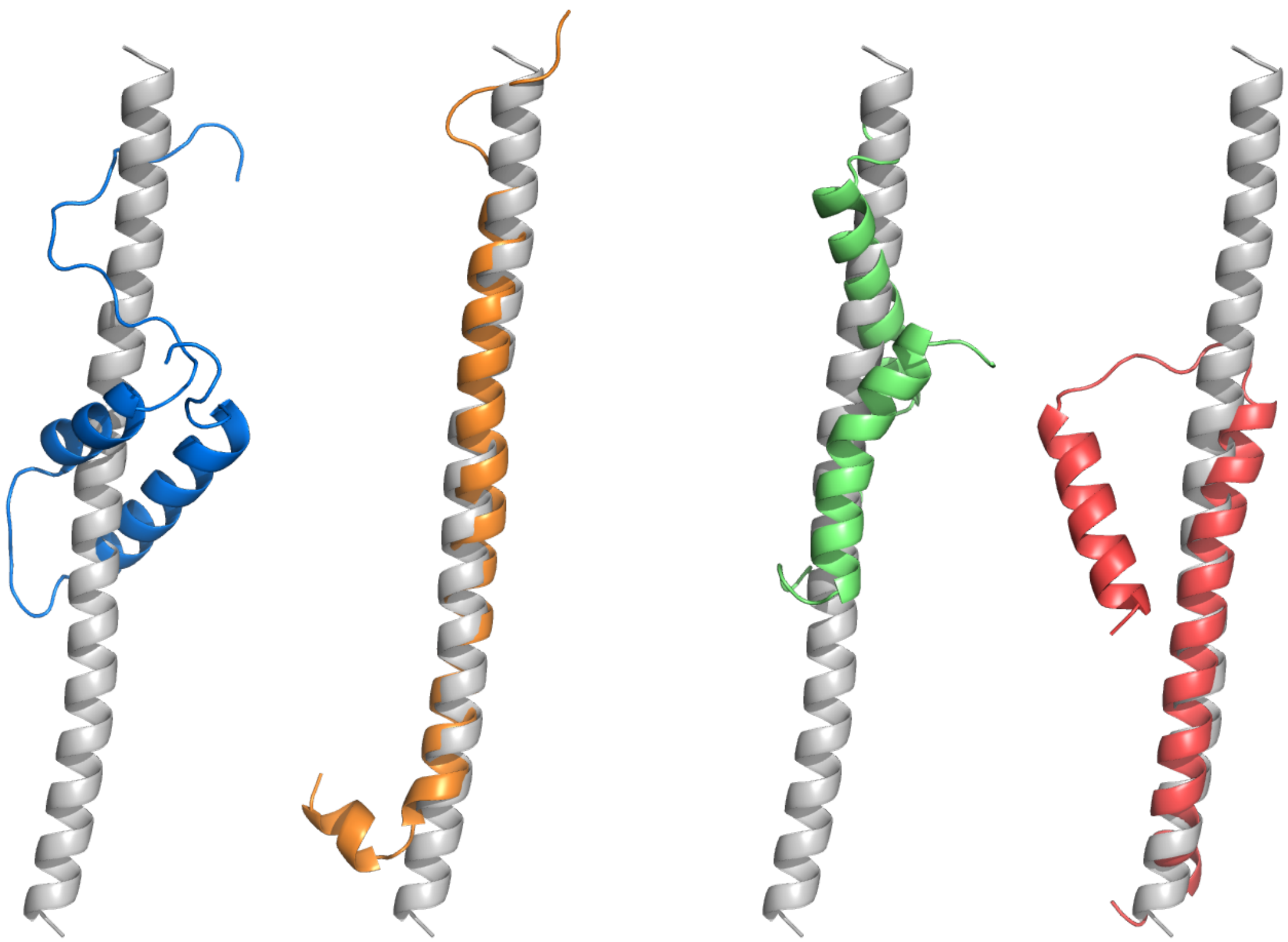

(a) C36m (b) C36mPs3P (c) C36mPs4P (d) A19sb

Figure S21: Final snapshots of the protein 1L2P for the four different simulation setups. The crystal structure is always shown in light grey, and the final simulation snapshots are displayed in different colors: (a) C36m (blue), (b) C36mPs3P (orange), (c) C36mPs4P (green), and (d) A19sb (red).

S8 Comparison of simulation setups based on MD trajectories and GDT

We computed GDT for snapshots extracted every 10 ns, with the initial 200 ns discarded as equilibration, yielding 80 score values per protein and simulation setup. For each protein and pair of simulation setups, we then quantified differences between the resulting score distributions using the EMD. In addition, we calculated the mean scores for each simulation setup and assessed their differences to investigate whether A19sb again yields systematically higher-similarity scores. Figure S22 compares score distributions of C36m and A19sb for the representative proteins 1AIL, 3UV9, 3GV3, and 135L, providing intuition for the interpretation of the EMDs and the differences in the means. Finally, for each simulation setup pair, we computed the average EMD and the average difference in the means over all proteins. The averaged EMDs were then normalized by the maximum value (normalization factor) observed for any simulation setup pair, yielding the normalized average EMDs.

Overall, the normalized average EMDs closely resemble the normalized EMDs obtained from the final snapshots (see Table S7), and the corresponding normalization factors are likewise similar. This indicates that the relative differences between simulation setups are largely preserved when the trajectories are considered.

Moreover, the largest average differences in the means were observed between A19sb and the CHARMM36m-based simulation setups, with the negative sign indicating higher mean scores for A19sb, consistent with the findings for the final simulation snapshots.

	Trajectories		Final snapshots
Simulation setup	Average difference in the means	Normalized average EMD (norm. factor = 0.055)	Normalized EMD (norm. factor = 0.058)
C36m–A19sb	−0.045	1.0	1.0
C36mPs4P–A19sb	−0.046	0.97	1.0
C36mPs3P–A19sb	−0.024	0.85	0.62
C36m–C36mPs3P	−0.021	0.66	0.55
C36mPs3P–C36mPs4P	0.021	0.69	0.50
C36m–C36mPs4P	0.001	0.51	0.32

Table S7: Comparison of score distributions based on final snapshots and trajectories across simulation setups. For each simulation setup pair (e.g., C36m–A19sb), the difference in means was computed as the mean of the first-listed setup minus the mean of the second-listed setup. Thus, for C36m–A19sb, an average difference in the means of −0.045 indicates that, on average across all proteins, the mean score of A19sb is 0.045 higher than that of C36m. Norm. factor: abbreviation for normalization factor.

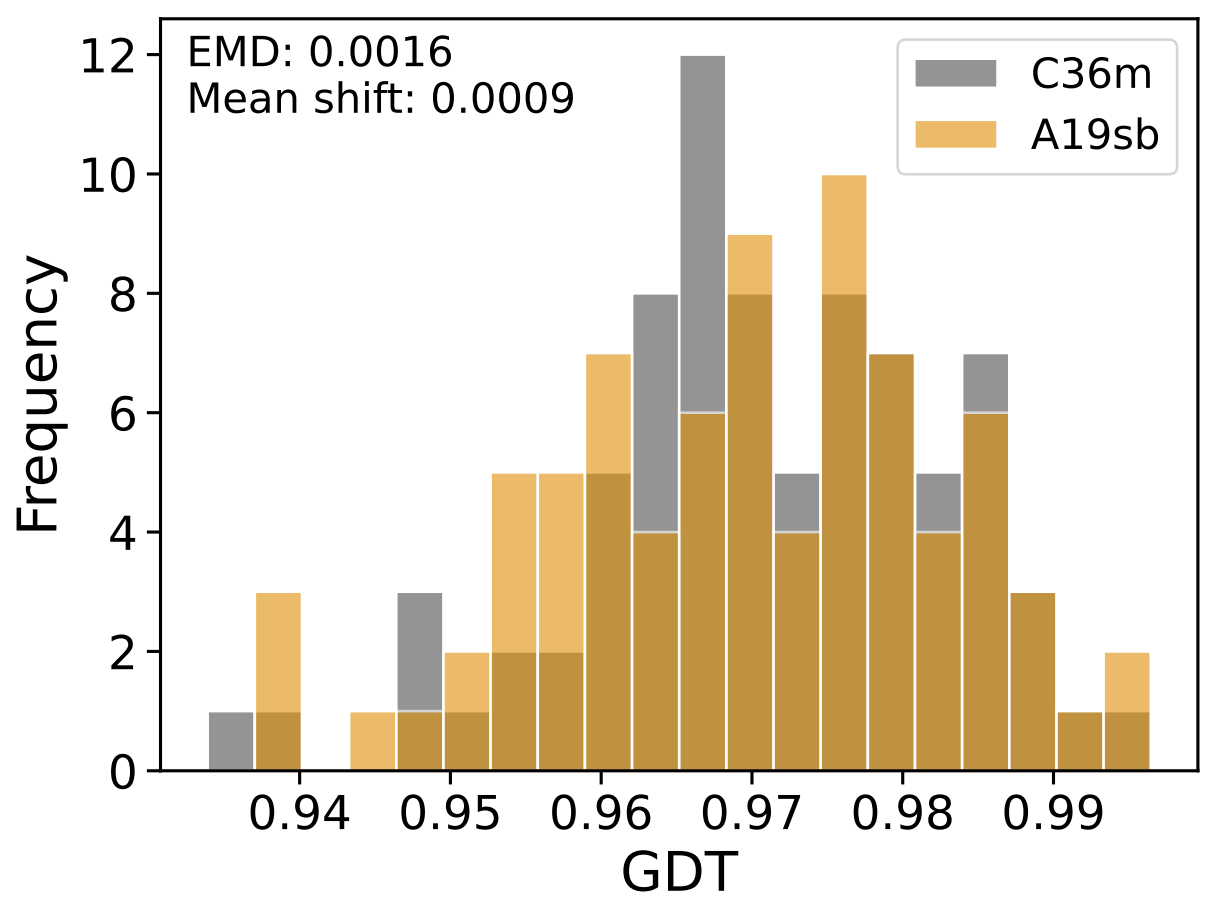

(a) 1AIL dimer

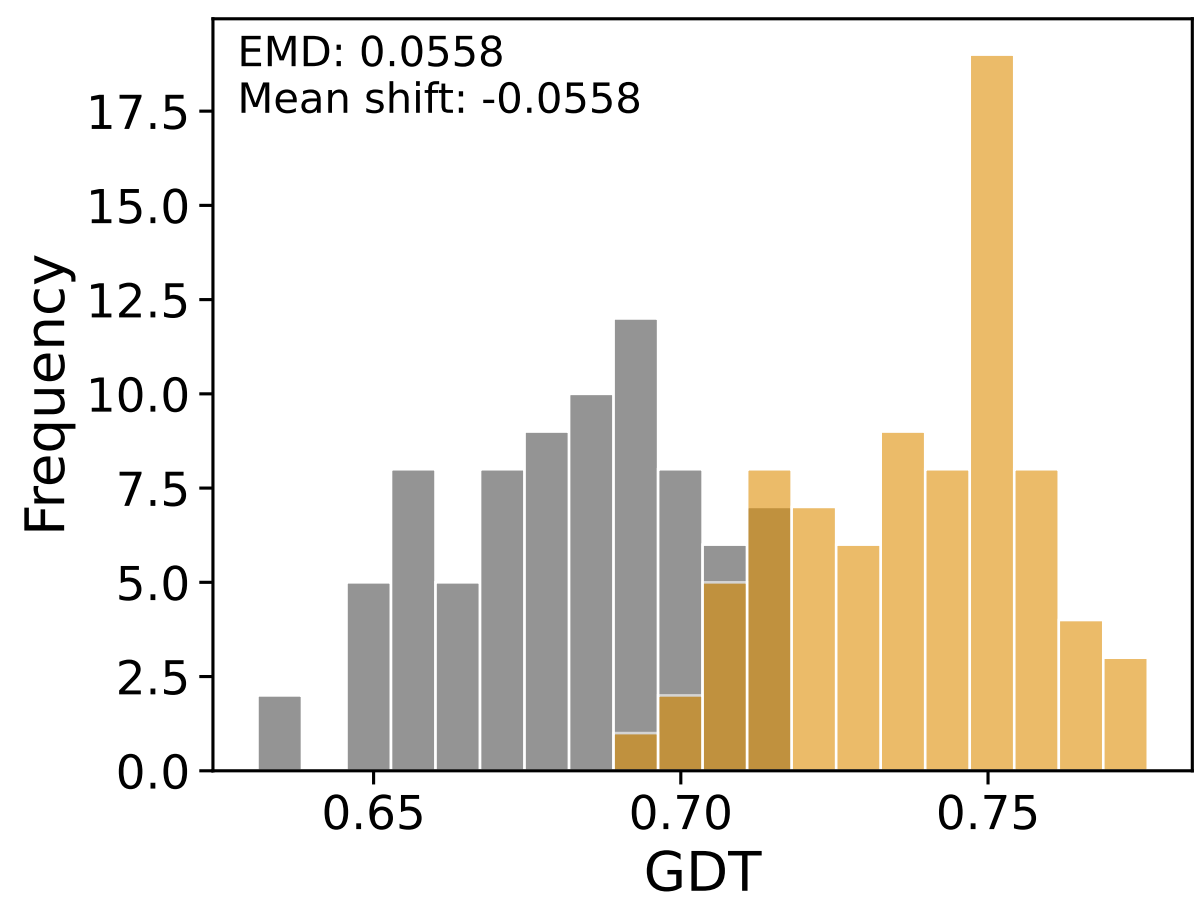

(b) 3UV9 monomer

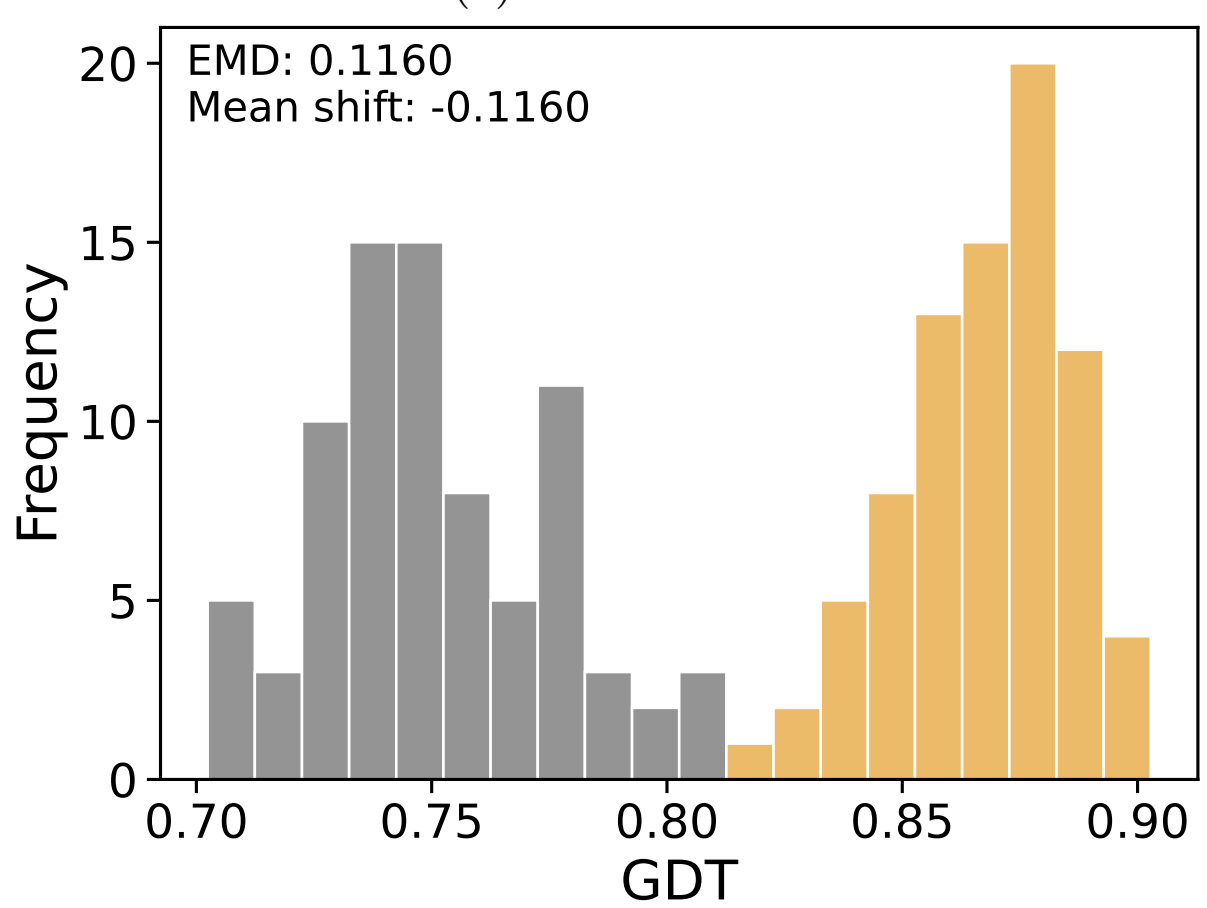

(c) 3GV3 dimer

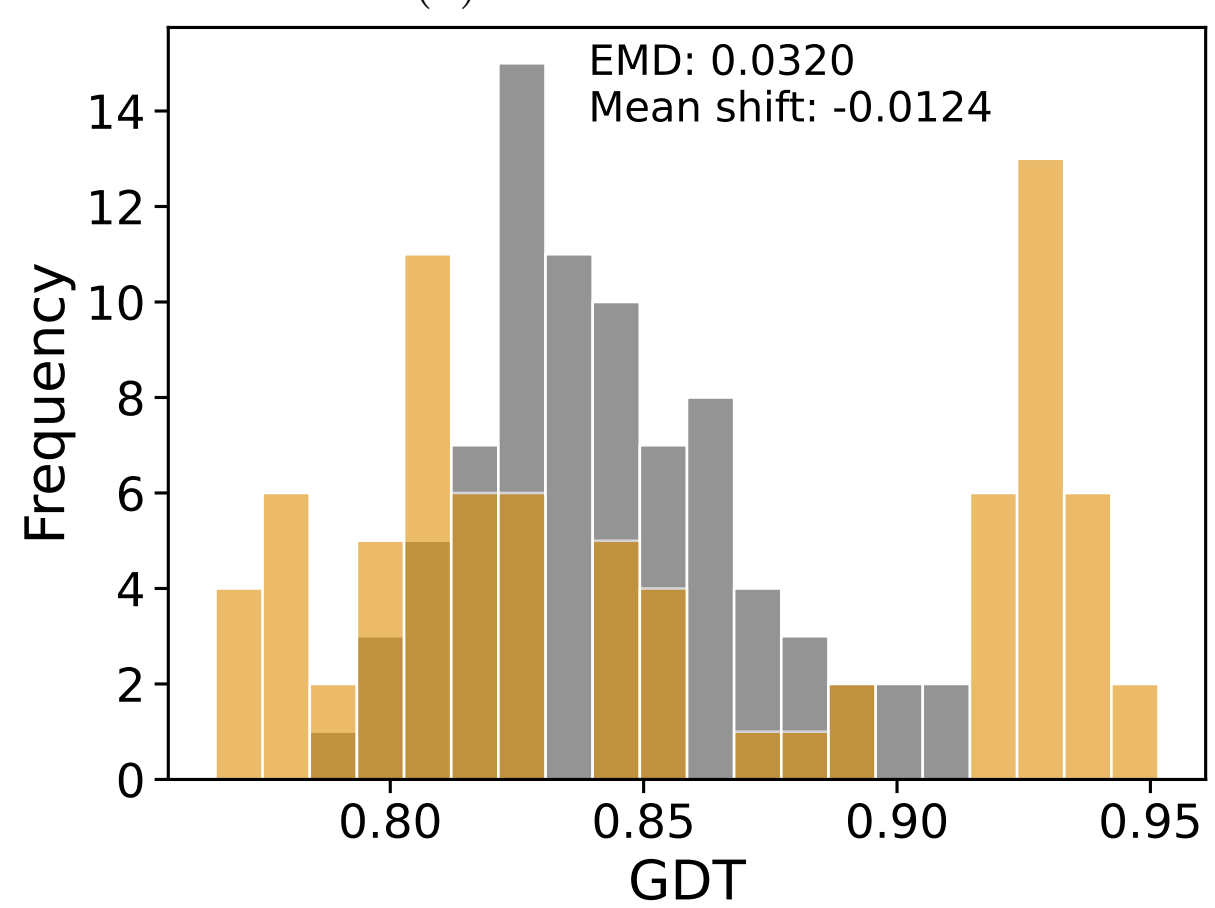

(d) 135L monomer

Figure S22: Trajectory score distributions of C36m and A19sb for representative proteins. The score distributions of C36m and A19sb are displayed in grey and orange, respectively, and the representative proteins are 1AIL (a), 3UV9 (b), 3GV3 (c), and 135L (d). The EMD and the difference between the means of each distribution (mean shift) are given in the upper region of each panel. A negative mean shift indicates a higher mean score for A19sb, and a positive mean shift a higher mean score for C36m. Notice that the EMD and the mean shift have the same value when the dominant difference between the distributions is a shift in location. This is the case for 3UV9 and 3GV3 but not for 1AIL and 135L.

References

(1) Abramson, J. et al. Accurate structure prediction of biomolecular interactions with AlphaFold 3. *Nature* **2024**, *630*, 493–500, DOI: 10.1038/s41586-024-07487-w.

(2) Berman, H. M.; Westbrook, J.; Feng, Z.; Gilliland, G.; Bhat, T. N.; Weissig, H.; Shindyalov, I. N.; Bourne, P. E. The Protein Data Bank. *Nucleic Acids Res.* **2000**, *28*, 235–242, DOI: 10.1093/nar/28.1.235.

(3) Kabsch, W. A solution for the best rotation to relate two sets of vectors. *Acta Crystallogr. A* **1976**, *32*, 922–923, DOI: 10.1107/S0567739476001873.

(4) Zhang, Y.; Skolnick, J. Scoring function for automated assessment of protein structure template quality. *Proteins: Struct., Funct., Bioinf.* **2004**, *57*, 702–710, DOI: 10.1002/prot.20264.

(5) Zemla, A.; Venclovas, Č.; Moult, J.; Fidelis, K. Processing and analysis of CASP3 protein structure predictions. *Proteins: Struct., Funct., Bioinf.* **1999**, *37*, 22–29, DOI: 10.1002/(SICI)1097-0134(1999)37:3+<22::AID-PROT5>3.0.CO;2-W.

(6) Zemla, A.; Venclovas, Č.; Moult, J.; Fidelis, K. Processing and evaluation of predictions in CASP4. *Proteins: Struct., Funct., Bioinf.* **2001**, *45*, 13–21, DOI: 10.1002/prot.10052.

(7) Mariani, V.; Biasini, M.; Barbato, A.; Schwede, T. lDDT: a local superposition-free score for comparing protein structures and models using distance difference tests. *Bioinformatics* **2013**, *29*, 2722–2728, DOI: 10.1093/bioinformatics/btt473.

(8) Biasini, M.; Schmidt, T.; Bienert, S.; Mariani, V.; Studer, G.; Haas, J.; Johner, N.; Schenk, A. D.; Philippsen, A.; Schwede, T. *OpenStructure*: an integrated software framework for computational structural biology. *Acta Crystallogr. D* **2013**, *69*, 701–709, DOI: 10.1107/S0907444913007051.

(9) Antczak, P. L. M.; Ratajczak, T.; Lukasiak, P.; Blazewicz, J. In *2015 IEEE International Conference on Bioinformatics and Biomedicine (BIBM)*, 2015, pp 665–668, DOI: 10.1109/BIBM.2015.7359765.

(10) Olechnovič, K.; Monastyrskyy, B.; Kryshtafovych, A.; Venclovas, Č. Comparative analysis of methods for evaluation of protein models against native structures. *Bioinformatics* **2019**, *35*, 937–944, DOI: 10.1093/bioinformatics/bty760.

(11) Kryshtafovych, A.; Monastyrskyy, B.; Fidelis, K. CASP prediction center infrastructure and evaluation measures in CASP10 and CASP ROLL. *Proteins: Struct., Funct., Bioinf.* **2014**, *82*, 7–13, DOI: 10.1002/prot.24399.

(12) Cong, Q.; Kinch, L. N.; Pei, J.; Shi, S.; Grishin, V. N.; Li, W.; Grishin, N. V. An automatic method for CASP9 free modeling structure prediction assessment. *Bioinformatics* **2011**, *27*, 3371–3378, DOI: 10.1093/bioinformatics/btr572.

(13) Majumdar, I.; Krishna, S. S.; Grishin, N. V. PALSSE: A program to delineate linear secondary structural elements from protein structures. *BMC Bioinform.* **2005**, *6*, DOI: 10.1186/1471-2105-6-202.

(14) Olechnovič, K.; Kulberkytė, E.; Venclovas, Č. CAD-score: A new contact area difference-based function for evaluation of protein structural models. *Proteins: Struct., Funct., Bioinf.* **2013**, *81*, 149–162, DOI: https://doi.org/10.1002/prot.24172.

(15) Olechnovič, K.; Venclovas, Č. Voronota: A fast and reliable tool for computing the vertices of the Voronoi diagram of atomic balls. *J. Comput. Chem.* **2014**, *35*, 672–681, DOI: 10.1002/jcc.23538.

(16) Perez, A.; Yang, Z.; Bahar, I.; Dill, K. A.; MacCallum, J. L. FlexE: using elastic network models to compare models of protein structure. *J. Chem. Theory Comput.* **2012**, *8*, 3985–3991, DOI: 10.1021/ct300148f.

(17) Williams, C. J. et al. MolProbity: More and better reference data for improved all-atom structure validation. *Protein Sci.* **2018**, *27*, 293–315, DOI: 10.1002/pro.3330.

(18) Chen, V. B.; Arendall III, W. B.; Headd, J. J.; Keedy, D. A.; Immormino, R. M.; Kapral, G. J.; Murray, L. W.; Richardson, J. S.; Richardson, D. C. *MolProbity*: all-atom structure validation for macromolecular crystallography. *Acta Crystallogr. D* **2010**, *66*, 12–21, DOI: 10.1107/S0907444909042073.

(19) Hooft, R. W.; Sander, C.; Vriend, G. Objectively judging the quality of a protein structure from a Ramachandran plot. *Bioinformatics* **1997**, *13*, 425–430, DOI: 10.1093/bioinformatics/13.4.425.

(20) Consortium, T. U. UniProt: the Universal Protein Knowledgebase in 2025. *Nucleic Acids Res.* **2024**, *53*, D609–D617, DOI: 10.1093/nar/gkae1010.

(21) Touw, W. G.; Baakman, C.; Black, J.; te Beek, T. A. H.; Krieger, E.; Joosten, R. P.; Vriend, G. A series of PDB-related databanks for everyday needs. *Nucleic Acids Res.* **2014**, *43*, D364–D368, DOI: 10.1093/nar/gku1028.

(22) Kabsch, W.; Sander, C. Dictionary of protein secondary structure: Pattern recognition of hydrogen-bonded and geometrical features. *Biopolymers* **1983**, *22*, 2577–2637, DOI: https://doi.org/10.1002/bip.360221211.

(23) Bekker, H.; Berendsen, H.; Dijkstra, E.; Achterop, S.; Vondrumen, R.; Vanderspoel, D.; Sijbers, A.; Keegstra, H.; Renardus, M. In *4th international conference on computational physics (PC 92)*, 1993, pp 252–256.

(24) Abraham, M. J.; Murtola, T.; Schulz, R.; Páll, S.; Smith, J. C.; Hess, B.; Lindahl, E. GROMACS: High performance molecular simulations through multi-level parallelism from laptops to supercomputers. *SoftwareX* **2015**, *1*, 19–25, DOI: 10.1016/j.softx.2015.06.001.

(25) Abel, R.; Young, T.; Farid, R.; Berne, B. J.; Friesner, R. A. Role of the Active-Site Solvent in the Thermodynamics of Factor Xa Ligand Binding. *J. Am. Chem. Soc.* **2008**, *130*, 2817–2831, DOI: 10.1021/ja0771033.

(26) Šali, A.; Blundell, T. L. Comparative Protein Modelling by Satisfaction of Spatial Restraints. *Journal of Molecular Biology* **1993**, *234*, 779–815, DOI: https://doi.org/10.1006/jmbi.1993.1626.

(27) Hess, B. P-LINCS: A parallel linear constraint solver for molecular simulation. *J. Chem. Theory Comput.* **2008**, *4*, 116–122, DOI: 10.1021/ct700200b.

(28) Páll, S.; Hess, B. A flexible algorithm for calculating pair interactions on SIMD architectures. *Comput. Phys. Commun.* **2013**, *184*, 2641–2650, DOI: 10.1016/j.cpc.2013.06.003.

(29) Darden, T.; York, D.; Pedersen, L. Particle mesh Ewald: An $N \log(N)$ method for Ewald sums in large systems. *J. Chem. Phys.* **1993**, *98*, 10089–10092, DOI: 10.1063/1.464397.

(30) Berendsen, H. J.; Postma, J. v.; Van Gunsteren, W. F.; DiNola, A.; Haak, J. R. Molecular dynamics with coupling to an external bath. *The Journal of chemical physics* **1984**, *81*, 3684–3690, DOI: 10.1063/1.448118.

(31) Evans, D. J.; Holian, B. L. The nose–hoover thermostat. *J. Chem. Phys.* **1985**, *83*, 4069–4074, DOI: 10.1063/1.449071.

(32) Bernetti, M.; Bussi, G. Pressure control using stochastic cell rescaling. *J. Chem. Phys.* **2020**, *153*, 114107, DOI: 10.1063/5.0020514.

(33) Shirts, M. R.; Mobley, D. L.; Chodera, J. D.; Pande, V. S. Accurate and Efficient Corrections for Missing Dispersion Interactions in Molecular Simulations. *The Journal of Physical Chemistry B* **2007**, *111*, 13052–13063, DOI: 10.1021/jp0735987.

(34) Brooks, B. R.; Bruccoleri, R. E.; Olafson, B. D.; States, D. J.; Swaminathan, S.; Karplus, M. CHARMM: A program for macromolecular energy, minimization, and dynamics calculations. *J. Comput. Chem.* **1983**, *4*, 187–217, DOI: 10.1002/jcc.540040211.

(35) Brooks, B. R. et al. CHARMM: The biomolecular simulation program. *J. Comput. Chem.* **2009**, *30*, 1545–1614, DOI: https://doi.org/10.1002/jcc.21287.

(36) Best, R. B.; Zhu, X.; Shim, J.; Lopes, P. E. M.; Mittal, J.; Feig, M.; MacKerell, A. D. J. Optimization of the Additive CHARMM All-Atom Protein Force Field Targeting Improved Sampling of the Backbone ϕ, ψ and Side-Chain χ_1 and χ_2 Dihedral Angles. *J. Chem. Theory Comput.* **2012**, *8*, 3257–3273, DOI: 10.1021/ct300400x.

(37) Huang, J.; Rauscher, S.; Nawrocki, G.; Ran, T.; Feig, M.; De Groot, B. L.; Grubmüller, H.; MacKerell, A. D. CHARMM36m: an improved force field for folded and intrinsically disordered proteins. *Nat. Methods* **2017**, *14*, 71–73, DOI: 10.1038/nmeth.4067.

(38) Perilla, J. R.; Schulten, K. Physical properties of the HIV-1 capsid from all-atom molecular dynamics simulations. *Nat. Commun.* **2017**, *8*, 15959, DOI: 10.1038/ncomms15959.

(39) Rodríguez-Espigares, I.; Torrens-Fontanals, M.; Tiemann, J. K.; Aranda-García, D.; Ramírez-Anguita, J. M.; Stepniewski, T. M.; Worp, N.; Varela-Rial, A.; Morales-Pastor, A.; Medel-Lacruz, B., et al. GPCRmd uncovers the dynamics of the 3D-GPCRome. *Nat. Methods* **2020**, *17*, 777–787, DOI: 10.1038/s41592-020-0884-y.

(40) Johnson, A. G.; Mayer, M. L.; Schaefer, S. L.; McNamara-Bordewick, N. K.; Hummer, G.; Kranzusch, P. J. Structure and assembly of a bacterial gasdermin pore. *Nature* **2024**, *628*, 657–663, DOI: 10.1038/s41586-024-07216-3.

(41) Pluhackova, K.; Schittny, V.; Bürkner, P.-C.; Siligan, C.; Horner, A. Multiple pore lining residues modulate water permeability of GlpF. *Protein Sci.* **2022**, *31*, e4431, DOI: 10.1002/pro.4431.

(42) Lee, J. et al. CHARMM-GUI Input Generator for NAMD, GROMACS, AMBER, OpenMM, and CHARMM/OpenMM Simulations Using the CHARMM36 Additive Force Field. *J. Chem. Theory Comput.* **2016**, *12*, 405–413, DOI: 10.1021/acs.jctc.5b00935.

(43) Love, O.; Galindo-Murillo, R.; Zgarbová, M.; Šponer, J.; Jurečka, P.; Cheatham, T. E. I. Assessing the Current State of Amber Force Field Modifications for DNA-2023 Edition. *J. Chem. Theory Comput.* **2023**, *19*, 4299–4307, DOI: 10.1021/acs.jctc.3c00233.

(44) Janowski, P. A.; Liu, C.; Deckman, J.; Case, D. A. Molecular dynamics simulation of triclinic lysozyme in a crystal lattice. *Protein Sci.* **2016**, *25*, 87–102, DOI: 10.1002/pro.2713.

(45) Tian, C.; Kasavajhala, K.; Belfon, K. A. A.; Raguette, L.; Huang, H.; Migues, A. N.; Bickel, J.; Wang, Y.; Pincay, J.; Wu, Q.; Simmerling, C. ff19SB: Amino-Acid-Specific Protein Backbone Parameters Trained against Quantum Mechanics Energy Surfaces in Solution. *J. Chem. Theory Comput.* **2020**, *16*, 528–552, DOI: 10.1021/acs.jctc.9b00591.

(46) Maier, J. A.; Martinez, C.; Kasavajhala, K.; Wickstrom, L.; Haus er, K. E.; Simmerling, C. ff14SB: Improving the Accuracy of Protein Side Chain and Backbone Parameters from ff99SB. *J. Chem. Theory Comput.* **2015**, *11*, 3696–3713, DOI: 10.1021/acs.jctc.5b00255.

(47) Team, G. D. Molecular dynamics parameters, https://manual.gromacs.org/current/user-guide/mdp-options.html, Accessed: 2025-08-15.

(48) Whitmore, L. M.; Ramezani, Y.; Sharma, S.; Shirts, M. R. Force Switching and Potential Shifting Lead to Significant Cutoff Dependence in Alchemical Free Energies. *J. Chem. Theory Comput.* **2025**, *21*, 7967–7976, DOI: 10.1021/acs.jctc.5c00646.

(49) Klauda, J. B.; Venable, R. M.; Freites, J. A.; O'Connor, J. W.; Tobias, D. J.; Mondragon-Ramirez, C.; Vorobyov, I.; MacKerell, A. D. J.; Pastor, R. W. Update of the CHARMM All-Atom Additive Force Field for Lipids: Validation on Six Lipid Types. *J. Phys. Chem. B* **2010**, *114*, 7830–7843, DOI: 10.1021/jp101759q.

(50) Piggot, T. J.; Piñeiro, Á.; Khalid, S. Molecular Dynamics Simulations of Phosphatidylcholine Membranes: A Comparative Force Field Study. *J. Chem. Theory Comput.* **2012**, *8*, 4593–4609, DOI: 10.1021/ct3003157.

(51) MacKerell, A. D. J. et al. All-Atom Empirical Potential for Molecular Modeling and Dynamics Studies of Proteins. *J. Phys. Chem. B* **1998**, *102*, 3586–3616, DOI: 10.1021/jp973084f.

(52) Croitoru, A.; Park, S.-J.; Kumar, A.; Lee, J.; Im, W.; MacKerell, A. D. J.; Aleksandrov, A. Additive CHARMM36 Force Field for Nonstandard Amino Acids. *J. Chem. Theory Comput.* **2021**, *17*, 3554–3570, DOI: 10.1021/acs.jctc.1c00254.

(53) Bjelkmar, P.; Larsson, P.; Cuendet, M. A.; Hess, B.; Lindahl, E. Implementation of the CHARMM Force Field in GROMACS: Analysis of Protein Stability Effects from Correction Maps, Virtual Interaction Sites, and Water Models. *J. Chem. Theory Comput.* **2010**, *6*, 459–466, DOI: 10.1021/ct900549r.

(54) Chaplin, M. Water models, https://water.lsbu.ac.uk/water/water_models.html, Accessed: 2025-08-15, 2021.

(55) Lautner, L.; Pluhackova, K.; Barth, N. K.; Seydel, T.; Lohstroh, W.; Böckmann, R. A.; Unruh, T. Dynamic processes in biological membrane mimics revealed by quasielastic neutron scattering. *Chem. Phys. Lipids* **2017**, *206*, 28–42, DOI: 10.1016/j.chemphyslip.2017.05.009.

(56) Sengupta, A.; Li, Z.; Song, L. F.; Li, P.; Merz, K. M. J. Parameterization of Monovalent Ions for the OPC3, OPC, TIP3P-FB, and TIP4P-FB Water Models. *J. Chem. Inf. Model.* **2021**, *61*, 869–880, DOI: 10.1021/acs.jcim.0c01390.

(57) Carugo, O.; Pongor, S. A normalized root-mean-spuare distance for comparing protein three-dimensional structures. *Protein Sci.* **2001**, *10*, 1470–1473, DOI: 10.1110/ps.690101.

(58) Shitov, Y. Column subset selection is NP-complete. *Linear Algebra Its Appl.* **2021**, *610*, 52–58, DOI: 10.1016/j.laa.2020.09.015.

(59) Antoulas, A. C., *Approximation of large-scale dynamical systems*; Advances in Design and Control; Society for Industrial and Applied Mathematics: Philadelphia, PA, USA, 2005, p 489, DOI: 10.1137/1.9780898718713.

(60) Friedman, M. The Use of Ranks to Avoid the Assumption of Normality Implicit in the Analysis of Variance. *J. Am. Stat. Assoc.* **1937**, *32*, 675–701, DOI: 10.1080/01621459.1937.10503522.

(61) Benjamini, Y.; Hochberg, Y. Controlling the False Discovery Rate: A Practical and Powerful Approach to Multiple Testing. *J. R. Stat. Soc. Ser. B* **1995**, *57*, 289–300, DOI: 10.1111/j.2517-6161.1995.tb02031.x.

(62) Wilcoxon, F. Individual Comparisons by Ranking Methods. *Biometrics Bulletin* **1945**, *1*, 80–83, DOI: 10.2307/3001968.
